# The Naturally Processed CD95L Elicits a c-Yes/Calcium/PI3K-Driven Cell Migration Pathway

**DOI:** 10.1371/journal.pbio.1001090

**Published:** 2011-06-21

**Authors:** Sébastien Tauzin, Benjamin Chaigne-Delalande, Eric Selva, Nadine Khadra, Sophie Daburon, Cécile Contin-Bordes, Patrick Blanco, Jacques Le Seyec, Thomas Ducret, Laurent Counillon, Jean-François Moreau, Paul Hofman, Pierre Vacher, Patrick Legembre

**Affiliations:** 1 Université de Rennes-1, Rennes, France; 2 IRSET/EA-4427 SeRAIC, Rennes, France; 3 CNRS UMR 5164, Bordeaux, France; 4 Université de Bordeaux-2, Bordeaux, France; 5 Université de Nice Sophia antipolis, INSERM ERI21/EA 4319, Nice, France; 6 CHU Bordeaux, Bordeaux, France; 7 INSERM U1045, Centre de Recherche Cardio-Thoracique de Bordeaux, Bordeaux, France; 8 Université de Nice-Sophia Antipolis, UMR 6097 Faculté des Sciences Parc Valrose, Nice, France; 9 CHU de Nice et Centre de Ressources Biologiques-Tumorothèque, Nice, France; 10 INSERM U916, Institut Bergonié, Bordeaux, France; St. Jude Children's Research Hospital, United States of America

## Abstract

Patients affected by chronic inflammatory disorders display high amounts of soluble CD95L. This homotrimeric ligand arises from the cleavage by metalloproteases of its membrane-bound counterpart, a strong apoptotic inducer. In contrast, the naturally processed CD95L is viewed as an apoptotic antagonist competing with its membrane counterpart for binding to CD95. Recent reports pinpointed that activation of CD95 may attract myeloid and tumoral cells, which display resistance to the CD95-mediated apoptotic signal. However, all these studies were performed using chimeric CD95Ls (oligomerized forms), which behave as the membrane-bound ligand and not as the naturally processed CD95L. Herein, we examine the biological effects of the metalloprotease-cleaved CD95L on CD95-sensitive activated T-lymphocytes. We demonstrate that cleaved CD95L (cl-CD95L), found increased in sera of systemic lupus erythematosus (SLE) patients as compared to that of healthy individuals, promotes the formation of migrating pseudopods at the leading edge of which the death receptor CD95 is capped (confocal microscopy). Using different migration assays (wound healing/Boyden Chamber/endothelial transmigration), we uncover that cl-CD95L promotes cell migration through a c-yes/Ca^2+^/PI3K-driven signaling pathway, which relies on the formation of a CD95-containing complex designated the MISC for *Motility-Inducing Signaling Complex*. These findings revisit the role of the metalloprotease-cleaved CD95L and emphasize that the increase in cl-CD95L observed in patients affected by chronic inflammatory disorders may fuel the local or systemic tissue damage by promoting tissue-filtration of immune cells.

## Author Summary

The “death receptor” CD95 (also known as Fas) plays an essential role in ensuring immune tolerance of self antigens as well as in the elimination of the body's cells that have been infected or transformed. This receptor is engaged by the membrane-bound ligand CD95L, which can be released into blood circulation after cleavage by metalloproteases. Hitherto, most of the studies on the CD95 signal have been performed with chimeric CD95Ls that mimic the membrane-bound ligand and exhibit a level of aggregation beyond that described for the metalloprotease-cleaved ligand. Multi-aggregated CD95L elicits a caspase-driven apoptotic signal. In this study, we observe that levels of soluble and naturally processed CD95L in sera of patients suffering from lupus correlate with disease severity. Strikingly, although this soluble CD95L fails to trigger cell death unlike its chimeric version, it induces a “non-canonical” Ca^2+^/c-yes/PI3K-dependent signaling pathway that promotes the transmigration of T-lymphocytes across the endothelial barrier. These findings shed light on an entirely new role for the soluble CD95L that may contribute to local or systemic tissue damage by enhancing the infiltration of activated T-lymphocytes. Overall, these findings underline the importance of revisiting the role of this “apoptotic cytokine” in the context of chronic inflammatory disorders.

## Introduction

Screening for death inducers led to the discovery of the receptor CD95, which was initially cloned and described as a death receptor belonging to the TNF-R family [1],[2]. Since CD95 is devoid of enzymatic activity, both aggregation [3] and conformational modification [4] of the receptor are crucial to recruit the adaptor protein FADD, which in turn aggregates initiator caspases-8 and -10. On binding of CD95L to CD95, the death receptor clusterized and polarized in a structure designated the CD95-CAP [5],[6]. The local concentration of caspases is followed by their cleavage and the release in the cytoplasm of active caspases culminating in the induction of an apoptotic signal [7]. We and others showed that depending on the extent of CD95 mobilization, it is possible to convert CD95 into a non-apoptotic signaling receptor [8]–[10].

Expression of the CD95 ligand, CD95L, is tightly controlled and is found at the plasma membrane of activated T-lymphocytes and NK cells where it participates in immune surveillance and in peripheral tolerance [11]. As a consequence, failure of the CD95/CD95L signalling leads to auto-immunity [12] and increased risk of cancer [13],[14].

Recent evidence pinpointed that CD95L behaves as a chemoattractant molecule for neutrophils and macrophages [15]–[17] and for malignant cells in which the CD95-mediated apoptotic signal is non-productive [18],[19]. Nonetheless, the biological role of CD95L has to be clarified due to the fact that physiologically, the ligand is present under two main forms with different stoichiometries. Whereas the membrane-bound CD95L induces a potent apoptotic signal, the physiological and pathological role of the metalloprotease-cleaved CD95L remains poorly defined. Indeed, most studies on CD95L are focused on membrane-bound or engineered CD95L, which multimerizes and thereby efficiently triggers cell death. Initial studies focusing on the apoptotic signal induced by CD95 showed that the homotrimeric CD95L behaves as a competitive inhibitor of its membrane-bound counterpart [20],[21]. More recently, the antagonist role of cleaved CD95L (cl-CD95L) has been challenged by Strasser's group [22]. Indeed, they found that ectopic expression of the soluble CD95L in a CD95L-deficient mouse strain dramatically aggravated the lupus-like disease and the occurrence of histiocytic sarcoma compared to mice^gld/gld^ expressing a mutated CD95L incapable of CD95 binding. As a consequence, we may envision that soluble CD95L promotes autoimmunity and tumorigenesis through unknown non-apoptotic activities [22].

Cell migration is a multifactorial and multi-step process involving the formation of lamellipodia/pseudopodia, cell body contraction, and tail retraction [23]. For instance, production of phosphatidylinositol-(3,4,5)-trisphosphate (PIP3), formation of actin mesh, and localized Ca^2+^ rise are common features of migrating cells [23],[24]. Class I PI3K phosphorylates plasma membrane phospholipids and generates the second messenger PIP3, which serves as docking sites for various signaling factors. The cytosolic serine-threonine kinase Akt binds PIP3 via its pleckstrin homology domain (PH domain) and thus redistributes to the plasma membrane. Once at the membrane, Akt is activated through phosphorylation on two sites by the PI3K-dependent kinase-1 (PDK1) on Thr^308^ and by mTOR (mammalian target of rapamycin) complex-2 on Ser^473^.

Chronic inflammatory disorders such as systemic lupus erythematosus (SLE) achieve massive infiltration of organs with cells from innate (neutrophils/macrophages) and adaptive (activated T-lymphocytes) immune systems [25]. The initial steps involved in the attraction of these cells remains to be deciphered. Herein, we ascertain that the naturally processed CD95L is increased in SLE patients and this cytokine does not induce the “orthodox” apoptotic signal but rather ignites a c-yes/Ca^2+^/PI3K signaling pathway, which promotes cell migration.

## Results

### The Metalloprotease-Cleaved CD95L Does Not Induce Caspase-8 Activation and Cell Death

Physiologically, the plasma membrane homotrimeric ligand is released after cleavage of its ectodomain by metalloproteases between either serine 126 and leucine 127 [20] or lysine 129 and glutamine 130 [26]. Since a recent study described that the soluble form may contribute to the aggravation of a lupus-like disease in mice [22], we explored the effect of cl-CD95L on activated lymphocytes. To produce cleaved CD95L (cl-CD95L), we transfected the epithelial kidney cells 293 with wild type CD95L-encoding cDNA. These cells secrete exosomes, which contain full-length CD95L, and thus contaminate supernatants. Therefore, to eliminate this potential contaminant, supernatants from wild type CD95L-transfected 293 cells underwent an ultracentrifugation step to pellet the secreted vesicles [20]. As expected, CD95L-transfected human epithelial kidney 293 cell line produced full-length plasma membrane-bound CD95L (≈40 kDa) (Figure S1A) and pelleted exosomes also contained full-length CD95L (Figure S1A). Cleaved-CD95L was found in exosome-free supernatant at a lower molecular weight (≈30 kDa) than the full-length ligand (Figure S1A). In contrast, supernatants harvested from 293 cells expressing a CD95L in which serine 126 and leucine 127 were replaced by glutamic acid (CD95L^S126E/L127E^) [20], the putative cleavage site of metalloprotease, exhibited a dramatic decrease in their amounts of soluble CD95L, confirming that CD95L was cleaved between these amino acids (Figure S1A). The faint amount of soluble CD95L found in CD95L^S126E/L127E^-transfected 293 cells may be due to the presence of another cleavage site (i.e., Lys129/Gln130) since a shift of this cleavage site has also been described [26].

Next, we confirmed the homotrimeric stoichiometry of the cleaved CD95L using gel filtration (Figure S1B). In the purpose of generating a multi-aggregated and highly cytotoxic ligand, several CD95L-containing constructs have been engineered. As already reported, we have generated a dodecameric CD95L (gel filtration analysis, unpublished data) through the fusion of the extracellular region (a.a. 105 to 281) of CD95L with a dimerization domain (Ig) derived from the LIF receptor gp190 [27]. Although the Ig-CD95L triggered induction of the initiator caspase-8 and cell death (Figure S1C and S1D), cl-CD95L failed to initiate either cleavage of caspase-8 (Figure S1C) or apoptosis (Figure S1D). Overall, these findings support the conclusion that in contrast to the multi-aggregated forms of CD95L, which mimic the membrane-bound ligand, homotrimeric cl-CD95L does not achieve induction of the “orthodox” CD95-mediated apoptotic signal.

### Cleaved-CD95L Induces the Formation of CD95-CAP

**Figure 1:**
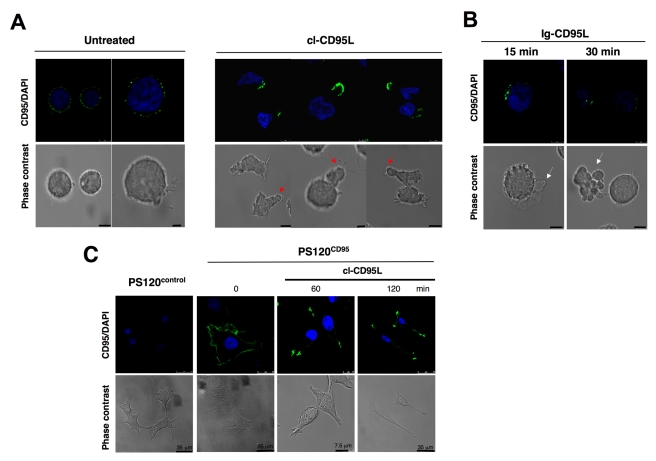
Cl-CD95L elicits CD95-CAP formation. (A) Activated (PHA/IL2)-peripheral blood T-lymphocytes (PBLs) from healthy individuals were incubated with or without 100 ng/ml of cl-CD95L for 30 min. Cell morphology was followed using phase contrast microscopy and CD95 was stained using a mouse anti-CD95 mAb (APO1-3) followed by a goat Alexa488-coupled anti-mouse IgG mAb. Red arrows depict emitted pseudopods (Bars = 5 µm). (B) PHA/IL2-activated PBLs were incubated with the cytotoxic Ig-CD95L for indicated times, and cells were analyzed as described in (A). White arrows depict blebs emitted by apoptotic cells (bars = 5 µm). (C) The fibroblastic cell line PS120 devoid of endogenous CD95 (PS120^control^) or reconstituted with human wild type CD95 (PS120^CD95^) were untreated or treated with 100 ng/ml of cleaved CD95L for indicated times, and cell shape and CD95 distribution were analyzed.

It has been described that soluble CD95L acts as an inert ligand, which once bound to CD95 competes with the membrane-bound CD95L and prevents the transmission of the apoptotic signal [20],[21]. Consistent with this statement, cells incubated with cl-CD95L did not undergo caspase-8 activation or cell death (Figure S1C and S1D). Nevertheless, cl-CD95L was able to induce proximal events of the CD95 signal up to the formation of CD95-CAP (Figure 1A). Strikingly, once incubated with 100 ng/ml (3.3 nM) of cl-CD95L, classical round-shaped activated T-lymphocytes underwent a dramatic alteration of their morphology and emitted pseudopods (red arrows, Figure 1A). Formation of CD95-CAP and pseudopods in the presence of cl-CD95L was also observed with the leukemic T-cell lines H9 and Jurkat (Figure S2A–C). In contrast, incubation of activated lymphocytes with the dodecameric Ig-CD95L triggered cell blebbing and shrinkage, hallmarks of cells dying through an apoptotic process (Figure 1B). Alteration of cell morphology and formation of CD95-CAP was not restricted to hematological cells since the fibroblastic PS120^CD95^ cell line, derived from PS120, a CD95-deficient cell that has been reconstituted with wild type human CD95 (Figure S3A), also underwent alterations of its morphology in the presence of cl-CD95L (Figure 1C). Indeed flat, cubical epithelial shape was converted to bipolar, well-oriented, slanted, fibroblastic appearance following incubation with 100 ng/ml of cl-CD95L (Figure 1C). Similarly to lymphocytes, clustering of CD95 was observed at the extremity of the emitted pseudopods in the fibroblastic cell line (Figure 1C).

### Cleaved-CD95L Induces the Emission of PIP3/Ca^2+^/F-Actin-Containing Pseudopods

The initial response of a cell to a migration-promoting agent is to emit protrusions in the direction of migration. These protrusions can be large, broad lamellipodia or more slender and filiform structures such as pseudopods and are usually driven by actin polymerization [23]. Several studies established that an intracellular calcium rise and an activation of PI3K play a crucial role to achieve cell motility [28]–[31]. These signals contribute to the remodeling of actin cytoskeleton, which in association with myosin motors elicits the mechanical process of cell movement. We then investigated whether although unable to trigger caspase activation, cl-CD95L induced remodeling of actin, Ca^2+^ response, and activation of the PI3K signaling pathway.

**Figure 2:**
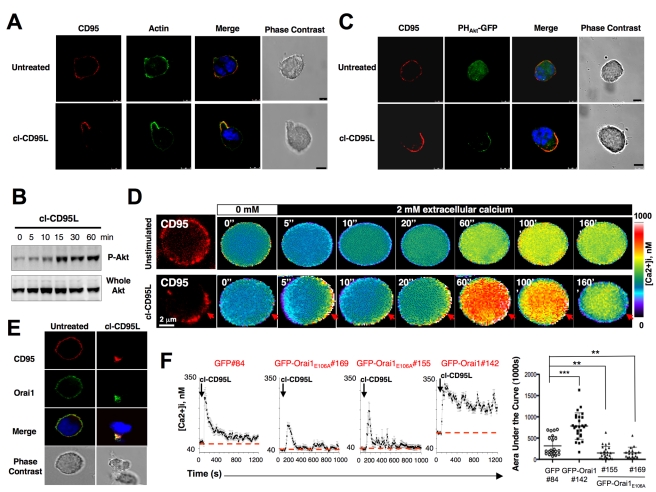
Cleaved CD95L triggers a pseudopod-localized actin/PI3K/Akt signal and a Ca^2+^ entry through activation of the CRAC channel Orai1. (A) H9 T-cells were transiently transfected with the actin marker Lifeact-GFP. Living cells were harvested (Ficoll) and treated or untreated for 30 min with 100 ng/ml of cleaved CD95L. Cells were fixed, and CD95 was stained using anti-CD95 mAb (APO1-3) and revealed with the secondary Alexa555-coupled Goat anti-mouse antibody (red). Nuclei were stained using DAPI (blue). Bars = 5 µm. Images were acquired with an ApoPLAN 63× objective. (B) The leukemic T-cell line H9 was incubated with 100 ng/ml of the naturally processed CD95L for indicated times. Cells were lysed, and for each lane, 100 µg of protein were loaded. Proteins were resolved by SDS-PAGE and anti-whole Akt and anti-Akt phosphorylation (serine^473^) immunoblots were performed. Akt phosphorylation stands for its activation. (C) The pleckstrin homology (PH) domain of Akt binds PIP3 produced by PI3K activation. As a consequence, the probe PH_Akt_-GFP stains PIP3. H9 T-cells were transiently transfected with the PH_Akt_-GFP construct (green). Living cells were isolated (Ficoll) and incubated with 100 ng/ml of the naturally processed CD95L (cl-CD95L) for 30 min. Cells were fixed, and CD95 was stained as previously mentioned (red). Pictures were taken by confocal microscopy. Cell morphology was followed using differential interference contrast microscopy. Nuclei were stained with DAPI (blue). Bars = 5 µm. (D) *Calcium measurement in single cell*. Jurkat T-cells were loaded with the permeant calcium probe Fura-2AM, and in parallel, CD95 was followed using an anti-CD95 mAb and an Alexa555-coupled goat anti-mouse mAb as described in Materials and Methods. Cells were bathed in a Ca^2+^-free medium and pre-incubated (*lower panel*; cl-CD95L) or not (*upper panel*; unstimulated) with cl-CD95L (100 ng/ml). 2 mM Ca^2+^ was added when indicated by the black-filled rectangle. Ratio images (F340 nm/F380 nm) were taken every 5 s, and the images shown correspond to the indicated period of time. Grey level intensities were translated to false colors according to the colors scale shown at the right, and [Ca^2+^]_i_ was estimated from the ratio values and calibration experiments as described in Materials and Methods. Red arrows indicate the CD95-CAP. (E) Jurkat T-cells were treated or untreated with 100 ng/ml of cl-CD95L for 60 min. Cells were fixed and Orai1 and CD95 were stained as described in Materials and Methods. Cell morphology was analyzed using phase contrast microscopy. (F) *Left panels*: GFP-, GFP-Orai1, or GFP-Orai1_E106A_-expressing Jurkat T-cells were loaded with 1 µM of the calcium probe Fura-2AM for 30 min at RT. Cells were bathed at 37°C in a medium containing 2 mM [Ca^2+^]_e_ and then treated with 100 ng/ml of cl-CD95L (black arrow). GFP#84 corresponded to control cells expressing GFP. GFP-Orai1_E106A_#155 and #169 corresponded to two independently isolated clones expressing the non-functional CRAC channel. GFP-Orai1#142 corresponded to Jurkat cells overexpressing human Orai1. Values were recorded every 10 s. *Right panel*: For each experiment, the area under the curve (AUC) was measured for 1,000 s, and the statistical analyses of the AUC values were performed for indicated cells using non-parametric two-tailed Mann-Whitney tests. ** and *** indicate *p* values≤0.01 and 0.001, respectively.

Using the non-competing molecule Lifeact, a small peptide, which has recently been described to bind polymerized actin and to not interfere with actin dynamics [32], we clearly observed that the pseudopods emitted by leukemic T-cells concentrated a dense meshwork of polymerized actin (Figure 2A). The serine-threonine kinase Akt is a downstream effector of the PI3K signal and its phosphorylation on its serine^473^ is a key feature of its activation. As shown in Figure 2B, T-cell lines incubated with cl-CD95L underwent a potent Akt phosphorylation. To confirm that cl-CD95L behaved as an inducer of the PI3K/Akt signal, we analyzed the impact of the naturally processed ligand on the production of the plasma membrane PIP3. For this purpose, we expressed in T-cells a chimeric protein encompassing GFP fused to the pleckstrin homology domain of Akt (PH_Akt_-GFP), which possesses a strong affinity to the negatively charged lipid. Addition of cl-CD95L on H9 T-cells promoted production of PIP3 since the cytoplasmic localization of PH_Akt_-GFP in resting cells changed for a plasma membrane reorganization (Figure 2C). Overall these findings highlighted that the naturally processed CD95L activates the PI3K/Akt signaling pathway.

On the grounds that spatiotemporal distribution of “calcium microdomains” orchestrates directional movement during cell migration [33], we further investigated the effects of cl-CD95L on the intracellular distribution of Ca^2+^ using video-microscopy and we established that T-lymphocytes incubated with cl-CD95L revealed significantly higher Ca^2+^ concentration at the leading edge of the pseudopod (Figure S4), beneath the CD95-CAP (Figure 2D).

Activation of a wide variety of plasma membrane receptors elicits a rapid biphasic Ca^2+^ response through the induction of phospholipase C (PLC) that generates inositol 1,4,5-trisphosphate (IP3), which in turn activates the IP3-receptors present in the membrane of the endoplasmic reticulum (ER). IP3-R activation drives the release of the ER-stored Ca^2+^ [34]. The second phase of the Ca^2+^ signal is mainly achieved by activation of the store-operated Ca^2+^ (SOC) channels in the plasma membrane that induce a Ca^2+^ influx [35]. In order to address the cl-CD95L-mediated molecular mechanism evoking the Ca^2+^ response, we first investigated whether PLCγ1 participated in this signal. Phosphorylation of PLCγ1 on its tyrosine 783 indicated that the lipase was activated in the presence of cl-CD95L (Figure S5A). To prove that PLCγ1 participated in the cl-CD95L-mediated Ca^2+^ rise, we analyzed the cl-CD95L-mediated Ca^2+^ signal in PLCγ1-deficient T-cells (Figure S5B). The Ca^2+^ signal was abrogated in the PLCγ1-deficient T-cells, while it was restored in the PLCγ1-reconstituted counterpart (Figure S5B). Second, T-cells pre-incubated with the IP3-R inhibitors, 2-APB (Figure S5C and S5D) and xestospongin C (unpublished data), failed to mobilize Ca^2+^ in the presence of cl-CD95L, indicating that the homotrimeric ligand elicited the Ca^2+^ signal through activation of PLCγ1 and the subsequent induction of the IP3-receptors.

In nonexcitable cells, SOC influx is the major Ca^2+^ entry mechanism [35],[36]. Recently it has been ascertained that two genes, STIM1 (stromal interaction molecule 1) and Orai1, participate in the SOC entry [37]–[41]. While STIM1 behaves as an ER-localized Ca^2+^ sensor, Orai1 is a pore-forming component of the SOC channel [42],[43]. To finely identify the molecular mechanism that drives the Ca^2+^ rise observed in the presence of cl-CD95L, we first explored whether the extracellular Ca^2+^ contributed to the cl-CD95L-mediated Ca^2+^ signal. In T-cell lines bathed in a Ca^2+^-free medium, the cl-CD95L-mediated Ca^2+^ signal was significantly reduced as compared to regular medium (Figure S6A and S6B). These findings suggested that Ca^2+^ influx participated in the cl-CD95L-induced Ca^2+^ response. To investigate the role played by the SOC channels on the cl-CD95L-mediated Ca^2+^ response, we analyzed the effect of the pharmacological inhibitor of SOC channels termed BTP2 [44] on this Ca^2+^ signal. Pre-incubation of peripheral blood lymphocytes (PBLs) with BTP2 reduced the magnitude of the Ca^2+^ peak and abrogated the Ca^2+^ plateau observed in the presence of the metalloprotease-processed cl-CD95L (Figure S6C), suggesting that the SOC channels contributed to Ca^2+^ entry upon the addition of cl-CD95L. Next, the role of Orai1 in the cl-CD95L-driven Ca^2+^ entry was studied. Confocal microscopy revealed that addition of cl-CD95L led to the compartmentalization of Orai1 into the CD95-CAP (Figure 2E). Substitution of the conserved glutamate in position 106 to an alanine (Orai1_E106A_) results in a non-conducting Orai1 channel acting as a dominant negative construct that abrogates the native SOC current [40],[42],[43]. Consequently, we generated leukemic T-cell clones stably expressing the Orai1 mutant or over-expressing the wild type Orai1 and we assessed the Ca^2+^ response induced by cl-CD95L. Compared to GFP-expressing control cells, the ectopic expression of Orai1_E106A_ dramatically reduced the amplitude and the duration of the Ca^2+^ response (Figure 2F). On the other hand, over-expression of Orai1 resulted in a significant increase in the intensity and the duration of the CD95-mediated Ca^2+^ response (Figure 2F). Overall, these findings established that in the presence of cl-CD95L, T-cells elicit a Ca^2+^ response through a PLCγ1/IP3R/Orai1 signaling pathway at the leading edge of the emitted pseudopod in a structure that may contribute to cell migration.

### cl-CD95L Promotes Cell Migration through a Death-Domain Dependent Signal

**Figure 3:**
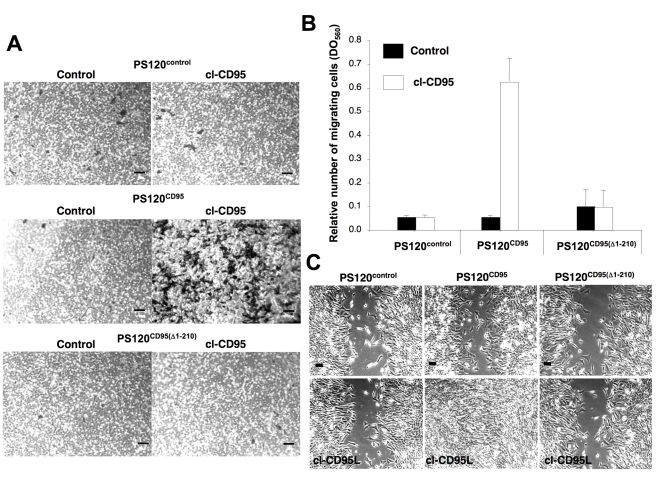
Cleaved CD95L promotes cell migration through a DD-dependent signal. (A) The CD95-deficient PS120 and its counterparts expressing human wild type CD95 or DD-truncated CD95 were seeded in a Boyden chamber in the presence or absence of cl-CD95L (100 ng/ml) and incubated for 24 h. The filter was removed, the upper side containing the non-migrating cells was wiped out with cotton-tipped swabs, and migrating cells in the opposite side of the filter were fixed with methanol and stained (Giemsa). For each experiment, five pictures of random fields were taken and a representative picture was depicted (Bars = 70 µm). (B) Cells were treated as described in (A). To quantify cell motility, Giemsa-stained migrating cells from the lower side of the membrane were lyzed and absorbance was measured at 560 nm. (C) Wound healing assay, a confluent monolayer of the indicated cells was “wounded” with a tip and then cells were incubated for 24 h in the presence or absence of 100 ng/ml of cl-CD95L and pictures were acquired (Bars = 50 µm). Pictures are representative of 5 independently performed experiments.

Next, we addressed the impact of the metalloprotease-processed CD95L on cell migration by using Boyden chamber and wound healing assays. For this purpose, we reconstituted the CD95-deficient PS120 cell line with either wild type CD95 (PS120^CD95^) or death-domain truncated counterpart (PS120^CD95(Δ1-210)^) (Figure S3A). In contrast to the parental PS120 or PS120^CD95(Δ1-210)^, PS120^CD95^ exhibited a sensitivity towards the multimeric Ig-CD95L (Figure S3B), which indicated that the intracellular CD95 machinery remained functional in these cells. As expected, cl-CD95L did not trigger cell death in the different PS120 cell lines (Figure S3B). Strikingly, we found that in contrast to PS120 and PS120^CD95(Δ1-210)^ cells, migration of PS120^CD95^ cells was stimulated in the presence of cl-CD95L (Figure 3A and 3B), indicating that a functional death domain was required to mediate the cl-CD95L effects on motility. To confirm the role of cl-CD95L on cell motility, wound-healing assays were carried out. Fibroblasts were grown to confluency, and a “wound” was realized in the cell monolayer. While in the presence of cl-CD95L cells devoid of CD95 or expressing a DD-deficient CD95 failed to fill the gap after 24 h, the CD95-reconstituted PS120 completely healed the “wound” (Figure 3C). In addition, incubation with cl-CD95L did not modify the cell proliferation rate (unpublished data). Overall, these findings led to the conclusion that cl-CD95L induces a DD-dependent signal, which promotes migration of the CD95-expressing cells.

### Cl-CD95L Causes an Orai1- and PI3K-Dependent Program of Cell Migration

**Figure 4:**
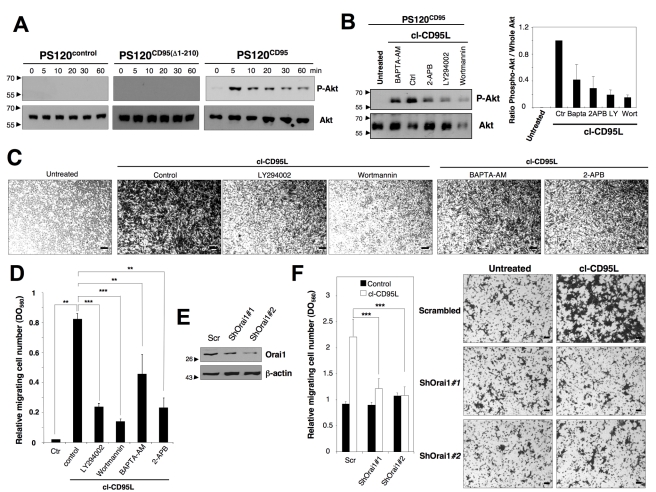
cl-CD95L promotes a cell migration through a Ca^2+^/PI3K/Akt signal. (A) The CD95-deficient fibroblastic cell line PS120 transfected with empty vector (PS120^control^) or reconstituted with either human wild type CD95 (PS120^CD95^) or a death domain-truncated CD95 (PS120^CD95(Δ1-210)^) was incubated with 100 ng/ml of cleaved CD95L for indicated times. Cells were harvested, lyzed, and 100 µg of protein was loaded per lane. Proteins were resolved in a SDS-PAGE and immunoblots were performed. Phosphorylation of the serine^473^ on Akt indicates activation of the kinase. Whole Akt is added as loading control. (B) *Upper panels*: PS120^CD95^ cells were pre-incubated with non-cytotoxic and non-cytostatic concentration of PI3K inhibitors (2.5 µM of wortmannin and 5 µM of LY294002), the cell permeant chelator of calcium BAPTA-AM (5 µM), or the inhibitor of IP3-R and SOC channels 2-APB (20 µM). Cells were then untreated (ctr) or treated for 5 min with 100 ng/ml of cl-CD95L and lysed, 50 µg/ml of protein was loaded per lane, and indicated immunoblots were performed. *Lower panels*: densitometry analyses of the immunoblot bands using ImageJ software and the histograms depict Phospho-Akt/whole Akt ratios. (C) Cell migration of PS120^CD95^ was assessed using the Boyden chamber assay. Cells were pre-incubated with non-cytotoxic and non-cytostatic concentrations of PI3K inhibitors (2.5 µM of Wortmannin and 5 µM of LY294002), the cell permeant chelator of calcium BAPTA-AM (5 µM), or the inhibitor of SOC channels 2-APB (20 µM) for 30 min and then stimulated with 100 ng/ml of cl-CD95L for 24 h. For each experiment, 10 pictures of the migrating cells were taken and a representative picture was depicted (Bars = 50 µm). (D) Cells were treated as described in (C). To quantitatively measure cell motility, Giemsa-stained migrating cells from the lower side of the membrane were lyzed, and absorbance was measured at a wavelength of 560 nm. Values represent means and SD of three independently performed experiments. **p<0.01 and ***p<0.001 as calculated using two-tailed non-parametric Mann-Whitney test. (E) The silencing effect of the Orai1-targeting shRNAmir-pGIPZ vectors was analyzed by immunoblot in lentiviral-transduced HEK cells. 48 h after transduction, cells were lysed and 100 µg of lysates were loaded per line. β-actin serves as a loading control. (F) Cell migration of indicated shRNAmir-transduced HEK cells was assessed using Boyden chamber assay. *Right panels*: HEK cells were transduced with scrambled or Orai1-targeting ShRNA, and 48 h later, cells were incubated for 24 h in the presence or absence of 100 ng/ml of cl-CD95L. Migrating cells were fixed with methanol and stained by Giemsa. For each experiment, five pictures of random fields were taken and a representative picture was depicted (Bars = 70 µm). *Left panel*: To quantitatively measure cell motility, Giemsa-stained migrating cells from the lower side of the membrane were lyzed, and absorbance was measured at a wavelength of 560 nm. Values represent means and SD of three independently performed experiments. *** *p*<0.001 as calculated using two-tailed non-parametric Mann-Whitney test.

As the death domain of CD95 was crucial to mediate the motile signal, we next analyzed whether this intracellular region was also mandatory to induce Akt phosphorylation upon addition of cl-CD95L. We did not observe any Akt phosphorylation in PS120^control^ and PS120^CD95(Δ1-210)^ cells (Figure 4A). Conversely, expression of wild type CD95 restored the transmission of the PI3K/Akt signal upon cl-CD95L addition (Figure 4A). Since interconnections have been reported between Ca^2+^ and PI3K/Akt signaling pathways, we next investigated whether the cl-CD95L-mediated Ca^2+^ response contributed to the magnitude of the Akt activation level. To prevent the cl-CD95L-mediated calcium response, cells were pre-treated with the calcium chelator BAPTA-AM and 2-APB, an inhibitor of inositol 1,4,5-trisphosphate receptor (IP_3_R) and store-operated calcium (SOC) channels. Inhibition of the cl-CD95L-induced Ca^2+^ response using BAPTA-AM or 2-APB decreased by 60% and 70% the amount of Akt phosphorylation (Figure 4B), respectively, indicating that the Ca^2+^ rise observed upon addition of cl-CD95L enhanced the activation level of Akt.

Next, the role played by Ca^2+^/PI3K signals in the CD95L-mediated cell motility was investigated. Non-cytotoxic and non-cytostatic amounts of the PI3K inhibitors LY294002, a quercetin analogue [45], or Wortmannin, a fungal metabolite, dramatically reduced the cl-CD95-mediated cell motility (Figure 4C and 4D). Likewise, BAPTA-AM and 2-APB, which both prevented the cl-CD95L-mediated Ca^2+^ response (Figure S5C and S5D, S7A and S7B), significantly impeded cell migration (Figure 4C and 4D). Indeed, we found that inhibition of PI3K activity by LY294002 and Wortmannin reduced cell motility by 75% and 83%, respectively. On the other hand, down-modulation of the CD95-mediated Ca^2+^ response by either BAPTA-AM or 2-APB achieved 47% and 71% of cell migration blockade, respectively. We next examined the effects of small-molecule inhibitors of class I PI3Ks to explore the contribution of the different isoforms in the cl-CD95L-mediated cell migration. While the catalytic subunits p110α and p110β are ubiquitously expressed, p110δ and p110γ are found predominately in hematological cells [46]. By screening for p110 isoform-selective PI3K inhibitors (Figure S8A) that could prevent the cl-CD95L-mediated cell migration, we observed that whereas p110-γ was instrumental in both the Akt activation and the transmigration of H9 T-cells across a barrier of endothelial cells (Figure S8B and S8D), the α and β isoforms orchestrated the motility process in the non-hematological cell line PS120^CD95^ (Figure S8C and S8E). These findings definitively proved that the cl-CD95L-mediated motility signal occurred through the class I PI3K activation and that the implicated isoform varied between hematological and non-hematological cells. Finally, we wondered whether Orai1 contributed to the cl-CD95L-mediated cell migration. To address this question, HEK cells were transiently transfected with either scrambled or Orai1-targeting shRNA (Figure 4E). Orai1 knock-down in HEK cells reduced the SOC current by 85% [47]. Strikingly, the silencing of Orai1 abrogated the cl-CD95L-mediated cell migration (Figure 4F), strongly supporting the conclusion that the Orai1-driven Ca^2+^ entry contributed to the motility signal induced in the presence of cl-CD95L. Altogether, these findings underlined that the metalloprotease-cleaved CD95L does not behave as an inert ligand but rather stimulates cell migration through an Orai1/Ca^2+^/PI3K-dependent mechanism.

### Cleaved CD95L Activates the PI3K/Akt Signal Through the src Kinase c-Yes

**Figure 5:**
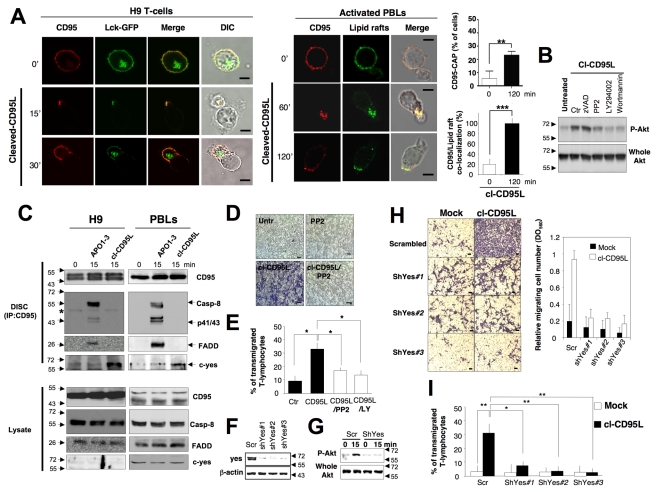
The src kinase c-yes orchestrates the cl-CD95L-mediated PI3K/Akt signaling pathway and cell migration. (A) *Upper panels*: T-leukemic cell line H9 was transiently transfected with Lck-GFP. 24 h after transfection, living cells were treated or untreated for the indicated times in the presence of cl-CD95L (100 ng/ml). CD95 was stained using a mouse anti-CD95 mAb and a goat Alexa555-coupled anti-mouse IgG mAb. *Lower panels*: Activated PBLs were incubated for the indicated times with 100 ng/ml of cl-CD95L, and then cells were fixed. Lipid rafts were stained using Alexa488-cholera toxin B subunit and CD95 was followed as previously mentioned. Images were acquired with a confocal microscope with a 63× objective. Cell morphology was followed using phase contrast microscopy (Bars = 7.5 µm). The percentage of T-cells displaying a CD95 cluster was assessed (∼300 cells counted for each condition). Among the activated T-lymphocytes showing CD95 cluster, the amount of CD95-CAP co-localized with lipid rafts was assessed. (B) H9 T-cells were pre-incubated for 30 min with DMSO (U), 10 µM of PP2, 5 µM of LY294002 (LY), 2.5 µM of wortmannin (Wort), or 40 µM of zVAD-fmk (zV) and then treated or untreated for 15 min with 100 ng/ml of cl-CD95L. Cells were lyzed and Akt phosphorylation (S^473^) and whole Akt were assessed by immunoblots. (C) The H9 T-cell line (*left panels*) and activated PBLs (*right panels*) were incubated for indicated times with 100 ng/ml of cl-CD95L or APO1-3. Then, cells were lyzed and CD95 was immunoprecipitated. The immune complex was resolved in a 10% SDS-PAGE, and indicated immunoblots were performed. Total lysates were depicted to confirm that the same amount of protein was present for each immunoprecipitation. The black stars represent heavy chains of Ig. (D) Boyden chamber assays were performed as described in Materials and Methods. PS120^CD95^ were pre-incubated with or without a non-cytotoxic and non-cytostatic concentration of PP2 (10 µM) for 30 min and then treated (cl-CD95L) or untreated (−) for 24 h with 100 ng/ml of cl-CD95L. Data are representative of three independently performed experiments. (E) Activated PBLs were pre-incubated with 10 µM of the src inhibitor PP2 or 5 µM of the PI3K inhibitor LY294002 (LY) and then incubated in the presence or absence of cl-CD95L (100 ng/ml) for 24 h. Then, endothelial transmigration of PBLs was assessed as described in Materials and Methods. (F) The silencing effect of the c-yes-targeting shRNAmir-pGIPZ vectors was analyzed by immunoblot in lentiviral-transduced PS120^CD95^ cells. 72 h after transduction, cells were lysed and 100 µg of lysate was loaded per line. β-actin serves as loading control. (G) The T-cell line H9 was transduced with scramble (scr) or c-yes (shYes#3)-targeting shRNAmir-containing lentivirus. 72 h after transduction, living cells were harvested and green cells were sorted by flow cytometry using FACSAria. Cells were immediately stimulated or unstimulated with cl-CD95L (100 ng/ml), and the amounts of Akt phosphorylation (S^473^) were assessed by immunoblot. (H) Cell migration of indicated shRNAmir-transduced PS120^CD95^ was assessed using the Boyden chamber assay. *Left panels*: 72 h after transduction, cells were treated (cl-CD95L) or untreated (mock) with 100 ng/ml of cl-CD95L for 24 h. For each experiment, five pictures of the migrating cells were taken and a representative picture was depicted (Bars = 50 µm). *Right panel*: To quantitatively measure cell motility, Giemsa-stained migrating cells from the lower side of the membrane were lyzed and absorbance was measured at a wavelength of 560 nm. Values represent means and SD of three independently performed experiments. (I) H9 cells were transduced as mentioned in (G), and the green cells were incubated in the presence or absence of cl-CD95L (100 ng/ml) for 24 h. Then, the endothelial transmigration of the sh-RNA-expressing T-cells was assessed as described in Materials and Methods.

Induction of the CD95-mediated PI3K/Akt response remains poorly defined. However, it is established that activation of PI3K can be reached through activation of src kinases [48], which are found enriched into sub-domains of the plasma membrane designated lipid rafts [49]. In this regard, src kinases are anchored to lipid rafts through the double acylation (i.e., palmitoylation and myristoylation) of their amino-terminal sequence [50]. Lipid rafts were tagged using the amino-terminal domain of the src kinase Lck fused to the fluorescent protein GFP [51], and we examined whether cl-CD95L enabled the compartmentalization of CD95 into lipid rafts, meaning in close vicinity of the src kinases. While T-cells displayed a homogeneous distribution of the Lck-GFP probe at the plasma membrane, addition of cl-CD95L partitioned CD95 into lipid rafts at the leading edge of the pseudopod (Figure 5A). To follow lipid rafts in activated PBLs, we incubated cells with a fluorescent-conjugated cholera toxin B sub-unit that exhibits strong affinity for the monosialoganglioside GM1, a lipid enriched into lipid rafts [52]. We confirmed that cl-CD95L achieved the partition of CD95 into lipid rafts at the leading edge of the emitted pseudopod in activated PBLs (Figure 5A). In addition, inhibition of Src kinase activity using the pharmacologic inhibitor PP2 impeded the phosphorylation of Akt, supporting the notion that src kinases were involved in the transduction of the CD95-mediated PI3K/Akt signal (Figure 5B). C-yes belongs to the src family and its activity promotes activation of PI3K in glioblastoma cells [19]. Strikingly, in activated PBLs and in the T-cell line H9, we observed that in contrast to the “canonical DISC” formed in the presence of the agonistic antibody APO1-3, addition of cl-CD95L failed to induce the binding of FADD, caspases-8 (Figure 5C), and -10 (unpublished data) to CD95 but promoted the recruitment of the tyrosine kinase c-yes (Figure 5C). In contrast, another src kinase termed syk, which is involved in the BCR (B-cell receptor)-mediated PI3K activation [48], was not detected in the CD95-containing complex (unpublished data). These findings indicated that binding of cl-CD95L to CD95 reached the formation of a molecular complex devoid of the initiator caspases-8 and -10. In agreement with these latter observations, inhibition of the caspase activity using the broad-spectrum caspase inhibitor zVAD-fmk did not alter Akt phosphorylation (Figure 5B) and cell motility (Figure S9A and S9B). Next, the role of src kinases in the cl-CD95L-mediated cell motility was explored. Using Boyden chamber and wound healing assays, we demonstrated that the src kinase inhibitor PP2 completely abrogated the cl-CD95L-mediated cell motility (Figure 5D and S10A). Likewise, activated PBLs underwent a significant increase in their migration across a vascular endothelial monolayer in the presence of cl-CD95L as compared with control medium and both PP2 and LY294002 hindered the transmigration process (Figure 5E). To ascertain that c-yes participated in igniting the CD95-mediated motility signal, cells were transduced with c-yes shRNA-encoding lentiviral vectors. (Figure 5F). C-yes silencing prevented the cl-CD95L-mediated Akt activation (Figure 5G) and thus abrogated both the cell migration of the non-hematological cell line PS120^CD95^ (Figures 5H and S9B) and the transmigration of T-cells across endothelial cells (Figure 5I). Overall, these findings provide unique evidence that the naturally processed CD95L elicits a non-apoptotic signal through the formation of a “non-canonical” DISC devoid of FADD and caspases-8/-10 but encompassing the tyrosine kinase c-yes. This newly disclosed molecular complex was designated MISC for motility-inducing signaling complex.

### Incubation of Activated Lymphocytes with Sera of SLE Patients Induces Endothelial Adhesion and Transmigration

**Figure 6:**
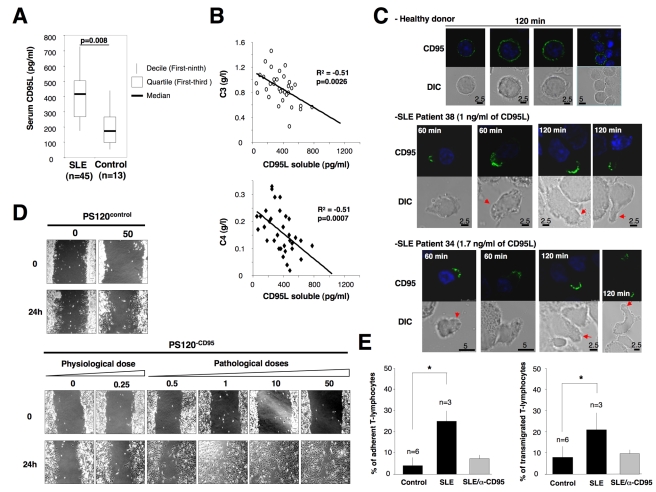
CD95L found in SLE patients promotes endothelial transmigration of activated PBLs. (A) By ELISA, the amounts of soluble CD95L in sera of healthy donors (control) and SLE patients were quantified. *n* stands for the number of SLE patients and healthy subjects. (B) Correlation between aggravation markers of the disease and the amount of soluble CD95L. (C) Activated PBLs were incubated for the indicated times with sera of healthy donors or SLE patients and the distribution of CD95 was analyzed by confocal microscopy. (D) A 24 h wound healing assay was performed. The fibroblastic cell line PS120^control^ or its CD95-expressing counterpart PS120^CD95^ was grown to confluence. then the monolayer was “wounded” by scratching cells using a pipette tip. Using microscopy, the migration was monitored for 24 h with the indicated concentrations of cl-CD95L as indicated in Materials and Methods. Pictures are representative of 3 independently performed experiments. (E) Activated PBLs were incubated in the presence of sera from SLE patients (*n* = 3) or healthy donors (*n* = 6), and then, adhesion to endothelial cells (*left panel*) and endothelial transmigration (*right panel*) of PBLs were assessed as described in Materials and Methods. Where indicated, activated PBLs were preincubated 30 min with the antagonist anti-CD95 mAb ZB4 (α-CD95). ZB4 was maintained in the culture at 10 µg/ml.

Since in murine model of systemic lupus erythematosus (SLE) ectopic expression of CD95L leads to a dramatic exacerbation of the disease index, we wondered whether cleaved CD95L was found increased in SLE-affected patients as compared to healthy individuals. We observed that the CD95L level was significantly higher in sera from SLE patients (431.28±301.8 pg/ml) than in healthy donors (217.12±125.8 pg/ml) (*p* = 0.008) (Figure 6A). Furthermore, we showed that the level of soluble CD95L was inversely correlated with two markers of the disease activity, complement C3 (R^2^ = 0.51, *p* = 0.0026) and C4 (R^2^ = 0.51, *p* = 0.0007) (Figure 6B). In this regard, we propose that serum CD95L may serve as a solid surrogate marker for the inflammatory progression of the disease in SLE patients.

Next, to address whether CD95L found in sera of SLE patients behaves similarly to the one produced in vitro, we incubated activated PBLs with control or SLE sera and analyzed cell morphology by contrast phase and plasma membrane distribution of CD95 using confocal microscopy. Sera from healthy donors induced neither clustering of CD95 at the plasma membrane nor alteration of the cell morphology, whereas sera from lupus patients dramatically aggregated CD95 at the plasma membrane of activated lymphocytes. Similar to observations obtained with cl-CD95L produced in 293 cells, CD95-CAP was found distributed at the extremity of the emitted pseudopod (see red arrows in Figure 6C). In addition, both modification of cell morphology and formation of CD95-CAP were abrogated by pre-incubating cells with anti-CD95 blocking mAb (clone ZB4, unpublished data).

To decipher whether the increased amount of CD95L found in SLE patients compared to healthy individuals was able to trigger cell motility, titration of CD95L was applied on PS120 and PS120^CD95^ and motility was assessed using wound healing assay. Doses of cleaved CD95L as low as 500 pg/ml efficiently promoted cell migration of PS120^CD95^ cells, while 250 pg/ml of CD95L was totally inefficient to enhance the basal motile pattern of these cells (Figure 6D). As previously mentioned, cells underwent migration through a CD95-driven mechanism, since CD95-deficient PS120 did not occur in cell migration upon addition of cleaved-CD95L (Figure 6D). Interestingly, 500 pg/ml of soluble CD95L was around the median concentration measured in SLE patients (431 pg/ml), while 250 pg/ml corresponded to the median concentration found in healthy donors (253 pg/ml) (Figure 6A). Finally, to establish that cleaved-CD95L promotes extravasation of activated T-lymphocytes, a process that is instrumental in accumulating T-cells in inflamed tissues and thus in fuelling the inflammatory process, activated T-lymphocytes were incubated either with SLE or healthy sera and both adhesion on and transmigration across endothelial cells were quantified. In the presence of SLE sera, activated T-lymphocytes underwent a significant increase in their adherence (4±1.21 versus 2±0.4×10^4^ cell equivalent, for SLE versus control sera, *p*<0.05) (Figure 6E) and migration across a vascular endothelial monolayer (Figure 6E) as compared with T-lymphocytes incubated in sera from healthy donors. In addition, blocking the CD95/CD95L interaction with an anti-CD95 neutralizing mAbs (clone ZB4) impeded both adhesion and transmigration of the activated T-cells. In summary, these findings indicated that the high amounts of cleaved-CD95L found in lupus patients promote T-lymphocyte extravasation and accumulation of the immune cells in inflamed tissues.

## Discussion

Most of the experiments aiming to understand the biological functions of CD95L have focused on its transmembrane moiety or engineered soluble CD95L mimicking the multimerized membrane-bound ligand. However, using the physiological soluble CD95L, which is the result of its membrane shedding, we demonstrated for the first time, to our knowledge, that this cytokine released in the circulation behaves as a potent inducer of non-canonical (caspase-independent) and non-apoptotic signals that causes endothelial transmigration of activated T-lymphocytes. Cleaved CD95L induces the rapid emission of pseudopods in both activated lymphocytes and fibroblastic cells and subsequently promotes cell motility through a c-yes/Orai1/Ca^2+^/PI3K signal. Recent studies have revealed that a localized Ca^2+^ rise occurs beneath the immune synapse upon the TCR engagement through the activation of the Ca^2+^ release-activated Ca^2+^ (CRAC) channel consisting of STIM1 and Orai1 [53]. STIM-1 is an ER-store Ca^2+^ sensing molecule [38],[39],[41] that links store depletion to Orai1 aggregation, which in turn leads to the selective Ca^2+^ entry. In this study, we ascertain that the CRAC channel consisting of the *pore*-forming subunit Orai1 plays a crucial role in the Ca^2+^ entry observed in the presence of cl-CD95L. In addition, this Ca^2+^ response that promotes the CD95-mediated cell motility does not display a homogeneous cytosolic distribution but instead constitutes a Ca^2+^ microdomain localized at the leading edge of the cell protrusion emitted by the migrating cells. This heterogeneous Ca^2+^ distribution has recently been described as essential in steering cell migration [33] and we speculate that the Ca^2+^ microdomain observed contiguous to the CD95-CAP may play a similar function. The spatial and temporal co-distribution of the PI3K activity (production of PIP3), actin polarization, and Ca^2+^ increase at the leading edge of the CD95-driven pseudopods suggests interconnections between these different processes. This assumption is supported by the fact that failure to induce a calcium response hinders Akt activation and, as a result, decreases cell migration. Few publications have reported molecular targets linking cytosolic Ca^2+^ increase to Akt activity; for instance, a calmodulin-dependent mechanism in glioblastoma [54] and a PKCα-dependent process in HUVEC [55] enhance Akt phosphorylation and thus activation. Nevertheless, the molecular mechanism underlying the modulation of the Akt activity following the formation of polarized Ca^2+^ microdomains remains totally unknown and would require further investigation.

Upon CD95 engagement, the proximal molecular events leading to activation of the non-canonical PI3K signal remains poorly defined. Here, we underscore that despite the fact that the CD95-mediated PI3K activation occurs through a death domain (DD)-dependent mechanism, FADD and the initiator caspases are not detected in the newly identified CD95-containing complex, suggesting either that another adaptor protein may participate in this CD95-mediated “non-orthodox” signal or that undetectable amounts of FADD remain sufficient to promote both the partition of CD95 into aggregated lipid rafts and the subsequent activation of src kinase yes through a yet unknown signaling pathway. In agreement with this latter notion, it has previously been reported that 2% of the caspase-8 activity is sufficient to redistribute CD95 into lipid raft platform through a ceramide-driven process and thus to evoke the apoptotic signal [56]. However, this assumption seems unlikely since inhibition of the caspase activity does not affect either PI3K/Akt activation or cell motility. The newly disclosed CD95-containing complex, which gathers the src kinase c-yes, has been designated MISC for *Mobility-inducing signaling complex* in comparison with the *Death-inducing signaling complex* (DISC) [57]. Recent evidence emphasizes that the tyrosine kinase c-src abrogates the caspase-8 activity through its phosphorylation on tyrosine 380, which serves as a docking site for the recruitment and the activation of p85, the regulatory sub-unit of the class IA PI3K [58]. Nevertheless, the implication of caspase-8 in the recruitment and activation of the PI3K signal remains doubtful in the context of the MISC since no caspase-8 or -10 were detected in this CD95-containing complex. In conclusion, even if the CD95-mediated c-yes activation is instrumental in eliciting PI3K/Akt signal and cell migration, the molecular ordering connecting the src kinase to the activation of PI3K/Akt remains to be clarified.

Surprisingly, redistribution of CD95 into lipid rafts has initially been described as a crucial step in the induction of the apoptotic signal [59]–[64]. It is tempting to postulate that as we previously reported [65], at least two different types of lipid rafts can be gathered around CD95, and thus, according to the composition of the recruited lipid platform, an opposite signal may be transduced in the presence of cleaved and membrane-bound CD95L. This hypothesis has still to be confirmed, and furthermore, our observations raise the question of how ligands that only diverge by their stoichiometry may account for the partition of CD95 into different types of lipid rafts.

This new observation not only is important to better appreciate the function of seric CD95L in cell biology but also offers the opportunity to gain insight into mechanisms underlying autoimmune disorders. Soluble CD95L was significantly increased in SLE patients as compared to healthy individuals, and furthermore, the concentration of CD95L was correlated with the activity of the autoimmune disease. We were concerned that the mix of cytokines present in the serum of SLE patients may affect the effect of cl-CD95L. However, we showed that in contrast to sera from healthy donors, soluble CD95L present in SLE patients efficiently achieved clustering of CD95 at the leading edge of the emitted pseudopods, which promoted both adhesion and transmigration across endothelial cells of the activated T-lymphocytes. Overall, the naturally processed CD95L cytokine evokes lymphocyte motility, which may account for the accumulation of cytotoxic T-cells in inflamed areas, causing tissue damages associated with chronic inflammatory disorders. Indeed, cell migration contributes to leukocyte extravasation and metastasis transition, among others, and these cellular mechanisms participate in the chronicity of inflammatory disorders and subsequent malignancy occurrence. Identification of cleaved-CD95L as a cytokine underlying these cellular processes may hold promises of new therapeutical approaches to prevent both tissue infiltration and damages. As a consequence and counter-intuitively, these findings point out that soluble CD95L may accelerate tumorigenesis through the activation of pro-survival, pro-proliferative signals and besides by promoting cell migration.

In agreement with two recent publications [66],[67], the concept of CD95-mediated apoptosis contributing to elimination of unwanted and damaged cells can be revisited. Even if the so-called “death receptor” CD95 is not only dedicated to induce cell death, the molecular mechanisms finely tuning the switch from apoptotic to non-apoptosis signals or vice-versa remain unknown. Herein, we show that a crucial factor monitoring the CD95 signaling pathway is its ligand itself. Indeed, the post-translational modification consisting in the cleavage by metalloprotease of the membrane-bound CD95L creates a new ligand displaying totally different functions. Whereas the membrane-bound CD95L helps to contract the immune response and maintains peripheral tolerance, its metalloprotease-cleaved counterparts released in blood circulation induce non-apoptotic signals promoting cell migration, which plays a pivotal function in inflammation and tumorigenesis.

## Materials and Methods

### Ethics Statement

All clinical investigations have been conducted according to the principles expressed in the Declaration of Helsinki. Blood was sampled from patients diagnosed with SLE after written consent was obtained from each individual. This study was approved by the Institutional Review Board at the Centre Hospitalier Universitaire de Bordeaux.

### Patients

All SLE patients fulfilled four or more of the 1982 revised ACR criteria for the disease. C3 and C4 complement components were measured by nephelometry using commercially available kits (Siemens Healthcare, Saint Denis, France).

### Antibodies, Plasmids, and Other Reagents

BTP2, PP2, LY294002, Wortmannin, BAPTA-AM ([1,2-bis-(o-Aminophenoxy)ethane-N,N,N′,N′-tetraacetic Acid Tetra-(acetoxymethyl) Ester]), and 2-APB (2-Aminoethoxydiphenyl borate) were purchased from Calbiochem (Merck Chemicals Ltd., Nottingham, UK). PHA, DAPI, Fura-2AM, FITC-conjugated cholera toxin B subunit, and DiOC6 were purchased from Sigma-Aldrich (L'Isle-d'Abeau-Chesnes, France). Anti-Akt and anti-phospho-Akt antisera were from Cell Signaling Technology, Inc. (Boston, MA, USA). The homemade soluble CD95L (gp190-CD95L) was generated in the laboratory [27]. Anti-caspase-8 (C15) was purchased from Axxora (Coger S.A., Paris, France). Anti-human CD95 mAb (DX2) was from BD Biosciences (Le Pont de Claix, France). Anti-c-Yes, Anti-Akt, anti-Akt-phosphoS473-, anti-PLCγ1, and anti-phospho-PLCγ1 antibodies were from Cell Signaling Technology (Boston, MA, USA). The anti-human Orai1 was from Abcam (Paris, France). Plasmid encoding the EGFP-Lifeact was a kind gift from Dr. R. Wedlich-Söldner (Max Planck Institute of Biochemistry, Martinsried, Germany) [32]. Lck-GFP- and PH_Akt_-GFP-containing vectors were provided by Dr. Rodgers (Oklahoma Medical Research Foundation, Oklahoma City, USA) [51] and Dr. T. Balla (National Institutes of Health, Bethesda, USA) [68], respectively. The pCR3-FasL-S126E/L127E came from Dr. P. Schneider (University of Lausanne, Epalinges, Switzerland) [20]. The pEGFP-C2 plasmids encoding GFP-Orai1 and GFP-Orai1_E106A_ were kindly provided by Dr. M. Cahalan (University of California, Irvine, CA, USA).

### Cell Lines and Peripheral Blood Lymphocytes

The human leukemic T-cell line Jurkat and the lymphoma T-cell line H9 were maintained in RPMI supplemented with 8% v/v heat-inactivated FCS and 2 mM L-glutamine at 37°C in a 5% CO_2_ incubator. 293 cells and the hamster fibroblastic cell line PS120 were cultured in DMEM supplemented with 8% v/v heat-inactivated FCS and 2 mM L-glutamine at 37°C in a 5% CO_2_ incubator. PBMCs (peripheral blood mononuclear cells) from healthy donors were isolated by Ficoll centrifugation and washed twice in PBS. Monocytes were removed by a 2 h adherence step and the naive PBLs (peripheral blood lymphocytes) were incubated overnight in RPMI supplemented with 1 µg/ml of PHA. Cells were washed extensively and incubated in the culture medium supplemented with 100 units/ml of recombinant IL-2 (PeproTech Inc., Rocky Hill, NJ, USA) for 6 d. Human umbilical vein endothelial cells (HUVEC) [69] were grown in human endothelial serum-free medium (Invitrogen, Cergy Pontoise, France) supplemented with 20% FCS, 20 ng/ml basics FGF, 10 ng/ml EGF (Invitrogen), and 1 µg/ml heparin (Sigma-Aldrich).

In order to select stable clones, Jurkat cells were transfected as previously mentioned and then placed in a medium supplemented with 1.8 mg/ml of neomycin. GFP-, GFP-Orai1_WT_, and GFP-Orai1_E106A_-expressing cells were cloned by limiting dilutions and next selected based on their expression of GFP using flow cytometry. Silencing experiments were performed by lentiviral transduction of H9 T-cells or PS120^CD95^ using validated shRNAmir-pGIPZ vectors for c-yes (RHS4430-98843955, -99161516, -98843955), Orai1 (RHS4430-98715881, -101067842), or a nontargeting shRNAmir-pGIPZ vector as a negative control (Open Biosystems, USA). To improve the percentage of transduced T-cells, living cells were harvested 72 h after transduction and green cells (pGIPZ encodes GFP) were sorted by flow cytometry using FACSAria (BD Bioscience).

### Cleaved-CD95L Production

293 cells maintained in a 1% FCS-containing medium were transfected using Calcium/Phosphate precipitation method with 3 µg of empty plasmid or wild type CD95L-containing vector. Media containing cleaved CD95L and exosome-bound full-length CD95L were harvested 5 d after transfection. Dead cells and debris were eliminated through two steps of centrifugation (4,500 rpm/15 min), and then exosomes were pelleted via an ultracentrifugation step (100,000 g/2 h). Finally, debris- and exosome-free supernatants were concentrated (10 kDa cut-off centricon) and dialyzed against PBS.

### CD95L ELISA

Anti-CD95L ELISA (Diaclone, Besançon, France) was performed to accurately quantify the cleaved-CD95L present in sera following the manufacturer's recommendations.

### Cell Death Assay

Cell viability was assessed using MTT assay, exactly as previously described [63]. In brief, 4.10^4^ cells were cultured for 24 h in flat-bottom, 96-well plates with the indicated concentrations of the apoptosis inducer in a final volume of 100 µl. 15 µl of MTT (5 mg/ml in PBS) solution were added, and after 4 h of incubation at 37°C, the absorbance was measured at 570 nm wavelength using the Titertek Labsystems Multiskan reader (Turku, Finland).

### Flow Cytometry Analysis

All steps were performed at 4°C. Cells were washed in PBS/1% (w/v) BSA, washed with PBS, and then stained with anti-CD95 mAb (clone DX2) for 30 min at 4°C. Cells were incubated for 30 min with a FITC-conjugated seconfodary antibody and immediately analyzed using FACScalibur (BD Bioscience).

### Immunoblot Analysis

Cells were lyzed for 30 min at 4°C in lysis buffer (25 mM HEPES pH 7.4, 1% v/v Triton X-100, 150 mM NaCl, 2 mM EGTA supplemented with a mix of protease inhibitors; Sigma-Aldrich). Protein concentration was determined by the bicinchoninic acid method (PIERCE, Rockford, IL, USA) according to the manufacturer's protocol. Proteins were separated on a 12% SDS-PAGE and transferred to a nitrocellulose membrane (GE Healthcare, Buckinghamshire, UK). The membrane was blocked 15 min with TBST (50 mM Tris, 160 mM NaCl, 0.05% v/v Tween 20, pH 7.8) containing 5% w/v dried skimmed milk (TBSTM). Primary antibody was incubated overnight at 4°C in TBSTM. The membrane was intensively washed (TBST), and then the peroxydase-labeled anti-rabbit or anti-mouse (SouthernBiotech, Birmingham, Alabama, USA) was added for 45 min. The proteins were visualized with the enhanced chemiluminescence substrate kit (ECL, GE Healthcare).

### DISC and MISC immunoprecipitations

The T-cell line H9 and activated PBLs (20.10^6^ cells per condition) were incubated with 1 µg/ml of APO1-3 or 100 ng/ml cl-CD95L for 15 min at 4°C (0 min) or at 37°C (15 min). Cells were then lysed and CD95 was immunoprecipitated using protein A-sepharose beads (Sigma-Aldrich). To immunoprecipitate the cl-CD95L-induced CD95-containing complex, 1 µg of Apo1-3 was added in the cell lysate and CD95 was immunoprecipitated as previously mentioned. After extensive washing, the immune complex was resolved using a 12% SDS-PAGE.

### Immunofluorescence Imaging

Cells were left to adhere 5 min at room temperature to poly-_L_-Lysine-coated slides and treated with Ig-CD95L or cl-CD95L for indicated times at 37°C. After extensive washing, cells were fixed in PBS containing 4% w/v paraformaldehyde for 15 min. The aldehyde groups were quenched for 10 min using a solution of PBS supplemented with 5% FCS. Cells were incubated with 1 µg/ml of the anti-CD95 mAb (APO1-3) for 30 min at 4°C. Finally, CD95 was revealed using either the Alexa488-conjugated (green) or the Alexa555-conjugated (red) goat anti-mouse antibody (Molecular Probes, Cergy Pontoise, France) in PBS/1% w/v BSA for 30 min at 4°C. Lipid rafts were tagged by FITC-labeled cholera toxin B (CTB). For Orai1 staining, cells were incubated with APO1-3 and 1 µg/ml of the anti-Orai1 mAb in PBS/1% w/v BSA for 60 min at room temperature. CD95 was revealed using secondary Alexa594-coupled goat anti-mouse mAb and Orai1 was observed using Alexa488-conjugated donkey anti-rabbit mAb (Invitrogen, Carlsbad, CA, USA) for 60 min at room temperature. Slides were washed with PBS, dried, and mounted with Fluorescent Mounting Media (Dako, Carpinteria, CA, USA). Images were acquired with a confocal microscope TSC SP5 (Leica, Wetzlar, Germany) with a 63× objective.

### In Vitro Motility Assays

#### Boyden chamber assay

Boyden chambers (Millipore, Molsheim, France) containing 8 µm pore size positron emission tomograph membranes were cultured in 24-well plates. After hydration of the membranes, 10^5^ cells were added to the top chamber in a low serum (1%)-containing medium. The bottom chamber was filled with low serum (1%)-containing medium in the presence or absence of 100 ng/ml of cl-CD95L. Cells were cultured for 24 h at 37°C in a 5% CO2, humidified incubator. To quantify invasion, cells were removed from the top side of the membrane mechanically using a cotton-tipped swab, and invading cells from the reverse side were fixed with methanol and stained with Giemsa. For each experiment, five representative pictures were taken for each insert, then cells were lyzed and absorbance at 560 nm correlated to the amount of Giemsa stain was measured. For experiments in which cells were incubated with inhibitors, cells were pre-incubated with inhibitors for 30 min prior to addition in the top chamber.

#### Wound healing assay

Cells (10^6^) were seeded in a 6-well plate and cultured until confluence. Next, a straight scratch was performed in the monolayer of cells using a pipette tip. Imaging of the two wound edges were performed using the 10× objective. For each condition, images were acquired at five different positions along the scratch and a representative field was depicted.

### Adhesion and Transendothelial Migration of Activated T-Lymphocytes

#### Transmigration

Assays for endothelial transmigration of activated PBLs were performed in Boyden chambers with polycarbonate filters of 8 µm pore size (Costar). Activated T-lymphocytes (2×10^6^) were placed in the upper chamber on a confluent monolayer of HUVEC. 1 ml of serum from either healthy or SLE patients was added in the lower reservoir, and after 24 h, transmigrated cells were counted in the lower reservoir by flow cytometry using a standard of 2.5×10^4^ fluorescent beads (Flow-count, Beckman Coulter).

#### Adherence

Determination of T-lymphocyte adherence to endothelial monolayers was performed with a fluorescence multi-well plate reader (Cytifluor, Framingham, MA, USA). Briefly, 2×10^6^ activated T-lymphocytes were added to the upper chamber and 1 ml of different plasma samples in the lower reservoir. Cells were allowed to adhere for 1 h at 37°C and 5% CO_2_ atmosphere, and after extensive washing with HBSS, cell-associated fluorescence was measured. Results are depicted as relative mean adherence corresponding to the ratio of fluorescence values before and after washing.

### Video Imaging of Calcium Response in Living Cells

T-cells were loaded with Fura2-AM (1 µM) at resting temperature for 30 min in Hank's Balanced Salt Solution (HBSS). After washing with HBSS, the cells were incubated for 15 min in the absence of Fura2-AM to complete de-esterification of the dye. Cells were placed in a thermostated chamber (37°C) of an inverted epifluorescence microscope (Olympus IX70) equipped with a ×40, UApo/340–1.15 W water-immersion objective (Olympus), and fluorescence micrograph images were captured at 510 nm and at 12-bit resolution by a fast-scan camera (CoolSNAP fx Monochrome, Photometrics). To minimize UV light exposure, 4×4 binning function was used. Fura2-AM was alternately excited at 340 and 380 nm, and ratios of the resulting images (excitations at 340 and 380 nm and emission filter at 520 nm) were produced at constant intervals (5 s or 10 s according to the stimulus). Fura-2 ratio (F_ratio_ 340/380) images were displayed and the F_ratio_ values from the regions of interest (ROIs) drawn on individual cells were monitored during the experiments and analyzed later offline with Universal Imaging software, including Metafluor and Metamorph. Each experiment was independently repeated 3 times, and for each experimental condition, we displayed an average of more than 20 single-cell traces. Fluorescent images were pseudocolored using the IMD display mode in MetaFluor and assembled without further manipulation in Photoshop (Adobe). Raw data were acquired with MetaFluor and graphed in Origin (OriginLab). [Ca^2+^]_i_ was calculated using the following equation: [Ca^2+^]_i_ = K_d_(R−R_min_)/(R−R_max_)×Sf2/Sf1, where K_d_ is the Fura2-AM dissociation constant at the two excitation wavelengths (F_340_/F_380_); R_min_ is the fluorescence ratio in the presence of minimal calcium, obtained by chelating Ca^2+^ with 10 mM EGTA; R_max_ is the fluorescence ratio in the presence of excess calcium, obtained by treating cells with 1 µM ionomycin; Sf2 is the fluorescence of the Ca^2+^-free form; and Sf1 is the fluorescence of the Ca^2+^-bound form of Fura2-AM at excitation wavelengths of 380 and 340 nm, respectively.

In some experiments cells were placed in a Ca^2+^-free medium consisting of the HBSS described above in which CaCl_2_ was omitted and 100 µM EGTA was added in order to chelate residual Ca^2+^ ions. This medium was added to the cells just before recording to avoid leak of the intracellular calcium stores.

In experiments combining CD95 immuno-staining and Ca^2+^ imaging, cells were incubated or not with 100 ng/ml of cleaved-CD95L at 4°C and then washed. To visualize the plasma membrane distribution of CD95, the cells were incubated at 4°C with the non-agonistic anti-CD95 mAb DX2 (1 µg/ml) associated with an Alexa555-coupled goat anti-mouse mAb (GAM). Finally, the CD95-stained cells were mixed with 1 µM fura-2AM and cl-CD95L for 30 min at RT in HBSS solution. CD95 immuno-staining was imaged at the beginning and at the end of the experiment. Each experiment was independently repeated 3 times, and for each experimental condition, we displayed an average of more than 15 single-cell traces.

## Supporting Information

Figure S1 The naturally processed CD95L fails to trigger caspase-8 activation and cell death. (A) The human embryonic kidney epithelial cell line 293T (HEK) was transfected with wild type CD95L or the mutated CD95L^S126E/L127E^. Cells were lyzed (Cell) and supernatants were harvested (Sn) 5 d after transfection. Dead cells were eliminated from the supernatant by centrifugation (2×4,000 rpm for 15 min), and then exosomes were pelleted using ultracentrifugation (100,000 g/2 h) and lyzed (Exo.). White arrowhead indicates cleaved-CD95L. Black arrowhead depicts full-length CD95L and the star indicates an unknown processed CD95L product. (B) The molecular size of cleaved CD95L was analyzed using size exclusion S-200-HR Sephacryl columns (Amersham Pharmacia, Orsay, France). Fractions were harvested and cl-CD95L was dosed by ELISA. (C) Jurkat and H9 T-cells were treated with 100 ng/ml of the home-made Ig-CD95L (dodecameric) or the naturally processed CD95L (trimeric), and activation of caspase-8 was assessed by following the cleavage of the protease using immunoblot (fragments p41/43 and p18). For each cell, long exposures of the film are depicted to detect the p18 cleaved fragment. Stars indicate irrelevant bands. (D) Indicated cells were incubated for 24 h with an engineered dodecameric Ig-CD95L or the naturally processed homotrimeric CD95L and cell death was assessed by MTT assay.
(TIF)

Figure S2 Cleaved CD95L induces formation of CD95-Cap at the extremity of the emitted pseudopod. (A) The leukemic T-cell line H9 was incubated for indicated times with 100 ng/ml of cleaved CD95L, and after extensive washing, cells were fixed. CD95 was stained using an anti-CD95 mAb (APO1-3) and revealed using the secondary Alexa488-conjugated goat anti-mouse antibody (Invitrogen, Carlsbad, CA, USA). Number of cells harboring a CD95-Cap was counted (at least 300 cells counted for each condition). (B) The leukemic T-cell lines CEM and H9 were incubated for 30 min in the presence or absence of 100 ng/ml of cleaved CD95L, and the plasma membrane distribution of CD95 was then analyzed as described in (A). The quantity of cells harboring a CD95-Cap was assessed by counting (300 cells/condition). Values represent means and SD of three independently performed experiments. **p<0.01 and ***p<0.001 as calculated using non-parametric and two-tailed Mann-Whitney test. (C) Leukemic T-cells H9 and CEM were untreated (control) or treated for 30 min with cleaved CD95L (100 ng/ml), and after extensive washing, cells were fixed. CD95 was stained using an anti-CD95 mAb (APO1-3) and revealed using a secondary Alexa488-conjugated goat anti-mouse antibody. Nuclei were stained with DAPI (blue). Slides were washed with PBS, dried, and mounted with Fluoromount (Cliniscience SAS, Montrouge, France). Red arrows depict emitted pseudopods upon CD95 engagement. Images were acquired with a confocal microscope TSC SP5 (Leica, Wetzlar, Germany) with a ApoPLAN 63× objective.
(TIF)

Figure S3 CD95 and truncated CD95(1-210)-expressing PS120 clones. (A) *Upper panel*: analysis of the CD95 expression at the surface of the indicated PS120 clones stably expressing either the CD95 wild type or its death domain truncated counterpart (Δ1-210). Cells were stained with an anti-CD95 mAb (clone DX2), washed, and a PE-coupled goat anti-mouse secondary antibody was used to reveal plasma membrane CD95 by flow cytometry. *Lower panel*: for each staining, the mean of the fluorescence intensity (MFI), which is correlated to the amount of plasma membrane CD95, was depicted. (B) Indicated PS120 clones expressing either empty vector (PS120^control^), truncated (PS120^CD95(Δ1-210)^), or wild type CD95 (PS120^CD95^) were incubated with the cytotoxic Ig-CD95L or the cleaved CD95L for 24 h and cell death was quantified using viability assay MTT.
(TIF)

Figure S4 Cleaved CD95L triggers a pseudopod-localized Ca^2+^ rise. *Video imaging on living cells*: Before application of cl-CD95L, activated T-lymphocytes were loaded with the Ca^2+^ indicator Fura-2AM along with EGTA-AM (1 µM, 30 min, room temperature), a slow high-affinity Ca^2+^ buffer. Under these conditions, Ca^2+^ entering the cell would bind rapidly to the Ca^2+^ probe, producing a fluorescent signal, and then be captured by EGTA. Activated T-lymphocytes were bathed in an external medium containing 2 mM Ca^2+^ and stimulated with 100 ng/ml of cl-CD95L just after the capture of the ratio images (0 s). Pictures were recorded at the indicated times following the addition of cl-CD95L to assess the formation of localized Ca^2+^ influx. Emitted pseudopods were depicted using a white square, and cell migration is indicated by a white arrow. Grey levels were translated to false colors according to a scale shown on the right.
(TIF)

Figure S5 The cl-CD95L-mediated calcium response occurs through a PLCγ1/IP3-R-dependent process. (A) The T-cell line Jurkat was incubated for the indicated times with 100 ng/ml of cl-CD95L, and then cells were lyzed. 100 µg of protein was loaded per lane and resolved in a 10% SDS-PAGE. Immunoblots were performed using indicated mAbs (Cell Signaling technology, Ozyme, Saint Quentin, France). (B) *Left panel*: The PLC-γ1-deficient T-cell Jurkat and its PLC-γ1-reconstituted counterpart were loaded with the calcium probe Fura2-AM (1 µM, 30 min at RT) and then stimulated with 100 ng/ml of cl-CD95L. Ratio images were taken every 10 s. Regions of Interest (ROIs) outlining individual cells were defined in three independent experiments, and the mean ratio in each ROI was plotted versus time. Ratio values were converted to [Ca^2+^]i values using a calibration curve (see Materials and Methods section). *Right panel*: Statistical analyses of the AUC values for the indicated T-cells. *** *p* values≤0.001 using non-parametric two-tailed Mann-Whitney test. (C, D) *Left panels*: Jurkat (C) and H9 (D) T-cells were loaded as previously mentioned. The IP3-R inhibitor, 2-APB (20 µM), was applied during the recording as indicated by the unfilled rectangle. Then cells were stimulated with 100 ng/ml of cl-CD95L (black arrow) and the intracellular Ca^2+^ was recorded every 10 s. Regions of Interest (ROIs) outlining individual cells were defined in three independently performed experiments and the mean ratio in each ROI was plotted versus time. Ratio values were converted to [Ca^2+^]i values using a calibration curve (see Materials and Methods section). Values depict the mean ± SD of the area under the curve (AUC) calculated for 1,000 s. *Right panels*: statistical analyses of the AUC values. *** *p* value≤0.001 using non-parametric two-tailed Mann-Whitney test.
(TIF)

Figure S6 Extracellular Ca^2+^ plays a crucial role in the cl-CD95L-mediated Ca^2+^ rise. (A) Indicated T-cells were loaded with 1 µM of the calcium probe Fura-2AM for 30 min at RT. Cells were bathed at 37°C in a medium containing 2 mM Ca^2+^ (2 mM [Ca^2+^]_e_) or a Ca^2+^-free medium (0 mM [Ca^2+^]_e_) and then untreated (vehicle) or treated (cl-CD95L) with 100 ng/ml of cl-CD95L. Values were recorded every 10 s. Regions of Interest (ROIs) outlining individual cells were defined in three independently performed experiments and the mean ratio in each ROI was plotted versus time. Ratio values were converted to [Ca^2+^]i values using a calibration curve (see Materials and Methods section). For each treatment, the mean ± SD of the area under the curve (AUC) measured for 1,000 s in individual cells is depicted. Basal and maximal levels of [Ca^2+^]_i_ are indicated by red and blue dotted lines, respectively. (B) Statistical analyses of the AUC values for indicated cells incubated in the presence of cl-CD95L in a regular (2 mM) or a Ca^2+^-free medium (0 mM). Using non-parametric two-tailed Mann-Whitney test, *** indicates a *p* value≤0.001. (C) PBLs were untreated (upper panel) or treated (lower panel) with the SOC channel inhibitor BTP-2 (500 nM) and then 100 ng/ml of cl-CD95L was added. For each condition, the mean ± SD of the area under the curve (AUC) measured for 1,000 s is depicted. Basal and maximal levels of [Ca^2+^]_i_ are indicated by red and blue dotted lines, respectively. Note that individual calcium oscillations and/or synchronous calcium oscillations (upper panel) lead to apparent calcium oscillations during the plateau phase.
(TIF)

Figure S7 BAPTA-AM abrogates the cl-CD95L-mediated Ca^2+^ response. (A) H9 T-cells were loaded with 1 µM of the calcium probe Fura-2AM for 30 min at RT. Cells were pre-incubated or not with BAPTA-AM (5 µM) and then stimulated with 100 ng/ml of cl-CD95L. Values were recorded every 10 s. Regions of Interest (ROIs) outlining individual cells were defined in three independently performed experiments, and the mean ratio in each ROI was plotted versus time. Ratio values were converted to [Ca^2+^]i values using a calibration curve (see Materials and Methods section). For each treatment, the mean ± SD of the area under the curve (AUC) measured for 1,000 s is depicted. (B) Statistical analyses of the AUC values for H9 T-cells treated or untreated with 5 µM of BAPTA-AM and stimulated with cl-CD95L. Using non-parametric two-tailed Mann-Whitney test, *** indicates a *p* value≤0.001.
(TIF)

Figure S8 Identification of the p110 isoform eliciting the cl-CD95L-mediated cell motility. (A) The table depicts IC50 (nM) of the different isoform-selective inhibitors on the class I PI3Ks. PI3K-α Inh-IV (3-(4-Morpholinothieno[3,2-d]pyrimidin-2-yl)phenol); PI3K-β Inh-VI/TGX-221 ((±)-7-Methyl-2-(morpholin-4-yl)-9-(1-phenylaminoethyl)-pyrido[1,2-a]-pyrimidin-4-one); PI3K-γ Inh (5-Quinoxalin-6-ylmethylene-thiazolidine-2,4-dione); PI3K-δ Inh-X, IC87114; and Wortmannin and LY294002 were tested to ascertain the isoform involved in the cl-CD95L-mediated motility. All the inhibitors came from Calbiochem (Merck Chemicals Ltd., Nottingham, UK). Note: Data were compiled from *Billottet et al., 2009, Cancer Research, 69(3):1027-36/Hayakawa et al., 2006, Bioorganic & Medicinal Chemistry, 14(20):6847-58*. (B, C, D, and E) For each inhibitor, the concentrations used in the different *in cellulo* assays were defined according to the respective isoform-selective IC50 indicated in bold in (A). The T-cell line H9 (B) and the fibroblastic cell line PS120^CD95^ (C) were pre-incubated with the indicated concentrations of the pan-PI3K inhibitors wortmannin and LY294002 or the isoform-selective inhibitors PI3K-α Inh-IV (α); PI3K-β inh-VI (β); PI3K-γ Inh (γ); and PI3K-δ Inh-X (δ) for 60 min and then untreated (U) or treated for 30 min with 100 ng/ml of cl-CD95L. Cells were lyzed and 100 µg of protein was loaded for each lane. Indicated immunoblots were performed. Bands were scanned and a densitometry analysis was performed using ImageJ. Values below the immunoblots indicate the percentage of phospho-Akt inhibition reached with each selective inhibitor. Data are representative of three independently performed experiments. (D) H9 T-cells were pre-incubated with a concentration of the indicated PI3K inhibitors corresponding to 100-fold their predicted IC_50_ (see A) for 60 min, and then cells were incubated in the presence or absence of cl-CD95L (100 ng/ml) for 24 h in a Boyden Chamber in which the porous membrane was covered with a confluent monolayer of endothelial cells (see Materials and Methods). Then, the endothelial transmigration of the T-cells was assessed as described in Materials and Methods. (E) The fibroblastic PS120^CD95^ cell line was pre-incubated with a concentration of the indicated PI3K inhibitors corresponding to 100-fold their predicted IC_50_ (see A) for 60 min, and then cells were incubated in the presence or absence of cl-CD95L (100 ng/ml) for 24 h in a Boyden Chamber assay. To quantitatively measure cell motility, Giemsa-stained migrating cells from the lower side of the membrane were lyzed and absorbance was measured at a wavelength of 560 nm. Values represent means and SEM of three independently performed experiments.
(TIF)

Figure S9 The caspase activity does not participate in the cl-CD95L-induced cell motility. (A) *Wound healing assays*: A confluent monolayer of the indicated adherent cells was wounded with a tip. Then, cells were pre-incubated for 30 min with 40 µM of zVAD-fmk and treated or untreated for 24 h with 100 ng/ml of cl-CD95L and pictures were acquired (Bars = 50 µm). Pictures are representative of three independently performed experiments. (B) *Upper panels*: The CD95-expressing PS120 cells were pre-incubated with or without the pan-caspase inhibitor zVAD-fmk (40 µM) for 30 min and then seeded in the upper compartment of the Boyden chamber in a low FCS (1%)-containing medium. Cells were incubated for 24 h in the presence of absence of 100 ng/ml of cl-CD95L. Then the membrane was removed, the upper side containing the non-migrating cells was wiped out with cotton-tipped swabs, and migrating cells in the opposite side of the filter were fixed with methanol and stained by Giemsa (purple cells). For each experiment, five pictures of random fields were taken and a representative picture was depicted (Bars = 70 µm). *Lower panel*: To quantify cell motility, Giemsa-stained migrating cells from the lower side of the membrane were lyzed and absorbance was measured at 560 nm. Data represent means and SD of three independently performed experiments.
(TIF)

Figure S10 The src kinase c-yes is instrumental in the cl-CD95L-induced cell motility. (A) *Wound healing assays*: A confluent monolayer of PS120^CD95^ adherent cells was wounded with a tip. Cells were pre-incubated with or without 10 µM of the src inhibitor PP2 and then were treated or untreated for 24 h with 100 ng/ml of cl-CD95L. Pictures were acquired to assess the efficiency of the wound healing. Dotted red lines delineate the initial position of the wound. (B) PS120^CD95^ Cells were transduced with lentiviral particles containing a c-yes-targeting shRNAmir or a scramble shRNAmir (OpenBiosystem, USA). 48 h after transduction, the confluent monolayer of cells was wounded and cell migration was analyzed in the presence of 100 ng/ml of cl-CD95L for the indicated time. Pictures are representative of three independently performed experiments.
(TIF)

## Abbreviations

DD: death domain
DISC: death-inducing signaling complex
ER: endoplasmic reticulum
HUVEC: human umbilical vein endothelial cells
mTOR: mammalian target of rapamycin
PBLs: peripheral blood lymphocytes
PLC: phospholipase C
ROIs: regions of interest
SLE: systemic lupus erythematosus
SOC: store-operated Ca^2+^
STIM1: stromal interaction molecule 1
