# Correction: The Naturally Processed CD95L Elicits a c-Yes/Calcium/PI3K-Driven Cell Migration Pathway

**DOI:** 10.1371/journal.pbio.3000521

**Published:** 2019-10-08

**Authors:** Sébastien Tauzin, Benjamin Chaigne-Delalande, Eric Selva, Nadine Khadra, Sophie Daburon, Cécile Contin-Bordes, Patrick Blanco, Jacques Le Seyec, Thomas Ducret, Laurent Counillon, Jean-François Moreau, Paul Hofman, Pierre Vacher, Patrick Legembre

In their article, the authors represented data obtained with PS120-NHE1 cells reconstituted with WT or (1–175) CD95 constructs, and unfortunately indicated PS120 instead of PS120-NHE1 cells in the Materials and Methods section.

Therefore, the text appearing on page 11 in the Materials and Methods section which currently states, “the hamster fibroblastic cell line PS120," should instead read as follows:

"293 cells and the hamster fibroblastic cell line PS120-NHE1 designated PS120 in the manuscript were cultured in DMEM supplemented with 8% v/v heat-inactivated FCS and 2 mM L-glutamine at 37°C in a 5% CO2 Incubator.”

## References

[1] Tauzin S, Chaigne-Delalande B, Selva E, Khadra N, Daburon S, et al (2011) The Naturally Processed CD95L Elicits a c-Yes/Calcium/PI3K-Driven Cell Migration Pathway. PLoS Biol 9(6): e1001090 10.1371/journal.pbio.1001090 21713032 PMC3119658

