# Correction: The Naturally Processed CD95L Elicits a c-Yes/Calcium/PI3K-Driven Cell Migration Pathway

**DOI:** 10.1371/journal.pbio.3002027

**Published:** 2023-02-23

**Authors:** Sébastien Tauzin, Benjamin Chaigne-Delalande, Eric Selva, Nadine Khadra, Sophie Daburon, Cécile Contin-Bordes, Patrick Blanco, Jacques Le Seyec, Thomas Ducret, Laurent Counillon, Jean-François Moreau, Paul Hofman, Pierre Vacher, Patrick Legembre

The following changes have been made to correct errors in this article and Correction [1, 2]. Additionally, reuse of data from [1] in a later article [3] is acknowledged in the corrected figure legends.

Figures and legends:

- The following figures have been updated to correct inconsistencies in the labeling: Figs 3, 4, 5, 6, S2, S3, S6, S9 and S10.
- The legends for Figs 1–6 and S1-S10 have been updated to describe the control conditions, clarify the meaning of labels, state that all PS120 cells are transfected with wild-type NHE1, and acknowledge the reuse of data in figures within this article [1] and in a later article from the same groups [3].
- The Untreated zVAD panel in the updated S9B Fig is a replacement, as the original panel was an accidental duplication of a panel in Fig 5D.
- A pair of fluorescence and brightfield panels for H9 Control were removed from S2C Fig because they did not present the same field of view.
- Images in the following figures have been replaced with unmodified versions, with no changes to contrast or brightness: Figs 4A (PS120^CD95(Δ1–210)^ panels), S9A and S10.
- Underlying image data for the western blot experiments in the following figures are provided in S1 File: Figs 2, 4, 5, S1, S5, and S8. The underlying blot for H9 cells in S1C Fig is no longer available.

The Materials and Methods section is supplemented with the following information provided in S2 File:

- Protocol for the generation of control and CD95-expressing PS120-NHE1 cells.
- Protocol for the transfection of Lifeact-GFP, PHAkt-GFP and GFP-Orai1.
- Protocol for the production of Ig-CD95.
- Information regarding CEM and SKW6.4 cell lines.
- Description of PLCg1-reconsistuted Jurkat cells.
- Sequences for all shRNAs, including scrambled.
- Suppliers for PI3K isoform selective inhibitors, zVAD.
- Additionally, the Flow Cytometry Analysis paragraph in [1] is corrected to state that a PE-coupled goat anti-mouse secondary antibody (RRID:AB_393768) was used.

In addition to the above changes in [1], an error in the previous Correction [2] is corrected, as “(1–175) CD95 constructs” should state “death-domain truncated CD95 (PS120^CD95(Δ1–210)^).”

The corresponding author apologizes for the errors in the published article and previous Correction.

**Fig 1:**
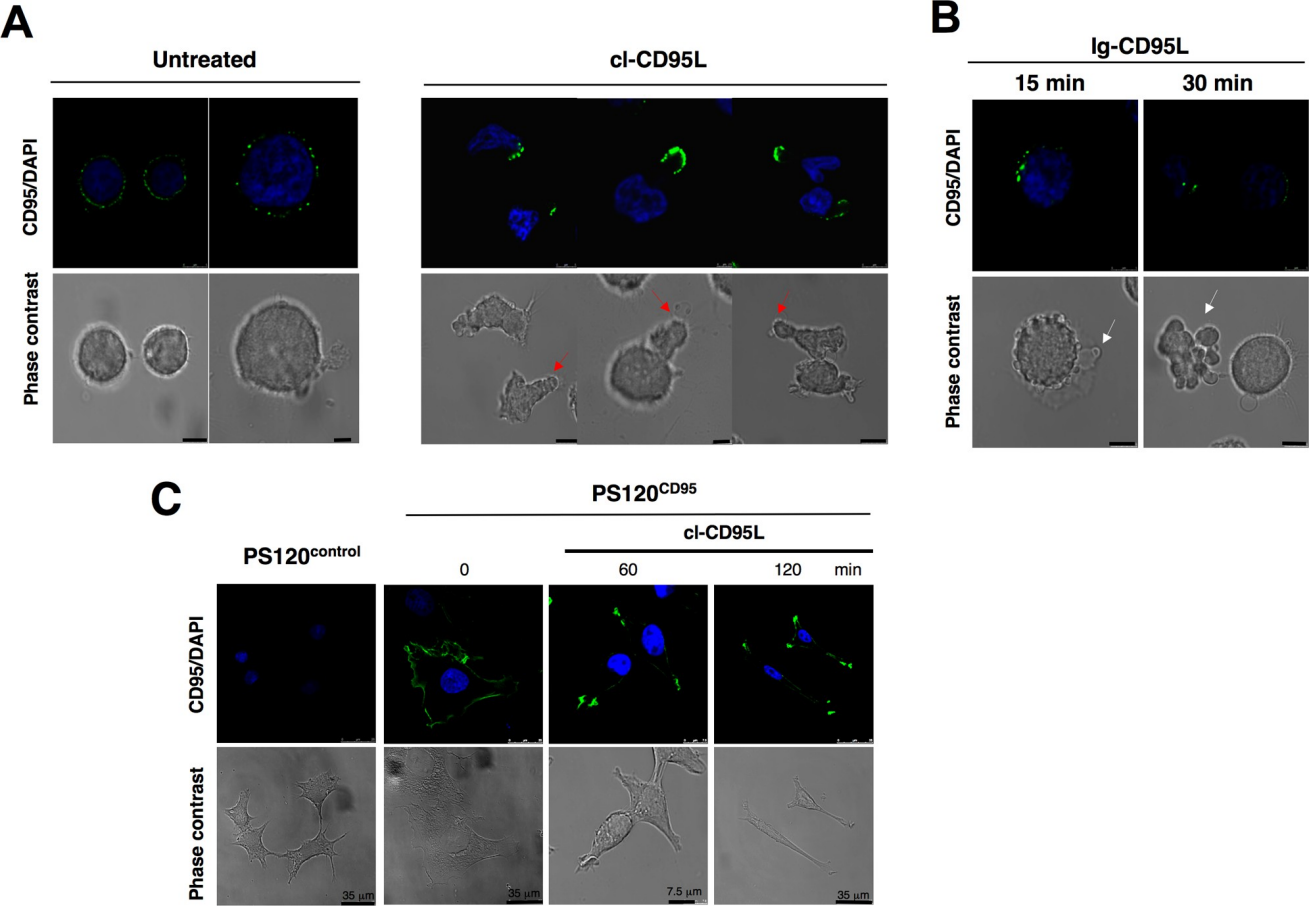
Cl-CD95L elicits CD95-CAP formation. (A) Activated (PHA/IL2)-peripheral blood T-lymphocytes (PBLs) from healthy individuals were incubated with control medium (untreated condition corresponding to the incubation with supernatant of pcDNA3.1(+)-transfected HEK cells) or 100 ng/ml of cl-CD95L for 30 min. Cell morphology was followed using phase contrast microscopy and CD95 was stained using a mouse anti-CD95 mAb (APO1-3) followed by a goat Alexa488-coupled anti-mouse IgG mAb. Red arrows depict emitted pseudopods (Bars = 5 mm). (B) PHA/IL2-activated PBLs were incubated with the cytotoxic Ig-CD95L for indicated times, and cells were analyzed as described in (A). White arrows depict blebs emitted by apoptotic cells (bars = 5 mm). (C) The fibroblastic cell line PS120 devoid of endogenous CD95 (wild type NHE1-reconstituted PS120^control^) or reconstituted with human wild type CD95 (PS120^CD95^) were untreated or treated with 100 ng/ml of cleaved CD95L for indicated times, and cell shape and CD95 distribution were analyzed. The “0” condition indicates cells analyzed before the addition of 100 ng/mL of cl-CD95L.

**Fig 2:**
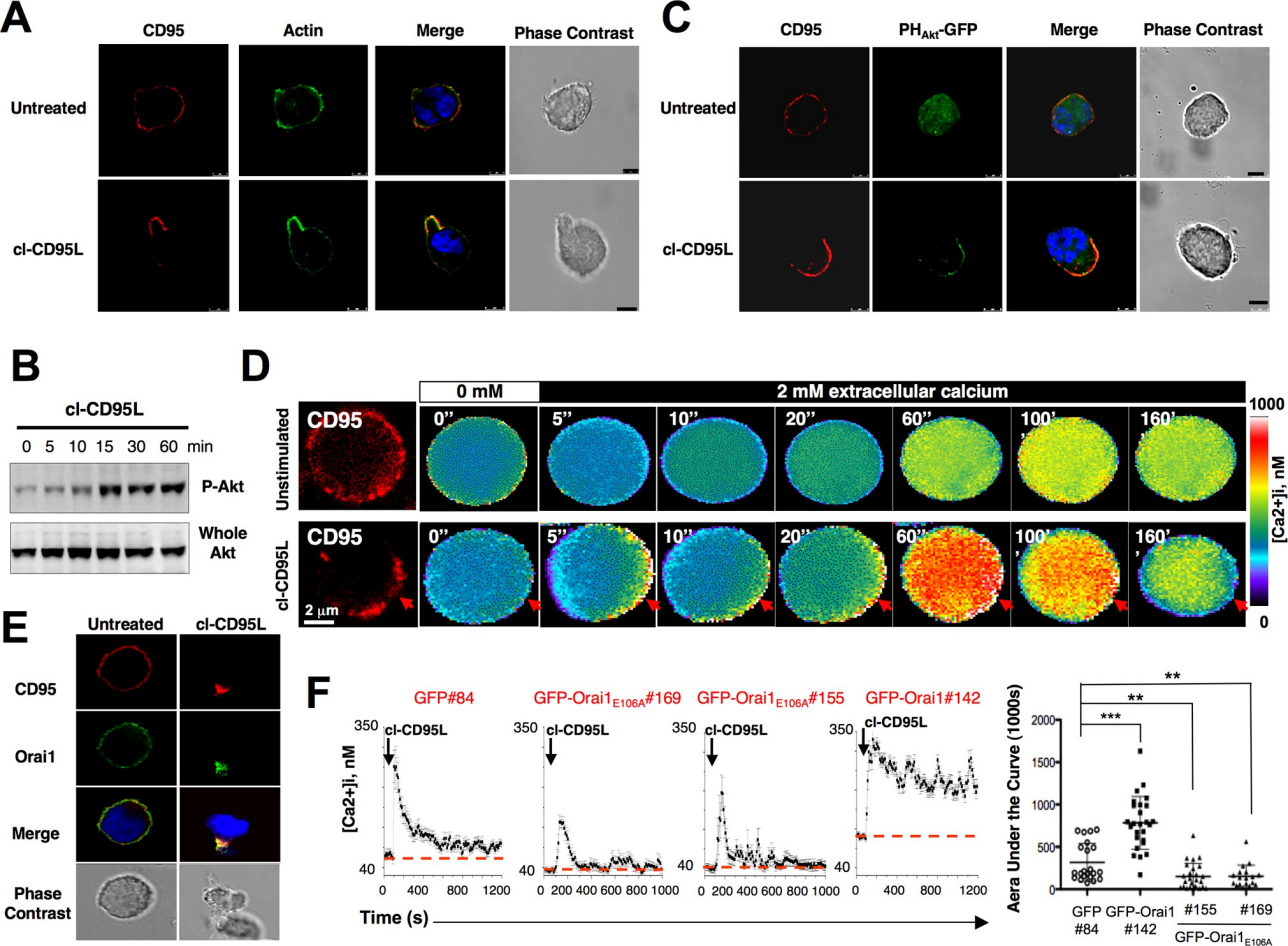
Cleaved CD95L triggers a pseudopod-localized actin/PI3K/Akt signal and a Ca^2+^ entry through activation of the CRAC channel Orai1. (A) H9 T-cells were transiently transfected with the actin marker Lifeact-GFP. Living cells were harvested (Ficoll) and treated for 30 min with 100 ng/ml of cleaved CD95L or with control medium (supernatant of pcDNA3.1(+)-transfected HEK cells—untreated). Cells were fixed, and CD95 was stained using anti-CD95 mAb (APO1-3) and revealed with the secondary Alexa555-coupled Goat anti-mouse antibody (red). Nuclei were stained using DAPI (blue). Bars = 5 mm. Images were acquired with an ApoPLAN 63× objective. (B) The leukemic T-cell line H9 was incubated with 100 ng/ml of the naturally processed CD95L for indicated times. Cells were lysed, and for each lane, 100 mg of protein were loaded. Proteins were resolved by SDS-PAGE and anti-whole Akt and anti-Akt phosphorylation (serine^473^) immunoblots were performed. Akt phosphorylation stands for its activation. (C) The pleckstrin homology (PH) domain of Akt binds PIP3 produced by PI3K activation. As a consequence, the probe PH_Akt_-GFP stains PIP3. H9 T-cells were transiently transfected with the PH_Akt_-GFP construct (green). Living cells were isolated (Ficoll) and incubated with 100 ng/ml of the naturally processed CD95L (cl-CD95L) or with control medium (supernatant from pcDNA3.1(+)-transfected HEK cells—untreated) for 30 min. Cells were fixed, and CD95 was stained as previously mentioned (red). Pictures were taken by confocal microscopy. Cell morphology was followed using differential interference contrast microscopy. Nuclei were stained with DAPI (blue). Bars = 5 mm. (D) *Calcium measurement in single cell*. Jurkat T-cells were loaded with the permeant calcium probe Fura-2AM, and in parallel, CD95 was followed using an anti-CD95 mAb and an Alexa555-coupled goat anti-mouse mAb as described in Materials and Methods. Cells were bathed in a Ca^2+^-free medium and pre-incubated (*lower panel*; cl-CD95L) or not (*upper panel*; supernatant from pcDNA3.1(+)-transfected HEK cells—untreated)) with cl-CD95L (100 ng/ml). 2 mM Ca^2+^ was added when indicated by the black-filled rectangle. Ratio images (F340 nm/F380 nm) were taken every 5 s, and the images shown correspond to the indicated period of time. Grey level intensities were translated to false colors according to the colors scale shown at the right, and [Ca^2+^]i was estimated from the ratio values and calibration experiments as described in Materials and Methods. Red arrows indicate the CD95-CAP. (E) Jurkat T-cells were treated with 100 ng/ml of cl-CD95L or with control medium (supernatant from pcDNA3.1(+)-transfected HEK cells—untreated) for 60 min. Cells were fixed and Orai1 and CD95 were stained as described in Materials and Methods. Cell morphology was analyzed using phase contrast microscopy. (F) *Left panels*: GFP-, GFP-Orai1, or GFP-Orai1_E106A_-expressing Jurkat T-cells were loaded with 1 mM of the calcium probe Fura-2AM for 30 min at RT. Cells were bathed at 37°C in a medium containing 2 mM [Ca^2+^]e and then treated with 100 ng/ml of cl-CD95L (black arrow). GFP#84 corresponded to control cells expressing GFP. GFP-Orai1_E106A_#155 and #169 corresponded to two independently isolated clones expressing the non-functional CRAC channel. GFP-Orai1#142 corresponded to Jurkat cells overexpressing human Orai1. Values were recorded every 10 s. *Right panel*: For each experiment, the area under the curve (AUC) was measured for 1,000 s, and the statistical analyses of the AUC values were performed for indicated cells using non-parametric two-tailed Mann-Whitney tests. ** and *** indicate *p* values≤0.01 and 0.001, respectively.

**Fig 3:**
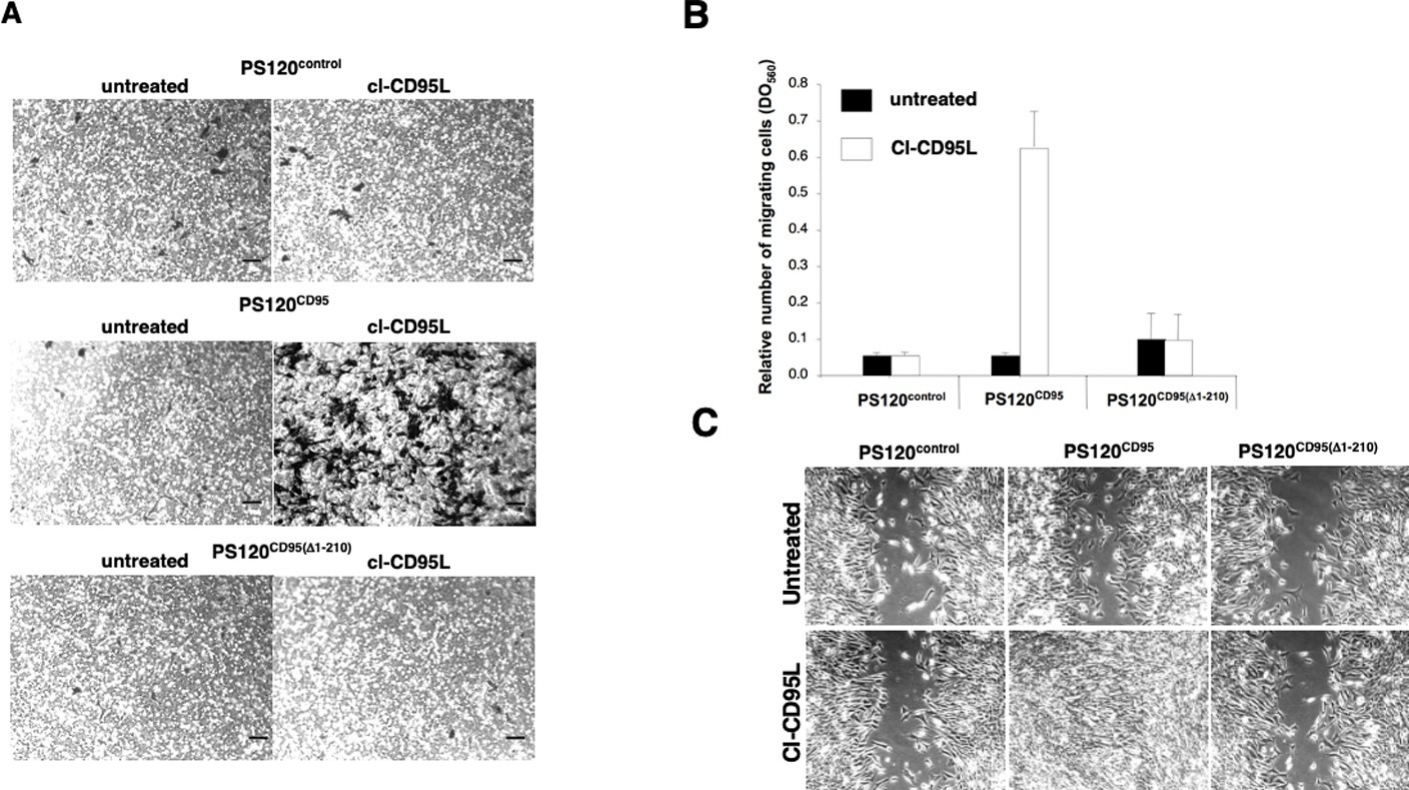
Cleaved CD95L promotes cell migration through a DD-dependent signal. (A) The CD95-deficient PS120 (wild type NHE1-reconstituted PS120^control^) and its counterparts expressing human wild type CD95 or DD-truncated CD95 were seeded in a Boyden chamber in the presence of cl-CD95L (100 ng/ml) or with control medium (supernatant from pcDNA3.1(+)-transfected HEK cells—Untreated) and incubated for 24 h. The filter was removed, the upper side containing the non-migrating cells was wiped out with cotton-tipped swabs and migrating cells in the opposite side of the filter were fixed with methanol and stained (Giemsa). For each experiment, five pictures of random fields were taken, and a representative picture was depicted (Bars  =  70 μm). (B) Cells were treated as described in (A). To quantify cell motility, Giemsa-stained migrating cells from the lower side of the membrane were lyzed and absorbance was measured at 560 nm. (C) Wound healing assay, a confluent monolayer of the indicated cells was “wounded” with a tip and then cells were incubated for 24 h in the presence of 100 ng/ml of cl-CD95L or a control medium (supernatant of pcDNA3.1(+)-transfected HEK cell) (untreated) and pictures were acquired (Bars  =  50 μm). Pictures are representative of 5 independently performed experiments.

**Fig 4:**
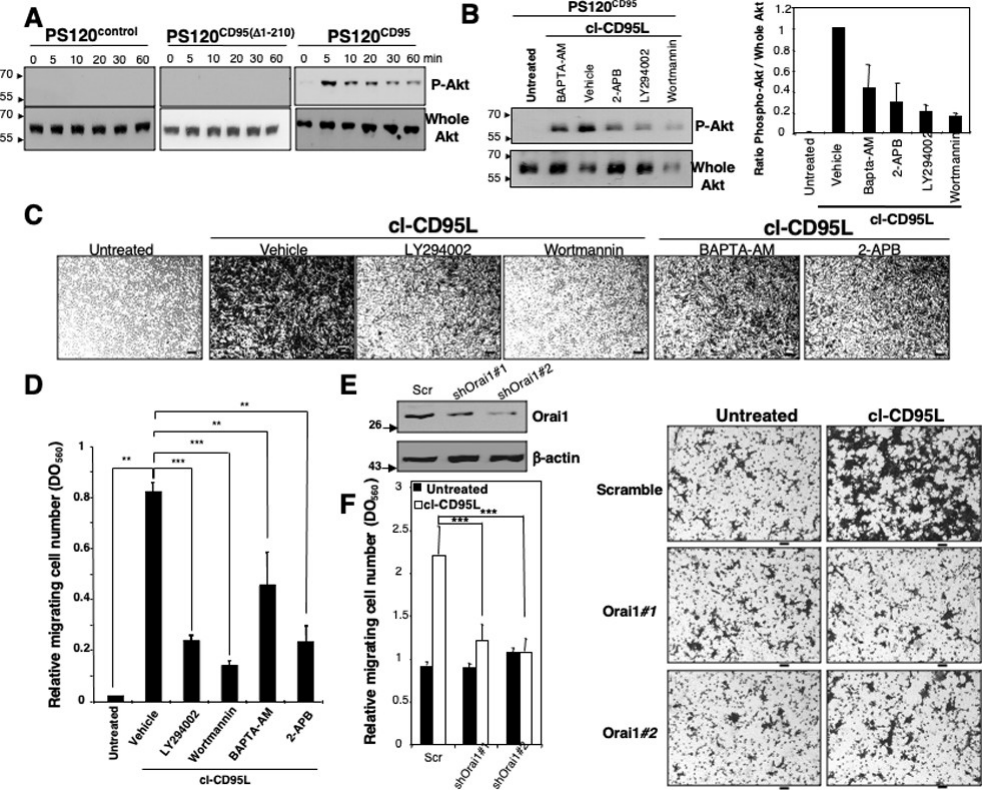
cl-CD95L promotes a cell migration through a Ca^2+^/PI3K/Akt signal. (A) The CD95-deficient fibroblastic cell line PS120 transfected with empty vector (wild type NHE1-reconstituted PS120^control^) or reconstituted with either human wild type CD95 (PS120^CD95^) or a death domain-truncated CD95 (PS120^CD95(Δ1–210)^) was incubated with 100 ng/ml of cleaved CD95L for indicated times. Cells were harvested, lyzed, and 100 μg of protein was loaded per lane. Proteins were resolved in a SDS-PAGE and immunoblots were performed. Phosphorylation of the serine^473^ on Akt indicates activation of the kinase. Whole Akt is added as loading control. The PS120 immunoblots shown in (A) have been partly reused in [3]. (B) *Left panels*: PS120^CD95^ cells were pre-incubated with non-cytotoxic and non-cytostatic concentration of PI3K inhibitors (2.5 μM of wortmannin and 5 μM of LY294002), the cell permeant chelator of calcium BAPTA-AM (5 μM), the inhibitor of IP3-R and SOC channels 2-APB (20 μM) or DMSO (vehicle). Cells were then untreated (control medium from pcDNA3.1(+)-transfected HEK cells) or treated for 5 min with 100 ng/ml of cl-CD95L and lysed, 50 μg/ml of protein was loaded per lane and indicated immunoblots were performed. *Right panel*: the densitometry analyses of the immunoblot bands were performed using ImageJ software and the histograms depict Phospho-Akt/whole Akt ratios. (C) Cell migration of PS120^CD95^ was assessed using the Boyden chamber assay. Cells were pre-incubated with non-cytotoxic and non-cytostatic concentrations of PI3K inhibitors (2.5 μM of Wortmannin and 5 μM of LY294002), the cell permeant chelator of calcium BAPTA-AM (5 μM), the inhibitor of SOC channels 2-APB (20 μM) or DMSO (vehicle) for 30 min and then stimulated with 100 ng/ml of cl-CD95L or a control medium (supernatant from pcDNA3.1(+)-transfected HEK cell—untreated) for 24 h. For each experiment, 10 pictures of the migrating cells were taken, and a representative picture was depicted (Bars  =  50 μm). (D) Cells were treated as described in (C). To quantitatively measure cell motility, Giemsa-stained migrating cells from the lower side of the membrane were lyzed, and absorbance was measured at a wavelength of 560 nm. Values represent means and SD of three independently performed experiments. **p≤0.01 and ***p≤0.001 as calculated using two-tailed non-parametric Mann-Whitney test. (E) The silencing effect of the Orai1-targeting shRNAmir-pGIPZ vectors was analyzed by immunoblot in lentiviral-transduced HEK cells. 48 h after transduction, cells were lysed and 100 mg of lysates were loaded per line. β-actin serves as a loading control. (F) Cell migration of indicated shRNAmir-transduced HEK cells was assessed using Boyden chamber assay. *Right panels*: HEK cells were transduced with scrambled or Orai1-targeting ShRNA, and 48 h later, cells were incubated for 24 h in the presence of 100 ng/ml of cl-CD95L or untreated (supernatant of pcDNA3.1(+)-transfected HEK cells). Migrating cells were fixed with methanol and stained by Giemsa. For each experiment, five pictures of random fields were taken, and a representative picture was depicted (Bars = 70 mm). *Left panel*: To quantitatively measure cell motility, Giemsa-stained migrating cells from the lower side of the membrane were lyzed, and absorbance was measured at a wavelength of 560 nm. Values represent means and SD of three independently performed experiments. *** *p*≤0.001 as calculated using two-tailed non-parametric Mann-Whitney test.

**Fig 5:**
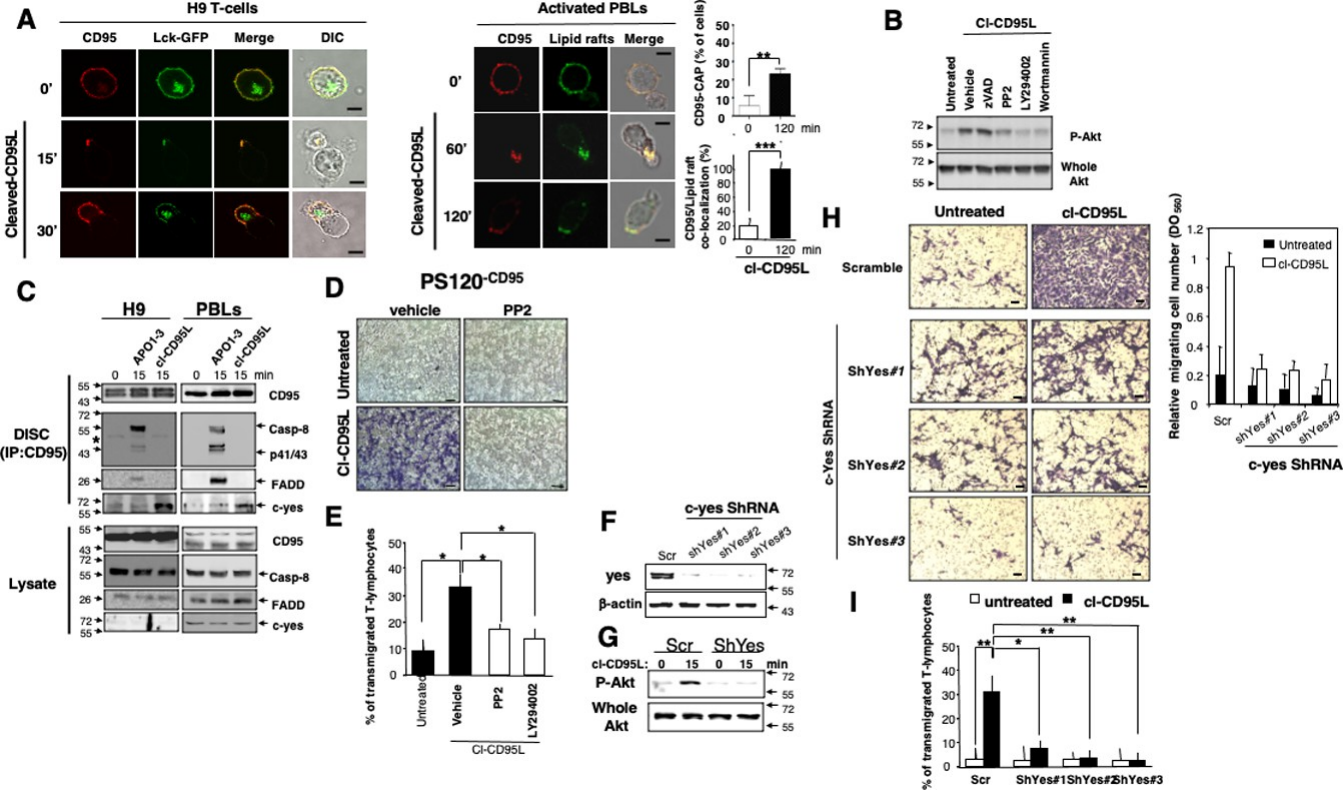
The src kinase c-yes orchestrates the cl-CD95L-mediated PI3K/Akt signaling pathway and cell migration. (A) *Left panels*: The leukemic cell line H9 was transiently transfected with Lck-GFP. 24 h after transfection, living cells were treated or untreated for the indicated times in the presence of cl-CD95L (100 ng/ml). 0 min (0’) condition corresponds to cells analyzed prior to the addition of 100 ng/mL of cl-CD95L. CD95 was stained using a mouse anti-CD95 mAb and a goat Alexa555-coupled anti-mouse IgG mAb. *Right panels*: Activated PBLs were incubated for the indicated times with 100 ng/ml of cl-CD95L, and then cells were fixed. Lipid rafts were stained using Alexa488-cholera toxin B subunit and CD95 was followed as previously mentioned. Images were acquired with a confocal microscope with a 63× objective. 0 min (0’) condition corresponds to cells analyzed prior to the addition of 100 ng/mL of cl-CD95L. Cell morphology was followed using phase contrast microscopy (Bars = 7.5 mm). The percentage of T-cells displaying a CD95 cluster was assessed (300 cells counted for each condition). Among the activated T-lymphocytes showing CD95 cluster, the amount of CD95-CAP co-localized with lipid rafts was assessed. (B) H9 T-cells were pre-incubated for 30 min with DMSO (vehicle), 10 mM of PP2, 5 mM of LY294002 (LY), 2.5 mM of wortmannin (Wort), or 40 mM of zVAD-fmk (zV) and then treated for 15 min with 100 ng/ml of cl-CD95L. Untreated condition corresponds to cells incubated with supernatant of pcDNA3.1(+)-transfected HEK cells. Cells were lyzed and Akt phosphorylation (S^473^) and whole Akt were assessed by immunoblots. (C) The H9 T-cell line (*left panels*) and activated PBLs (*right panels*) were incubated for indicated times with 100 ng/ml of cl-CD95L or APO1-3. APO1-3 is an agonistic anti-CD95 mAb incubated for 15 min with indicated cells to activate CD95 and trigger DISC formation. Then, cells were lyzed and CD95 was immunoprecipitated. The 0 min condition corresponds to unstimulated cells directly lyzed to next immunoprecipitate CD95. APO1-3 also serves for the immunoprecipitation step of CD95 (see Materials and Methods). The immune complex was resolved in a 10% SDS-PAGE and indicated immunoblots were performed. Total lysates were depicted to confirm that the same amount of protein was present for each immunoprecipitation. The black stars represent heavy chains of Ig. (D) Boyden chamber assays were performed as described in Materials and Methods. PS120^CD95^ (All PS120 cells have been reconstituted with wild type NHE1 (see Materials and Methods) were pre-incubated with or without a non-cytotoxic and non-cytostatic concentration of PP2 (10 mM) or DMSO (vehicle) for 30 min and then treated (cl-CD95L) or untreated (Untreated condition corresponds to cells incubated with supernatant of pcDNA3.1(+)-transfected HEK cells) for 24 h with 100 ng/ml of cl-CD95L. Data are representative of three independently performed experiments. The vehicle conditions in the presence or absence of cl-CD95L also serve in S9B Fig to decipher the effect of zVAD-fmk. (E) Activated PBLs were pre-incubated with 10 mM of the src inhibitor PP2, 5 mM of the PI3K inhibitor LY294002 (LY) or DMSO (vehicle) and then incubated in the presence of cl-CD95L (100 ng/ml) for 24 h. Untreated cells correspond to activated PBLs incubated for 24h with supernatant of pcDNA3.1(+)-transfected HEK cells. Then, endothelial transmigration of PBLs was assessed as described in Materials and Methods. (F) The silencing effect of the c-yes-targeting shRNAmir-pGIPZ vectors was analyzed by immunoblot in lentiviral-transduced PS120^CD95^ cells. 72 h after transduction, cells were lysed and 100 mg of lysate was loaded per line. β-actin serves as loading control. (G) The T-cell line H9 was transduced with scramble (scr) or c-yes (shYes#3)-targeting shRNAmir-containing lentivirus. 72 h after transduction, living cells were harvested and green cells were sorted by flow cytometry using FACSAria. Cells were immediately stimulated with cl-CD95L (100 ng/ml) (15 min) or left untreated (0 min), and the amounts of Akt phosphorylation (S^473^) were assessed by immunoblot. (H) Cell migration of indicated shRNAmir-transduced PS120^CD95^ was assessed using the Boyden chamber assay. *Left panels*: 72 h after transduction, cells were treated (cl-CD95L) or untreated (supernatant of pcDNA3.1(+)-transfected HEK cells) with 100 ng/ml of cl-CD95L for 24 h. For each experiment, five pictures of the migrating cells were taken, and a representative picture was depicted (Bars = 50 mm). *Right panel*: To quantitatively measure cell motility, Giemsa-stained migrating cells from the lower side of the membrane were lyzed and absorbance was measured at a wavelength of 560 nm. Values represent means and SD of three independently performed experiments. (I) H9 cells were transduced as mentioned in (G), and the green cells were incubated in the presence of cl-CD95L (100 ng/ml) or supernatant of pcDNA3.1(+)-transfected HEK cells (untreated) for 24 h. Then, the endothelial transmigration of the sh-RNA-expressing T-cells was assessed as described in Materials and Methods.

**Fig 6:**
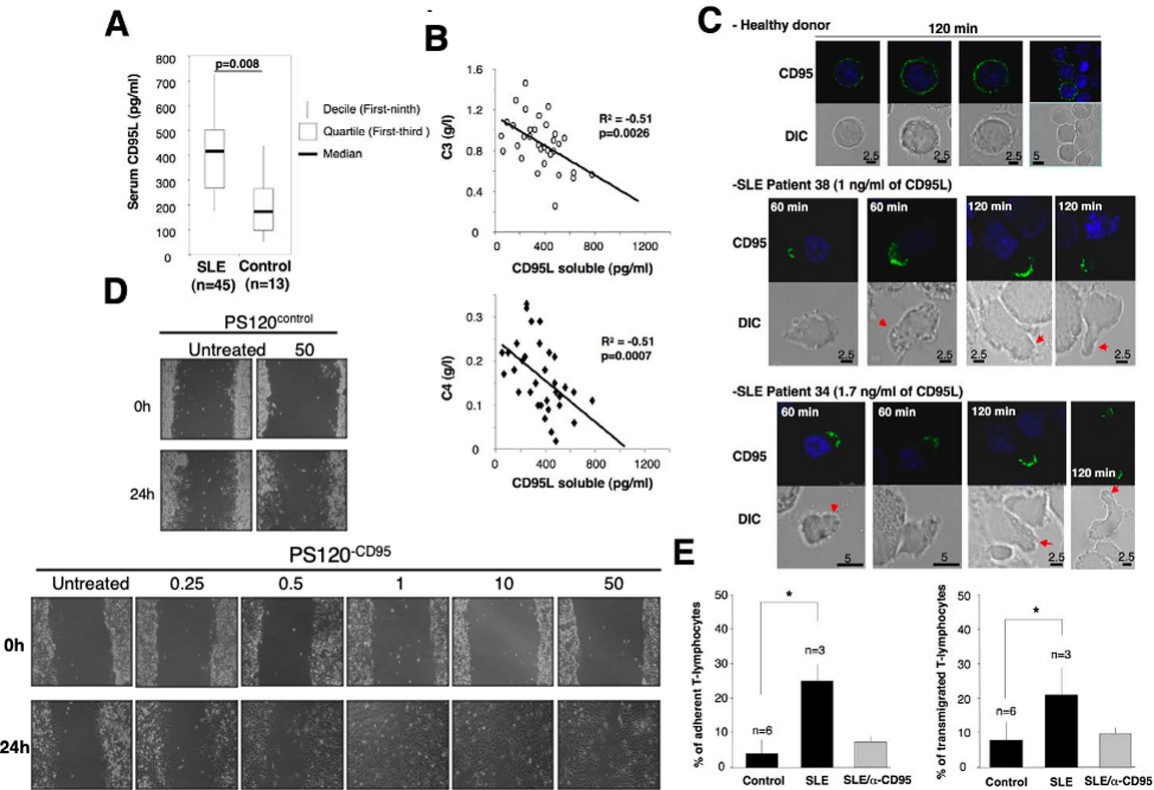
CD95L found in SLE patients promotes endothelial transmigration of activated PBLs. (A) By ELISA, the amounts of soluble CD95L in sera of healthy donors (control) and SLE patients were quantified. *n* stands for the number of SLE patients and healthy subjects. (B) Correlation between aggravation markers of the disease and the amount of soluble CD95L. (C) Activated PBLs were incubated for the indicated times with sera of healthy donors or SLE patients and the distribution of CD95 was analyzed by confocal microscopy. (D) All PS120 cells have been reconstituted with wild type NHE1 (see Materials and Methods). PS120^control^ cells do not express CD95, while its counterpart PS120^CD95^ expresses a full length human CD95 (see Materials and Methods). A 24 h wound healing assay was performed. The fibroblastic cell line PS120^control^ or its CD95-expressing counterpart PS120^CD95^ was grown to confluence. then the monolayer was “wounded” by scratching cells using a pipette tip. Using microscopy, the migration was monitored for 24 h with the indicated concentrations of cl-CD95L (ng/mL) as indicated in Materials and Methods. The untreated condition corresponds to cells incubated with a control medium (supernatant of pcDNA3.1(+)-transfected HEK cells). Pictures are representative of 3 independently performed experiments. (E) Activated PBLs were incubated in the presence of sera from SLE patients (*n*  =  3) or healthy donors (*n*  =  6), and then, adhesion to endothelial cells (*left panel*) and endothelial transmigration (*right panel*) of PBLs were assessed as described in Materials and Methods. Where indicated, activated PBLs were preincubated 30 min with the antagonist anti-CD95 mAb ZB4 (α-CD95). ZB4 was maintained in the culture at 10 μg/ml.

## Supporting information

S1 Fig The naturally processed CD95L fails to trigger caspase-8 activation and cell death. (A) The human embryonic kidney epithelial cell line 293T (HEK) was transfected with wild type CD95L or the mutated CD95L^S126E/L127E^. Cells were lyzed (Cell) and supernatants were harvested (Sn) 5 d after transfection. Dead cells were eliminated from the supernatant by centrifugation (2×4,000 rpm for 15 min), and then exosomes were pelleted using ultracentrifugation (100,000 g/2 h) and lyzed (Exo.). White arrowhead indicates cleaved-CD95L. Black arrowhead depicts full-length CD95L and the star indicates an unknown processed CD95L product. (B) The molecular size of cleaved CD95L was analyzed using size exclusion S-200-HR Sephacryl columns (Amersham Pharmacia, Orsay, France). Fractions were harvested and cl-CD95L was dosed by ELISA. (C) Jurkat and H9 T-cells were treated with 100 ng/ml of the home-made Ig-CD95L (dodecameric) or the naturally processed CD95L (trimeric), and activation of caspase-8 was assessed by following the cleavage of the protease using immunoblot (fragments p41/43 and p18). For each cell, long exposures of the film are depicted to detect the p18 cleaved fragment. Stars indicate irrelevant bands. (D) Indicated cells were incubated for 24 h with an engineered dodecameric Ig-CD95L or the naturally processed homotrimeric CD95L and cell death was assessed by MTT assay.
(TIF)

S2 Fig Cleaved CD95L induces formation of CD95-Cap at the extremity of the emitted pseudopod. (A) The leukemic T-cell line H9 was incubated for indicated times with 100 ng/ml of cleaved CD95L or a control medium (supernatant of pcDNA3.1(+)-transfected HEK cells—untreated), and after extensive washing, cells were fixed. CD95 was stained using an anti-CD95 mAb (APO1-3) and revealed using the secondary Alexa488-conjugated goat anti-mouse antibody (Invitrogen, Carlsbad, CA, USA). Number of cells harboring a CD95-Cap was counted (at least 300 cells counted for each condition). (B) The leukemic T-cell lines CEM and H9 were incubated for 30 min with 100 ng/ml of cleaved CD95L or a control medium (supernatant of pcDNA3.1(+)-transfected HEK cells -untreated), and the plasma membrane distribution of CD95 was then analyzed as described in (A). The quantity of cells harboring a CD95-Cap was assessed by counting (300 cells/condition). Values represent means and SD of three independently performed experiments. **p<0.01 and ***p<0.001 as calculated using non-parametric and two-tailed Mann-Whitney test. (C) Leukemic T-cells H9 and CEM were untreated (supernatant of pcDNA3.1(+)-transfected HEK cells) or treated for 30 min with cleaved CD95L (100 ng/ml), and after extensive washing, cells were fixed. CD95 was stained using an anti-CD95 mAb (APO1-3) and revealed using a secondary Alexa488-conjugated goat anti-mouse antibody. Nuclei were stained with DAPI (blue). Slides were washed with PBS, dried, and mounted with Fluoromount (Cliniscience SAS, Montrouge, France). Red arrows depict emitted pseudopods upon CD95 engagement. Images were acquired with a confocal microscope TSC SP5 (Leica, Wetzlar, Germany) with a ApoPLAN 63× objective.
(TIF)

S3 Fig CD95 and truncated CD95(1–210)-expressing PS120 clones. All PS120 cells are reconstituted with wild type NHE1 (see Materials and Methods). (A) *Upper panel*: analysis of the CD95 expression at the surface of the indicated PS120 clones stably expressing either the CD95 wild type or its death domain truncated counterpart (Δ1–210). Cells were stained with an anti-CD95 mAb (clone DX2), washed, and a PE-coupled goat anti-mouse secondary antibody was used to reveal plasma membrane CD95 by flow cytometry. *Lower panel*: for each staining, the mean of the fluorescence intensity (MFI), which is correlated to the amount of plasma membrane CD95, was depicted. FACS analyses of PS120 cells shown in (A) have been partly reused in [3]. (B) Indicated PS120 clones expressing either empty vector (PS120^control^), truncated (PS120^CD95(Δ1–210)^), or wild type CD95 (PS120^CD95^) were incubated with the cytotoxic Ig-CD95L or the cleaved CD95L for 24 h and cell death was quantified using viability assay MTT.
(TIF)

S4 Fig Cleaved CD95L triggers a pseudopod-localized Ca^2+^ rise. *Video imaging on living cells*: Before application of cl-CD95L, activated T-lymphocytes were loaded with the Ca^2+^ indicator Fura-2AM along with EGTA-AM (1 mM, 30 min, room temperature), a slow high-affinity Ca^2+^ buffer. Under these conditions, Ca^2+^ entering the cell would bind rapidly to the Ca^2+^ probe, producing a fluorescent signal, and then be captured by EGTA. Activated T-lymphocytes were bathed in an external medium containing 2 mM Ca^2+^ and stimulated with 100 ng/ml of cl-CD95L just after the capture of the ratio images (0 s). Pictures were recorded at the indicated times following the addition of cl-CD95L to assess the formation of localized Ca^2+^ influx. Emitted pseudopods were depicted using a white square, and cell migration is indicated by a white arrow. Grey levels were translated to false colors according to a scale shown on the right.
(TIF)

S5 Fig The cl-CD95L-mediated calcium response occurs through a PLCγ1/IP3-R-dependent process. (A) The T-cell line Jurkat was incubated for the indicated times with 100 ng/ml of cl-CD95L, and then cells were lyzed. 100 μg of protein was loaded per lane and resolved in a 10% SDS-PAGE. Immunoblots were performed using indicated mAbs (Cell Signaling technology, Ozyme, Saint Quentin, France). (B) *Left panel*: The PLC-γ1-deficient T-cell Jurkat and its PLC-γ1-reconstituted counterpart were loaded with the calcium probe Fura2-AM (1 μM, 30 min at RT) and then stimulated with 100 ng/ml of cl-CD95L. Ratio images were taken every 10 s. Regions of Interest (ROIs) outlining individual cells were defined in three independent experiments, and the mean ratio in each ROI was plotted versus time. Ratio values were converted to [Ca^2+^]i values using a calibration curve (see Materials and Methods section). *Right panel*: Statistical analyses of the AUC values for the indicated T-cells. *** *p* ≤0.001 using non-parametric two-tailed Mann-Whitney test. (C, D) *Left panels*: Jurkat (C) and H9 (D) T-cells were loaded as previously mentioned. Cells were incubated with the IP3-R inhibitor, 2-APB (20 μM) during the recording as indicated by the unfilled rectangle or DMSO (control). Then cells were stimulated with 100 ng/ml of cl-CD95L (black arrow) and the intracellular Ca^2+^ was recorded every 10 s. Regions of Interest (ROIs) outlining individual cells were defined in three independently performed experiments and the mean ratio in each ROI was plotted versus time. Ratio values were converted to [Ca^2+^]i values using a calibration curve (see Materials and Methods section). Values depict the mean ± SD of the area under the curve (AUC) calculated for 1,000 s. *Right panels*: statistical analyses of the AUC values. The control H9 values served in S7A and S7B Fig to compare the effect of the calcium chelator BAPTA-AM on the CD95-mediated intracellular Ca^2+^ response. *** *p* value≤0.001 using non-parametric two-tailed Mann-Whitney test.
(TIF)

S6 Fig Extracellular Ca^2+^ plays a crucial role in the cl-CD95L-mediated Ca^2+^ rise. (A) Indicated T-cells were loaded with 1 μM of the calcium probe Fura-2AM for 30 min at RT. Cells were bathed at 37°C in a medium containing 2 mM Ca^2+^ (2 mM [Ca^2+^]_e_) or a Ca^2+^-free medium (0 mM [Ca^2+^]_e_) and then treated with supernatant of pcDNA3.1(+)-transfected HEK cells (Untreated) or with 100 ng/ml of cl-CD95L. Values were recorded every 10 s. Regions of Interest (ROIs) outlining individual cells were defined in three independently performed experiments and the mean ratio in each ROI was plotted versus time. Ratio values were converted to [Ca^2+^]i values using a calibration curve (see Materials and Methods section). For each treatment, the mean ± SD of the area under the curve (AUC) measured for 1,000 s in individual cells is depicted. Basal and maximal levels of [Ca^2+^]i are indicated by red and blue dotted lines, respectively. (B) Statistical analyses of the AUC values for indicated cells incubated in the presence of cl-CD95L in a regular (2 mM) or a Ca^2+^-free medium (0 mM). Using non-parametric two-tailed Mann-Whitney test, *** indicates a *p* value ≤0.001. (C) PBLs were untreated (DMSO, upper panel) or treated (lower panel) with the SOC channel inhibitor BTP-2 (500 nM) and then 100 ng/ml of cl-CD95L was added. For each condition, the mean ± SD of the area under the curve (AUC) measured for 1,000 s is depicted. Basal and maximal levels of [Ca^2+^]i are indicated by red and blue dotted lines, respectively. Note that individual calcium oscillations and/or synchronous calcium oscillations (upper panel) lead to apparent calcium oscillations during the plateau phase.
(TIF)

S7 Fig BAPTA-AM abrogates the cl-CD95L-mediated Ca2+ response. (A) H9 T-cells were loaded with 1 mM of the calcium probe Fura-2AM for 30 min at RT. Cells were pre-incubated or not (DMSO) with BAPTA-AM (5 mM) and then stimulated with 100 ng/ml of cl-CD95L. Values were recorded every 10 s. Regions of Interest (ROIs) outlining individual cells were defined in three independently performed experiments, and the mean ratio in each ROI was plotted versus time. Ratio values were converted to [Ca^2+^]i values using a calibration curve (see Materials and Methods section). For each treatment, the mean ± SD of the area under the curve (AUC) measured for 1,000 s is depicted. (B) Statistical analyses of the AUC values for H9 T-cells treated or untreated (DMSO) with 5 mM of BAPTA-AM and stimulated with cl-CD95L. Using non-parametric two-tailed Mann-Whitney test, *** indicates a *p* ≤0.001. The control H9 values (DMSO) served in S5D Fig to compare the effect of the calcium chelator BAPTA-AM on the CD95-mediated intracellular Ca^2+^ response.
(TIF)

S8 Fig Identification of the p110 isoform eliciting the cl-CD95L-mediated cell motility. (A) The table depicts IC50 (nM) of the different isoform-selective inhibitors on the class I PI3Ks. PI3K-α Inh-IV (3-(4-Morpholinothieno[3,2-d]pyrimidin-2-yl)phenol); PI3K-β Inh-VI/TGX-221 ((±)-7-Methyl-2-(morpholin-4-yl)-9-(1-phenylaminoethyl)-pyrido[1,2-a]-pyrimidin-4-one); PI3K-γ Inh (5-Quinoxalin-6-ylmethylene-thiazolidine-2,4-dione); PI3K-δ Inh-X, IC87114; and Wortmannin and LY294002 were tested to ascertain the isoform involved in the cl-CD95L-mediated motility. All the inhibitors came from Calbiochem (Merck Chemicals Ltd., Nottingham, UK). Note: Data were compiled from [4, 5]. (B, C, D, and E) For each inhibitor, the concentrations used in the different *in cellulo* assays were defined according to the respective isoform-selective IC50 indicated in bold in (A). The T-cell line H9 (B) and the fibroblastic cell line PS120^CD95^ (C) were pre-incubated with DMSO (0), the indicated concentrations of the pan-PI3K inhibitors wortmannin and LY294002 or the isoform-selective inhibitors PI3K-α Inh-IV (α); PI3K-β inh-VI (β); PI3K-γ Inh (γ); and PI3K-δ Inh-X (δ) for 60 min and then untreated (supernatant of pcDNA3.1(+)-transfected HEK cells; U) or treated for 30 min with 100 ng/ml of cl-CD95L. Cells were lyzed and 100 μg of protein was loaded for each lane. Indicated immunoblots were performed. Bands were scanned and a densitometry analysis was performed using ImageJ. Values below the immunoblots indicate the percentage of phospho-Akt inhibition reached with each selective inhibitor. Data are representative of three independently performed experiments. (D) H9 T-cells were pre-incubated with a concentration of the indicated PI3K inhibitors corresponding to 100-fold their predicted IC50 (see A) or with DMSO (control) for 60 min, and then cells were incubated with cl-CD95L (100 ng/ml) or with supernatant of pcDNA3.1(+)-transfected HEK cells (Mock) for 24 h in a Boyden Chamber in which the porous membrane was covered with a confluent monolayer of endothelial cells (see Materials and Methods). Then, the endothelial transmigration of the T-cells was assessed as described in Materials and Methods. (E) The fibroblastic PS120^CD95^ cell line was pre-incubated with a concentration of the indicated PI3K inhibitors corresponding to 100-fold their predicted IC50 (see A) or with DMSO (control) for 60 min, and then cells were incubated with cl-CD95L (100 ng/ml) or with supernatant of pcDNA3.1(+)-transfected HEK cells (Mock) for 24 h in a Boyden Chamber assay. To quantitatively measure cell motility, Giemsa-stained migrating cells from the lower side of the membrane were lyzed and absorbance was measured at a wavelength of 560 nm. Values represent means and SEM of three independently performed experiments.
(TIF)

S9 Fig The caspase activity does not participate in the cl-CD95L-induced cell motility. (A) *Wound healing assays*: A confluent monolayer of the indicated adherent cells was wounded with a tip. Then, cells were pre-incubated for 30 min with 40 μM of zVAD-fmk or DMSO (vehicle) and treated for 24 h with 100 ng/ml of cl-CD95L or with a supernatant of pcDNA3.1(+)-transfected HEK cells (untreated) and pictures were acquired (Bars  =  50 μm). Pictures are representative of three independently performed experiments. (B) *Upper panels*: The CD95-expressing PS120 cells were pre-incubated with the pan-caspase inhibitor zVAD-fmk (40 μM) or DMSO (vehicle) for 30 min and then seeded in the upper compartment of the Boyden chamber in a low FCS (1%)-containing medium. Cells were incubated for 24 h with 100 ng/ml of cl-CD95L or with a supernatant of pcDNA3.1(+)-transfected HEK cells (untreated). Then the membrane was removed, the upper side containing the non-migrating cells was wiped out with cotton-tipped swabs, and migrating cells in the opposite side of the filter were fixed with methanol and stained by Giemsa (purple cells). For each experiment, five pictures of random fields were taken and a representative picture was depicted (Bars  =  70 μm). The images of PS120^CD95^ cells untreated/vehicle and cl-CD95L/vehicle came from the same experiment to decipher the effect of PP2 (src inhibitor) and zVAD (caspase inhibitor) and were used in S9B and 5D Figs. *Lower panel*: To quantify cell motility, Giemsa-stained migrating cells from the lower side of the membrane were lyzed and absorbance was measured at 560 nm. Data represent means and SD of three independently performed experiments.
(TIF)

S10 Fig The src kinase c-yes is instrumental in the cl-CD95L-induced cell motility. (A) *Wound healing assays*: A confluent monolayer of PS120^CD95^ adherent cells was wounded with a tip. Cells were pre-incubated with or without (DMSO-vehicle) 10 mM of the src inhibitor PP2 and then were treated or untreated (supernatant of pcDNA3.1(+)-transfected HEK cells) for 24 h with 100 ng/ml of cl-CD95L. Pictures were acquired to assess the efficiency of the wound healing. Dotted red lines delineate the initial position of the wound. (B) PS120^CD95^ cells were transduced with lentiviral particles containing a c-yes-targeting shRNAmir or a scramble shRNAmir (OpenBiosystem, USA). 48 h after transduction, the confluent monolayer of cells was wounded and cell migration was analyzed in the presence of 100 ng/ml of cl-CD95L for the indicated time. Pictures are representative of three independently performed experiments.
(TIF)

S1 File Underlying image data for the western blot experiments in Figs 2, 4, 5, S1, S5 and S8. (PPTX)

S2 File Supplementary information for Materials and Methods section. (DOCX)
