# Supplementary figures and images for: Correction: The Naturally Processed CD95L Elicits a c-Yes/Calcium/PI3K-Driven Cell Migration Pathway
Source: PLoS Biol. 2023 Feb 23;21(2):e3002027. doi: 10.1371/journal.pbio.3002027 (PMC9949890; doi:10.1371/journal.pbio.3002027)

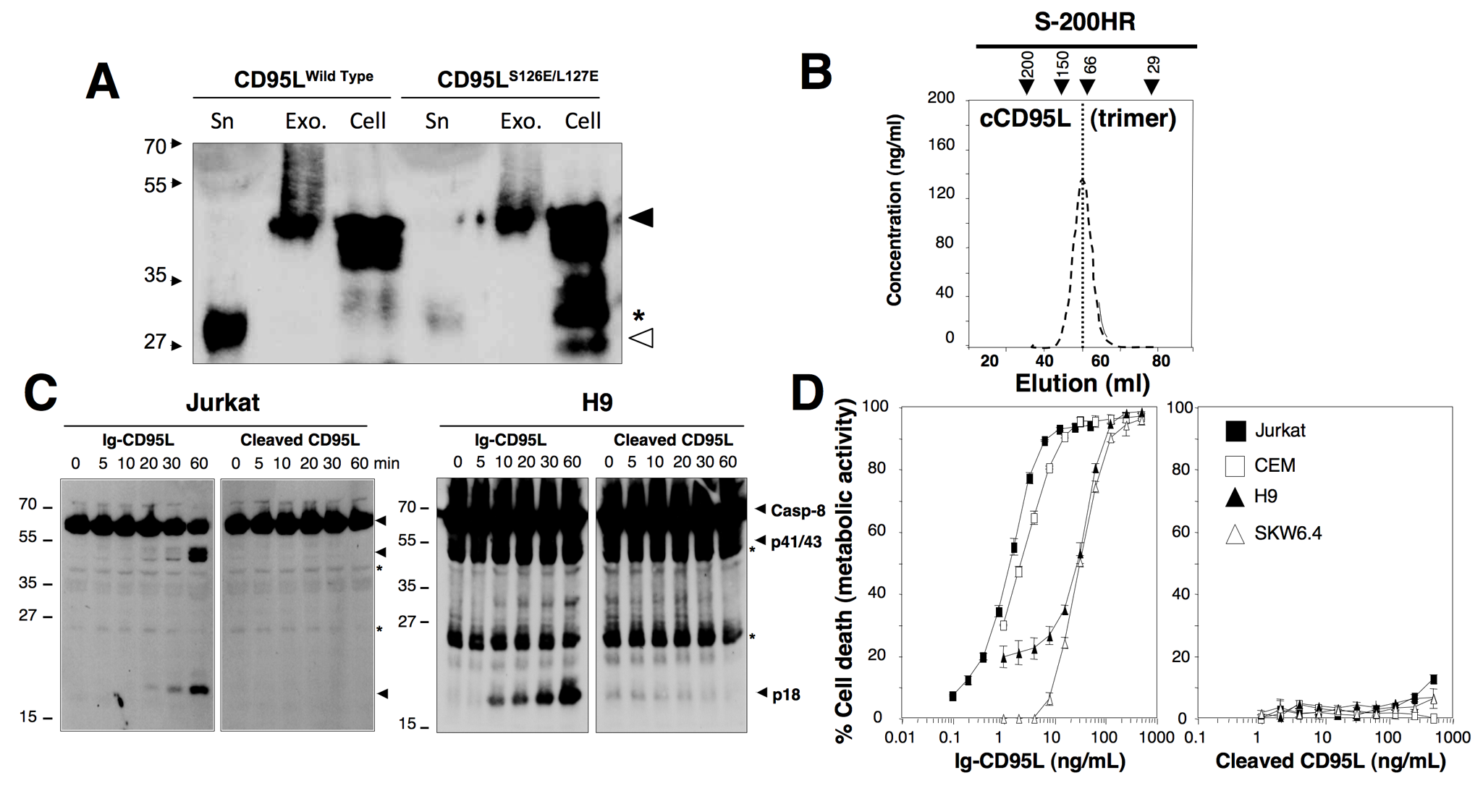

Supplement: S1 Fig — (A) The human embryonic kidney epithelial cell line 293T (HEK) was transfected with wild type CD95L or the mutated CD95LS126E/L127E. Cells were lyzed (Cell) and supernatants were harvested (Sn) 5 d after transfection. Dead cells were eliminated from the supernatant by centrifugation (2×4,000 rpm for 15 min), and then exosomes were pelleted using ultracentrifugation (100,000 g/2 h) and lyzed (Exo.). White arrowhead indicates cleaved-CD95L. Black arrowhead depicts full-length CD95L and the star indicates an unknown processed CD95L product. (B) The molecular size of cleaved CD95L was analyzed using size exclusion S-200-HR Sephacryl columns (Amersham Pharmacia, Orsay, France). Fractions were harvested and cl-CD95L was dosed by ELISA. (C) Jurkat and H9 T-cells were treated with 100 ng/ml of the home-made Ig-CD95L (dodecameric) or the naturally processed CD95L (trimeric), and activation of caspase-8 was assessed by following the cleavage of the protease using immunoblot (fragments p41/43 and p18). For each cell, long exposures of the film are depicted to detect the p18 cleaved fragment. Stars indicate irrelevant bands. (D) Indicated cells were incubated for 24 h with an engineered dodecameric Ig-CD95L or the naturally processed homotrimeric CD95L and cell death was assessed by MTT assay. (TIF) [file pbio.3002027.s001.tif]

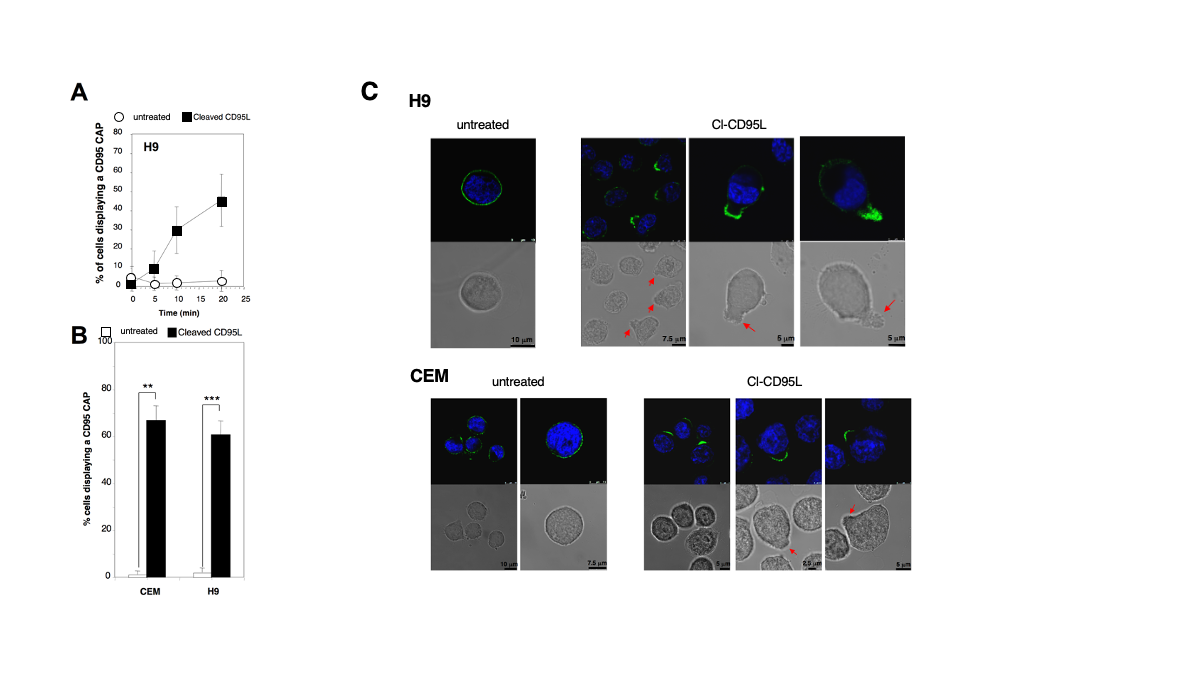

Supplement: S2 Fig — (A) The leukemic T-cell line H9 was incubated for indicated times with 100 ng/ml of cleaved CD95L or a control medium (supernatant of pcDNA3.1(+)-transfected HEK cells—untreated), and after extensive washing, cells were fixed. CD95 was stained using an anti-CD95 mAb (APO1-3) and revealed using the secondary Alexa488-conjugated goat anti-mouse antibody (Invitrogen, Carlsbad, CA, USA). Number of cells harboring a CD95-Cap was counted (at least 300 cells counted for each condition). (B) The leukemic T-cell lines CEM and H9 were incubated for 30 min with 100 ng/ml of cleaved CD95L or a control medium (supernatant of pcDNA3.1(+)-transfected HEK cells -untreated), and the plasma membrane distribution of CD95 was then analyzed as described in (A). The quantity of cells harboring a CD95-Cap was assessed by counting (300 cells/condition). Values represent means and SD of three independently performed experiments. **p<0.01 and ***p<0.001 as calculated using non-parametric and two-tailed Mann-Whitney test. (C) Leukemic T-cells H9 and CEM were untreated (supernatant of pcDNA3.1(+)-transfected HEK cells) or treated for 30 min with cleaved CD95L (100 ng/ml), and after extensive washing, cells were fixed. CD95 was stained using an anti-CD95 mAb (APO1-3) and revealed using a secondary Alexa488-conjugated goat anti-mouse antibody. Nuclei were stained with DAPI (blue). Slides were washed with PBS, dried, and mounted with Fluoromount (Cliniscience SAS, Montrouge, France). Red arrows depict emitted pseudopods upon CD95 engagement. Images were acquired with a confocal microscope TSC SP5 (Leica, Wetzlar, Germany) with a ApoPLAN 63× objective. (TIF) [file pbio.3002027.s002.tif]

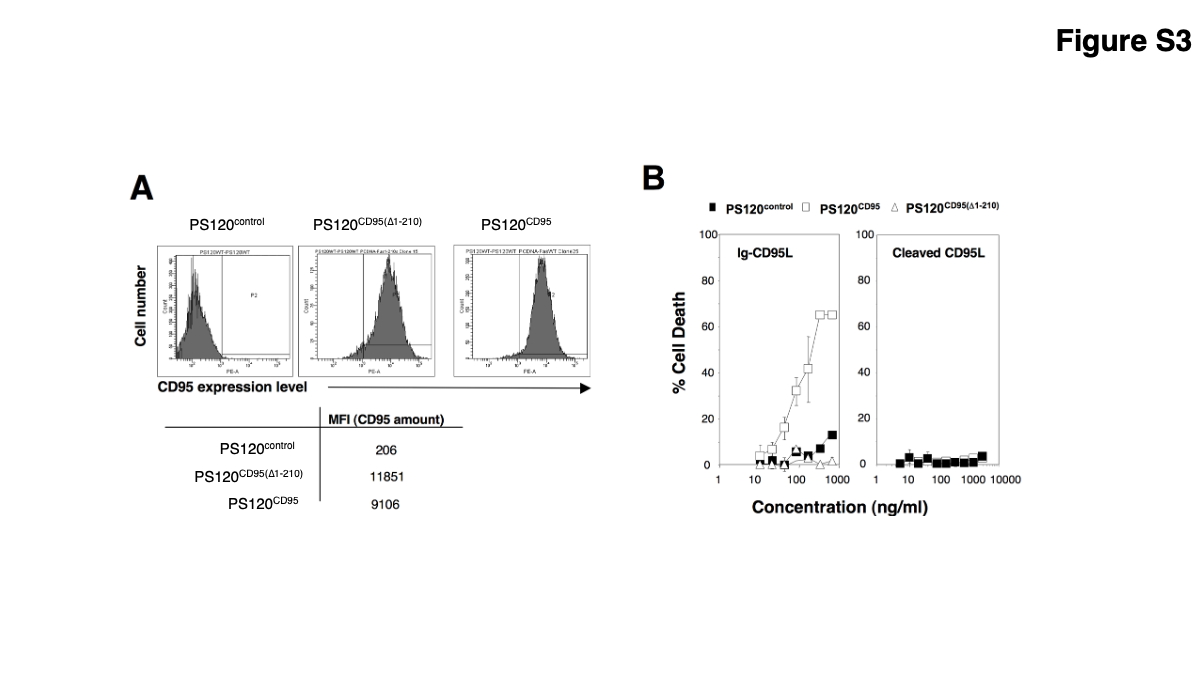

Supplement: S3 Fig — All PS120 cells are reconstituted with wild type NHE1 (see Materials and Methods). (A) Upper panel: analysis of the CD95 expression at the surface of the indicated PS120 clones stably expressing either the CD95 wild type or its death domain truncated counterpart (Δ1–210). Cells were stained with an anti-CD95 mAb (clone DX2), washed, and a PE-coupled goat anti-mouse secondary antibody was used to reveal plasma membrane CD95 by flow cytometry. Lower panel: for each staining, the mean of the fluorescence intensity (MFI), which is correlated to the amount of plasma membrane CD95, was depicted. FACS analyses of PS120 cells shown in (A) have been partly reused in [3]. (B) Indicated PS120 clones expressing either empty vector (PS120control), truncated (PS120CD95(Δ1–210)), or wild type CD95 (PS120CD95) were incubated with the cytotoxic Ig-CD95L or the cleaved CD95L for 24 h and cell death was quantified using viability assay MTT. (TIF) [file pbio.3002027.s003.tif]

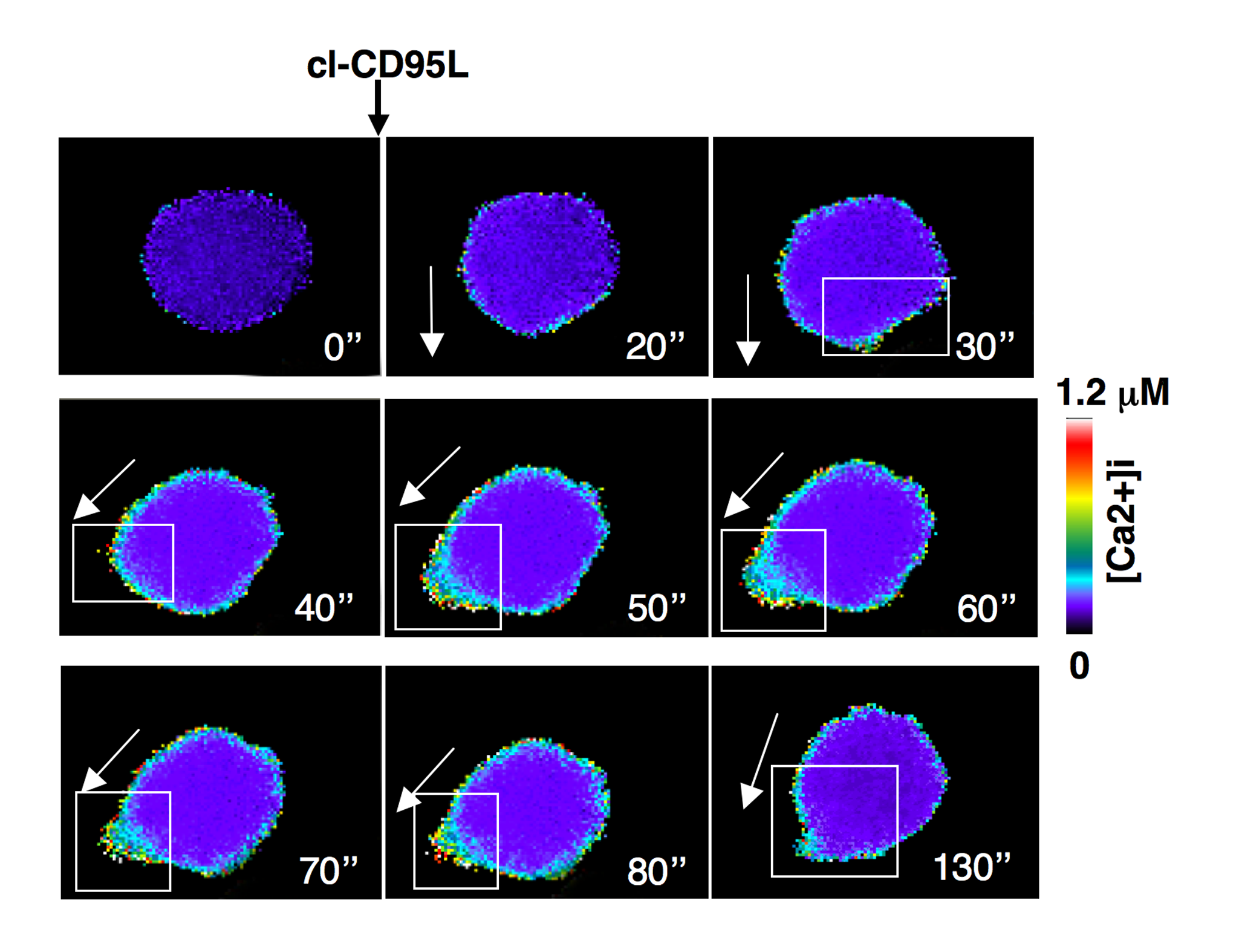

Supplement: S4 Fig — Video imaging on living cells: Before application of cl-CD95L, activated T-lymphocytes were loaded with the Ca2+ indicator Fura-2AM along with EGTA-AM (1 mM, 30 min, room temperature), a slow high-affinity Ca2+ buffer. Under these conditions, Ca2+ entering the cell would bind rapidly to the Ca2+ probe, producing a fluorescent signal, and then be captured by EGTA. Activated T-lymphocytes were bathed in an external medium containing 2 mM Ca2+ and stimulated with 100 ng/ml of cl-CD95L just after the capture of the ratio images (0 s). Pictures were recorded at the indicated times following the addition of cl-CD95L to assess the formation of localized Ca2+ influx. Emitted pseudopods were depicted using a white square, and cell migration is indicated by a white arrow. Grey levels were translated to false colors according to a scale shown on the right. (TIF) [file pbio.3002027.s004.tif]

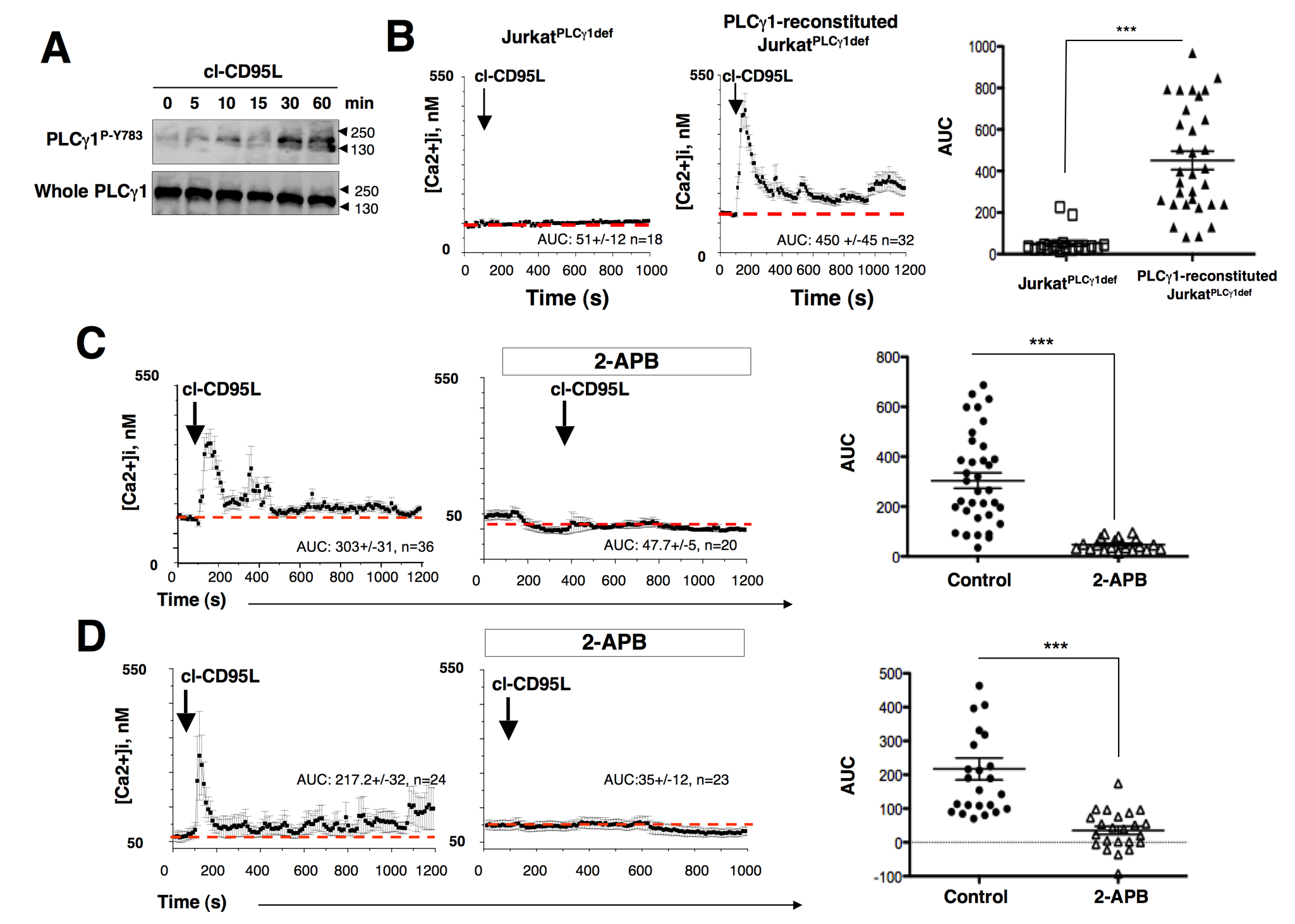

Supplement: S5 Fig — (A) The T-cell line Jurkat was incubated for the indicated times with 100 ng/ml of cl-CD95L, and then cells were lyzed. 100 μg of protein was loaded per lane and resolved in a 10% SDS-PAGE. Immunoblots were performed using indicated mAbs (Cell Signaling technology, Ozyme, Saint Quentin, France). (B) Left panel: The PLC-γ1-deficient T-cell Jurkat and its PLC-γ1-reconstituted counterpart were loaded with the calcium probe Fura2-AM (1 μM, 30 min at RT) and then stimulated with 100 ng/ml of cl-CD95L. Ratio images were taken every 10 s. Regions of Interest (ROIs) outlining individual cells were defined in three independent experiments, and the mean ratio in each ROI was plotted versus time. Ratio values were converted to [Ca2+]i values using a calibration curve (see Materials and Methods section). Right panel: Statistical analyses of the AUC values for the indicated T-cells. *** p ≤0.001 using non-parametric two-tailed Mann-Whitney test. (C, D) Left panels: Jurkat (C) and H9 (D) T-cells were loaded as previously mentioned. Cells were incubated with the IP3-R inhibitor, 2-APB (20 μM) during the recording as indicated by the unfilled rectangle or DMSO (control). Then cells were stimulated with 100 ng/ml of cl-CD95L (black arrow) and the intracellular Ca2+ was recorded every 10 s. Regions of Interest (ROIs) outlining individual cells were defined in three independently performed experiments and the mean ratio in each ROI was plotted versus time. Ratio values were converted to [Ca2+]i values using a calibration curve (see Materials and Methods section). Values depict the mean ± SD of the area under the curve (AUC) calculated for 1,000 s. Right panels: statistical analyses of the AUC values. The control H9 values served in S7A and S7B Fig to compare the effect of the calcium chelator BAPTA-AM on the CD95-mediated intracellular Ca2+ response. *** p value≤0.001 using non-parametric two-tailed Mann-Whitney test. (TIF) [file pbio.3002027.s005.tif]

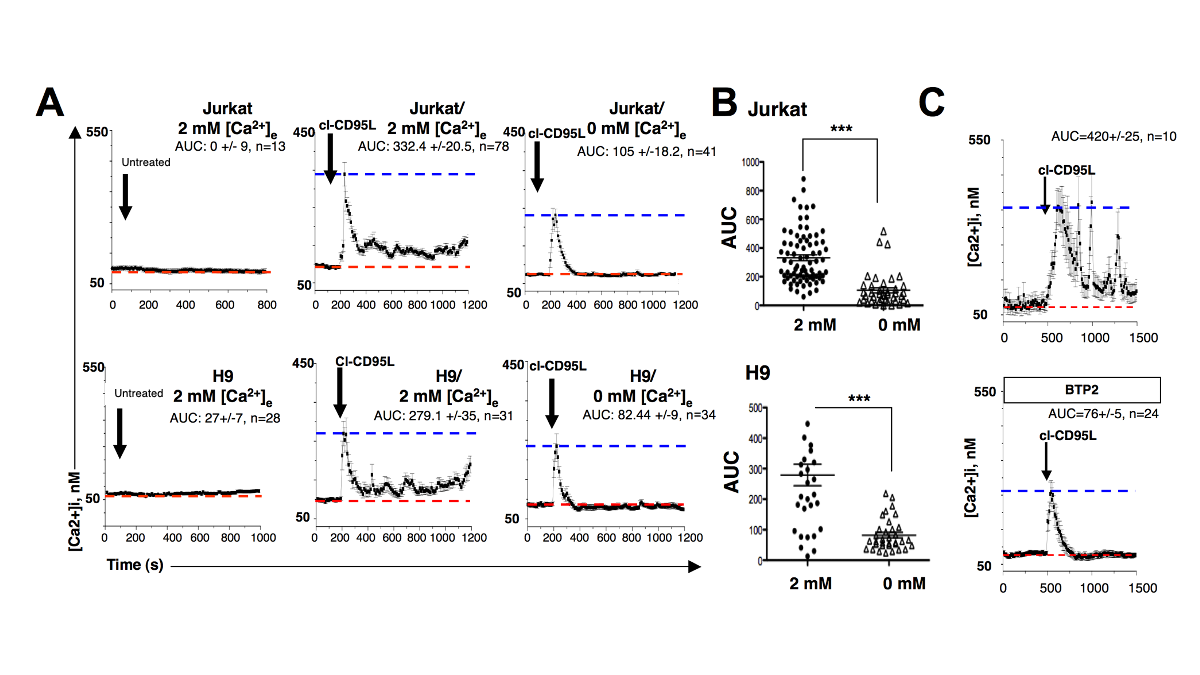

Supplement: S6 Fig — (A) Indicated T-cells were loaded with 1 μM of the calcium probe Fura-2AM for 30 min at RT. Cells were bathed at 37°C in a medium containing 2 mM Ca2+ (2 mM [Ca2+]e) or a Ca2+-free medium (0 mM [Ca2+]e) and then treated with supernatant of pcDNA3.1(+)-transfected HEK cells (Untreated) or with 100 ng/ml of cl-CD95L. Values were recorded every 10 s. Regions of Interest (ROIs) outlining individual cells were defined in three independently performed experiments and the mean ratio in each ROI was plotted versus time. Ratio values were converted to [Ca2+]i values using a calibration curve (see Materials and Methods section). For each treatment, the mean ± SD of the area under the curve (AUC) measured for 1,000 s in individual cells is depicted. Basal and maximal levels of [Ca2+]i are indicated by red and blue dotted lines, respectively. (B) Statistical analyses of the AUC values for indicated cells incubated in the presence of cl-CD95L in a regular (2 mM) or a Ca2+-free medium (0 mM). Using non-parametric two-tailed Mann-Whitney test, *** indicates a p value ≤0.001. (C) PBLs were untreated (DMSO, upper panel) or treated (lower panel) with the SOC channel inhibitor BTP-2 (500 nM) and then 100 ng/ml of cl-CD95L was added. For each condition, the mean ± SD of the area under the curve (AUC) measured for 1,000 s is depicted. Basal and maximal levels of [Ca2+]i are indicated by red and blue dotted lines, respectively. Note that individual calcium oscillations and/or synchronous calcium oscillations (upper panel) lead to apparent calcium oscillations during the plateau phase. (TIF) [file pbio.3002027.s006.tif]

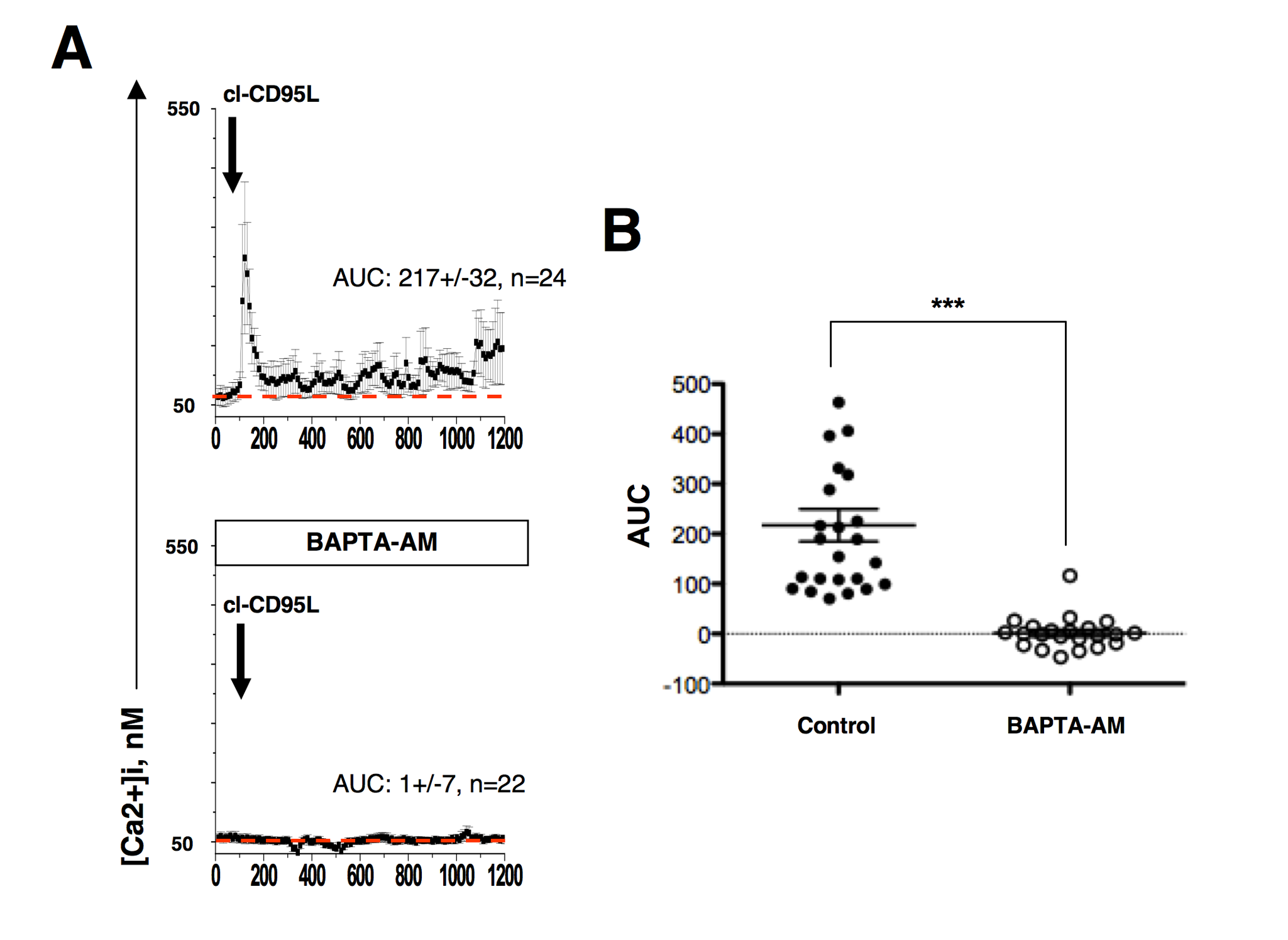

Supplement: S7 Fig — (A) H9 T-cells were loaded with 1 mM of the calcium probe Fura-2AM for 30 min at RT. Cells were pre-incubated or not (DMSO) with BAPTA-AM (5 mM) and then stimulated with 100 ng/ml of cl-CD95L. Values were recorded every 10 s. Regions of Interest (ROIs) outlining individual cells were defined in three independently performed experiments, and the mean ratio in each ROI was plotted versus time. Ratio values were converted to [Ca2+]i values using a calibration curve (see Materials and Methods section). For each treatment, the mean ± SD of the area under the curve (AUC) measured for 1,000 s is depicted. (B) Statistical analyses of the AUC values for H9 T-cells treated or untreated (DMSO) with 5 mM of BAPTA-AM and stimulated with cl-CD95L. Using non-parametric two-tailed Mann-Whitney test, *** indicates a p ≤0.001. The control H9 values (DMSO) served in S5D Fig to compare the effect of the calcium chelator BAPTA-AM on the CD95-mediated intracellular Ca2+ response. (TIF) [file pbio.3002027.s007.tif]

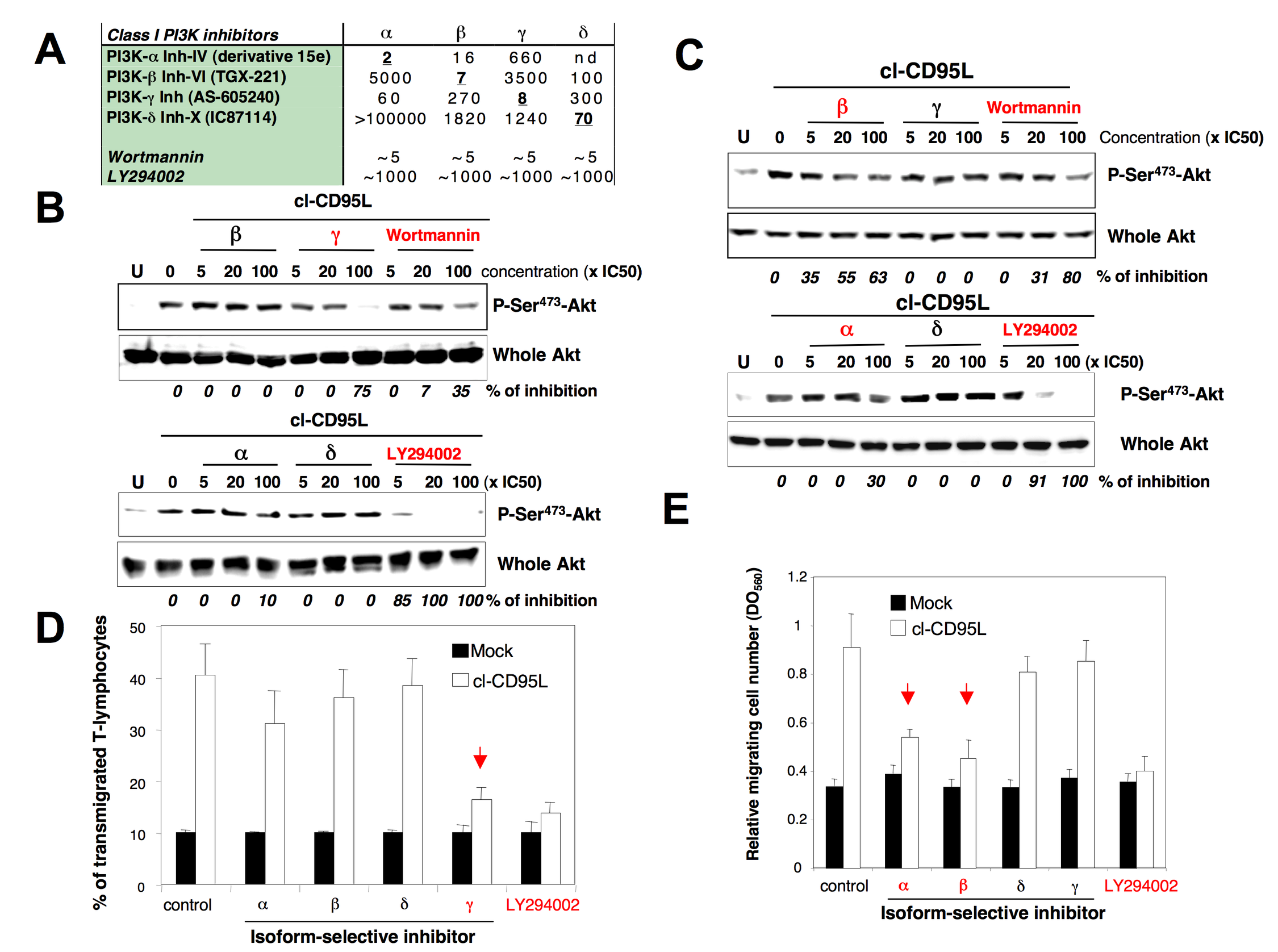

Supplement: S8 Fig — (A) The table depicts IC50 (nM) of the different isoform-selective inhibitors on the class I PI3Ks. PI3K-α Inh-IV (3-(4-Morpholinothieno[3,2-d]pyrimidin-2-yl)phenol); PI3K-β Inh-VI/TGX-221 ((±)-7-Methyl-2-(morpholin-4-yl)-9-(1-phenylaminoethyl)-pyrido[1,2-a]-pyrimidin-4-one); PI3K-γ Inh (5-Quinoxalin-6-ylmethylene-thiazolidine-2,4-dione); PI3K-δ Inh-X, IC87114; and Wortmannin and LY294002 were tested to ascertain the isoform involved in the cl-CD95L-mediated motility. All the inhibitors came from Calbiochem (Merck Chemicals Ltd., Nottingham, UK). Note: Data were compiled from [4, 5]. (B, C, D, and E) For each inhibitor, the concentrations used in the different in cellulo assays were defined according to the respective isoform-selective IC50 indicated in bold in (A). The T-cell line H9 (B) and the fibroblastic cell line PS120CD95 (C) were pre-incubated with DMSO (0), the indicated concentrations of the pan-PI3K inhibitors wortmannin and LY294002 or the isoform-selective inhibitors PI3K-α Inh-IV (α); PI3K-β inh-VI (β); PI3K-γ Inh (γ); and PI3K-δ Inh-X (δ) for 60 min and then untreated (supernatant of pcDNA3.1(+)-transfected HEK cells; U) or treated for 30 min with 100 ng/ml of cl-CD95L. Cells were lyzed and 100 μg of protein was loaded for each lane. Indicated immunoblots were performed. Bands were scanned and a densitometry analysis was performed using ImageJ. Values below the immunoblots indicate the percentage of phospho-Akt inhibition reached with each selective inhibitor. Data are representative of three independently performed experiments. (D) H9 T-cells were pre-incubated with a concentration of the indicated PI3K inhibitors corresponding to 100-fold their predicted IC50 (see A) or with DMSO (control) for 60 min, and then cells were incubated with cl-CD95L (100 ng/ml) or with supernatant of pcDNA3.1(+)-transfected HEK cells (Mock) for 24 h in a Boyden Chamber in which the porous membrane was covered with a confluent monolayer of endothelial cells (see Materials [file pbio.3002027.s008.tif]

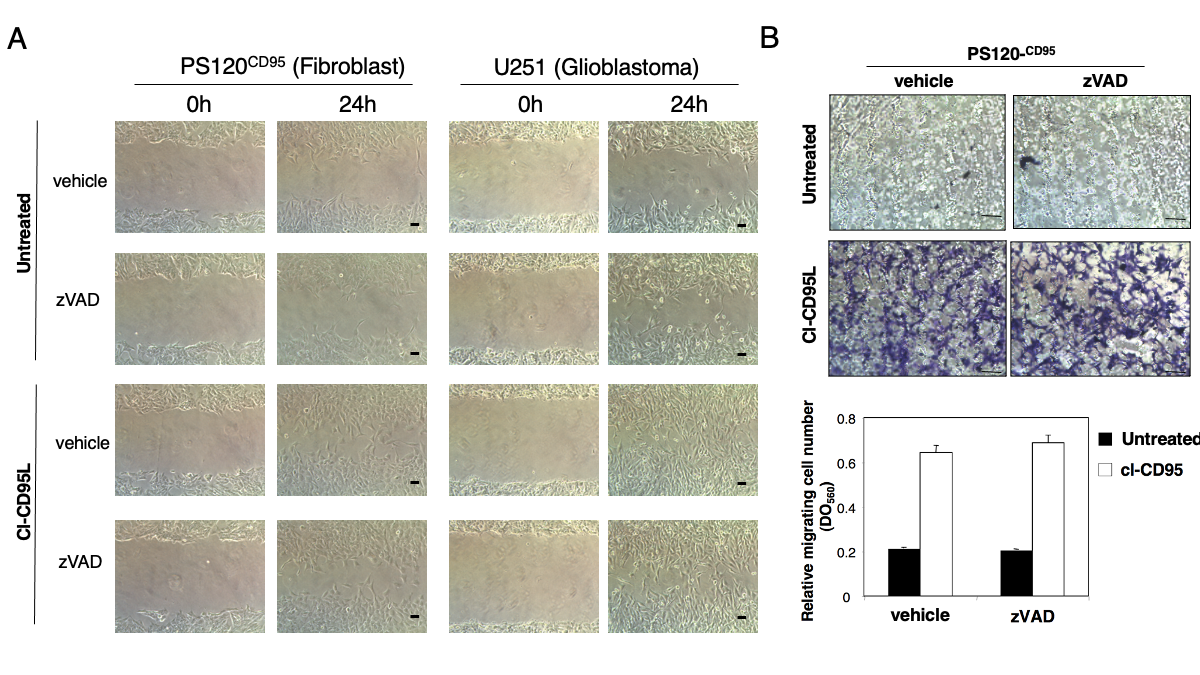

Supplement: S9 Fig — (A) Wound healing assays: A confluent monolayer of the indicated adherent cells was wounded with a tip. Then, cells were pre-incubated for 30 min with 40 μM of zVAD-fmk or DMSO (vehicle) and treated for 24 h with 100 ng/ml of cl-CD95L or with a supernatant of pcDNA3.1(+)-transfected HEK cells (untreated) and pictures were acquired (Bars = 50 μm). Pictures are representative of three independently performed experiments. (B) Upper panels: The CD95-expressing PS120 cells were pre-incubated with the pan-caspase inhibitor zVAD-fmk (40 μM) or DMSO (vehicle) for 30 min and then seeded in the upper compartment of the Boyden chamber in a low FCS (1%)-containing medium. Cells were incubated for 24 h with 100 ng/ml of cl-CD95L or with a supernatant of pcDNA3.1(+)-transfected HEK cells (untreated). Then the membrane was removed, the upper side containing the non-migrating cells was wiped out with cotton-tipped swabs, and migrating cells in the opposite side of the filter were fixed with methanol and stained by Giemsa (purple cells). For each experiment, five pictures of random fields were taken and a representative picture was depicted (Bars = 70 μm). The images of PS120CD95 cells untreated/vehicle and cl-CD95L/vehicle came from the same experiment to decipher the effect of PP2 (src inhibitor) and zVAD (caspase inhibitor) and were used in S9B and 5D Figs. Lower panel: To quantify cell motility, Giemsa-stained migrating cells from the lower side of the membrane were lyzed and absorbance was measured at 560 nm. Data represent means and SD of three independently performed experiments. (TIF) [file pbio.3002027.s009.tif]

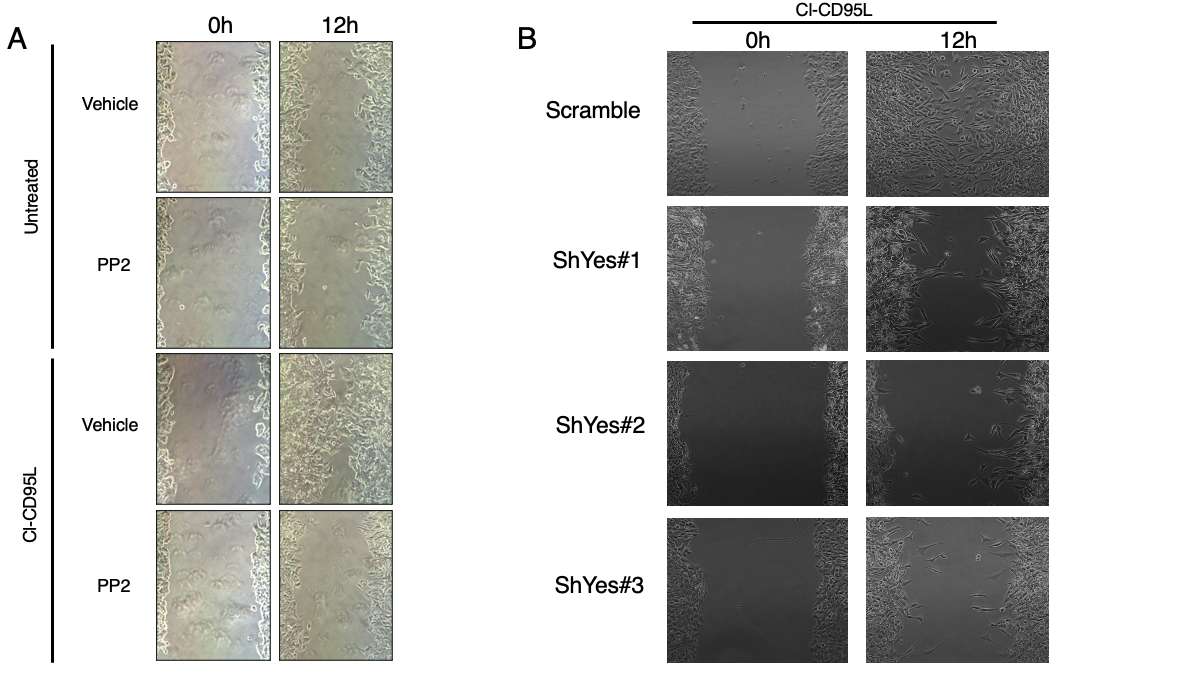

Supplement: S10 Fig — (A) Wound healing assays: A confluent monolayer of PS120CD95 adherent cells was wounded with a tip. Cells were pre-incubated with or without (DMSO-vehicle) 10 mM of the src inhibitor PP2 and then were treated or untreated (supernatant of pcDNA3.1(+)-transfected HEK cells) for 24 h with 100 ng/ml of cl-CD95L. Pictures were acquired to assess the efficiency of the wound healing. Dotted red lines delineate the initial position of the wound. (B) PS120CD95 cells were transduced with lentiviral particles containing a c-yes-targeting shRNAmir or a scramble shRNAmir (OpenBiosystem, USA). 48 h after transduction, the confluent monolayer of cells was wounded and cell migration was analyzed in the presence of 100 ng/ml of cl-CD95L for the indicated time. Pictures are representative of three independently performed experiments. (TIF) [file pbio.3002027.s010.tif]
