# Supplementary material for: Correction: The Naturally Processed CD95L Elicits a c-Yes/Calcium/PI3K-Driven Cell Migration Pathway
Source: PLoS Biol. 2023 Feb 23;21(2):e3002027. doi: 10.1371/journal.pbio.3002027 (PMC9949890; doi:10.1371/journal.pbio.3002027)
Supplement: S1 File — (PPTX) [file pbio.3002027.s011.pptx]

## Slide 1
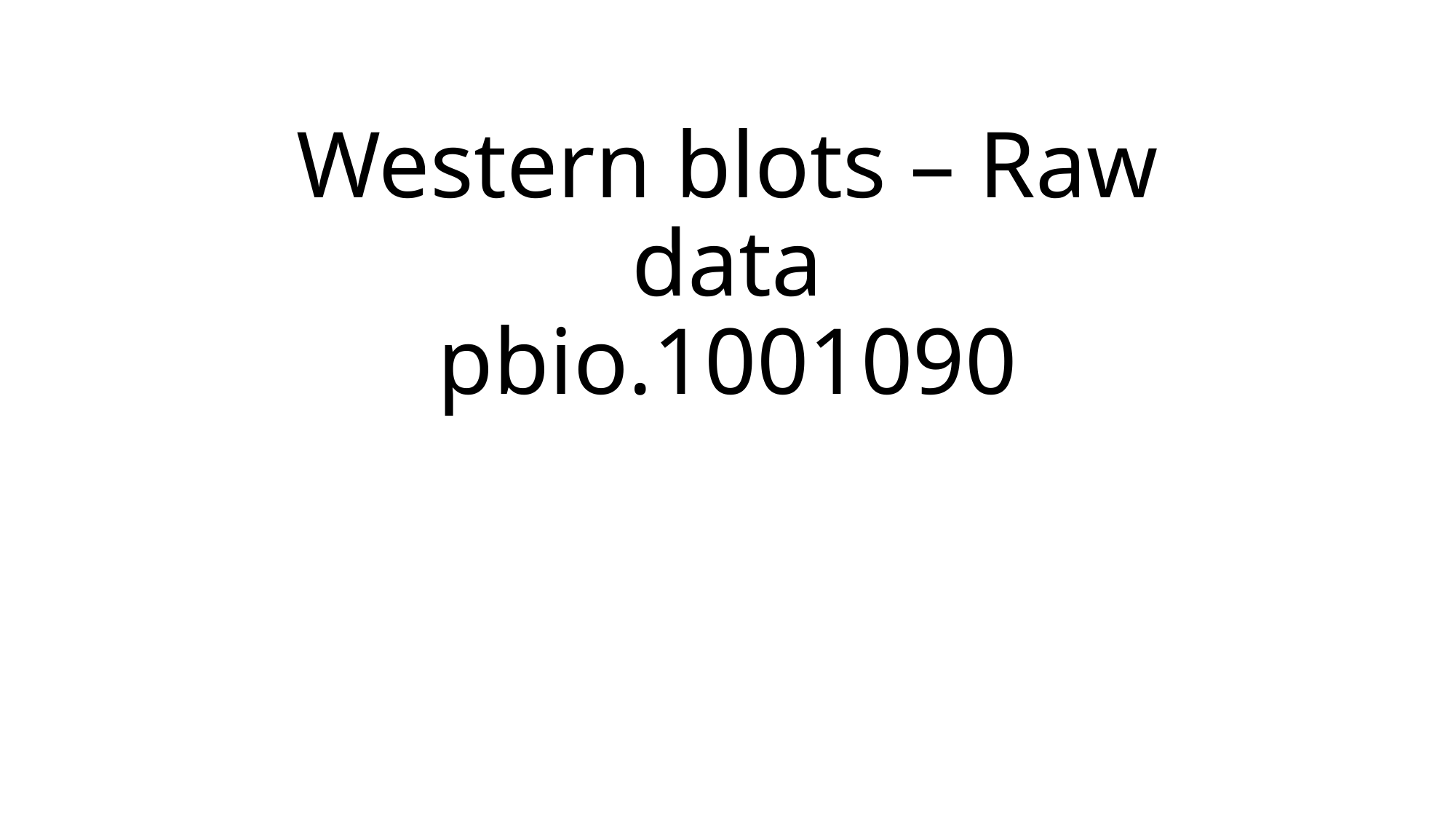

# Western blots – Raw data pbio.1001090

## Slide 2
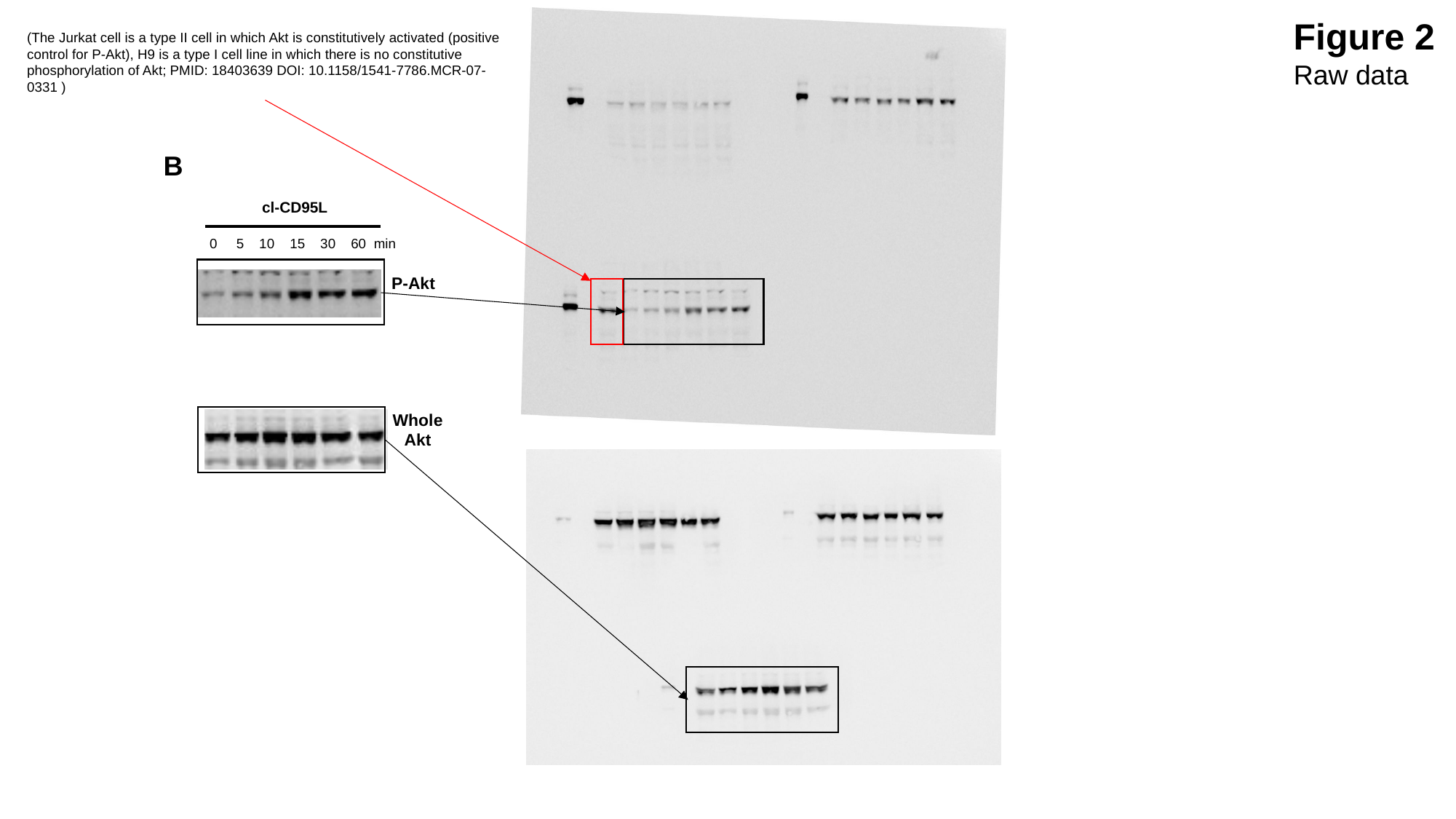

Figure 2
(The Jurkat cell is a type II cell in which Akt is constitutively activated (positive control for P-Akt), H9 is a type I cell line in which there is no constitutive phosphorylation of Akt; PMID: 18403639 DOI: 10.1158/1541-7786.MCR-07-0331 )
Raw data
B
cl-CD95L
0 5 10 15 30 60 min
P-Akt
Whole
Akt

## Slide 3
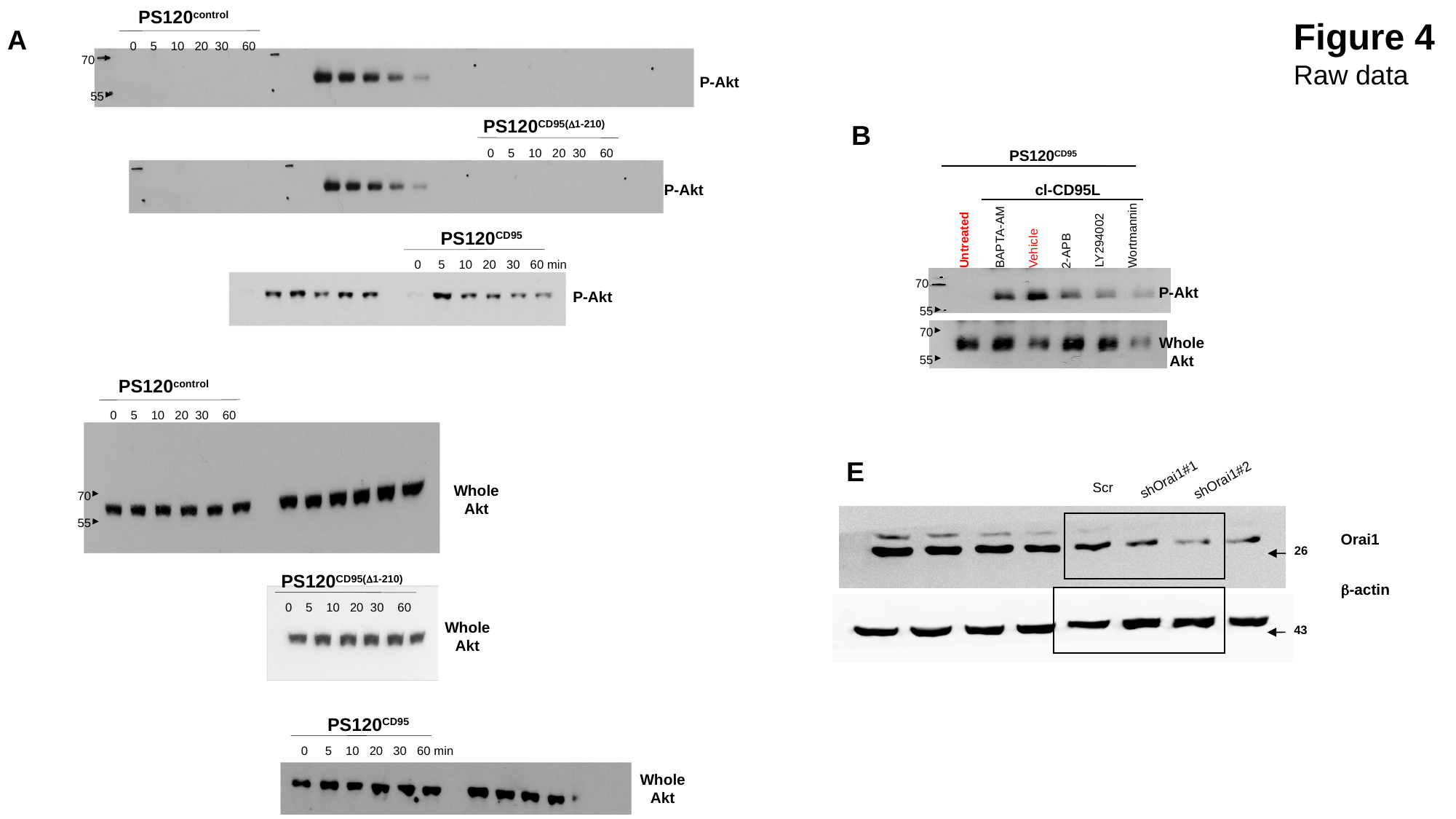

PS120control
Figure 4
A
0 5 10 20 30 60
70
Raw data
P-Akt
55
PS120CD95(Δ1-210)
B
0 5 10 20 30 60
PS120CD95
P-Akt
cl-CD95L
PS120CD95
Wortmannin
BAPTA-AM
Untreated
LY294002
Vehicle
2-APB
0 5 10 20 30 60 min
70
P-Akt
P-Akt
55
70
Whole
Akt
55
PS120control
0 5 10 20 30 60
E
shOrai1#1
shOrai1#2
Whole
Akt
Scr
70
55
Orai1
26
PS120CD95(Δ1-210)
β-actin
0 5 10 20 30 60
Whole
Akt
43
PS120CD95
0 5 10 20 30 60 min
Whole
Akt

## Slide 4
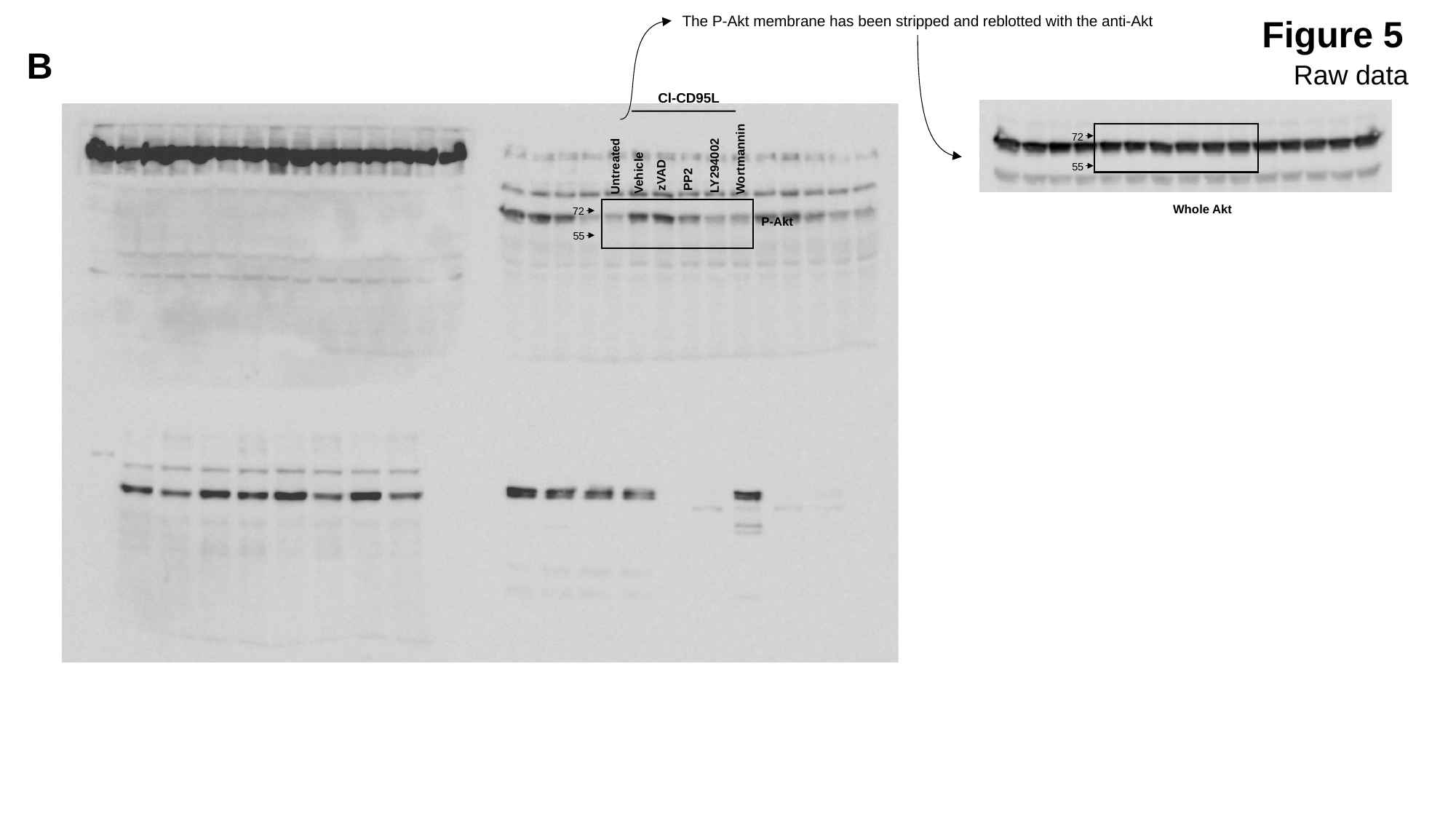

The P-Akt membrane has been stripped and reblotted with the anti-Akt
Figure 5
B
Raw data
Cl-CD95L
72
Wortmannin
LY294002
Untreated
55
Vehicle
zVAD
PP2
Whole Akt
72
P-Akt
55

## Slide 5
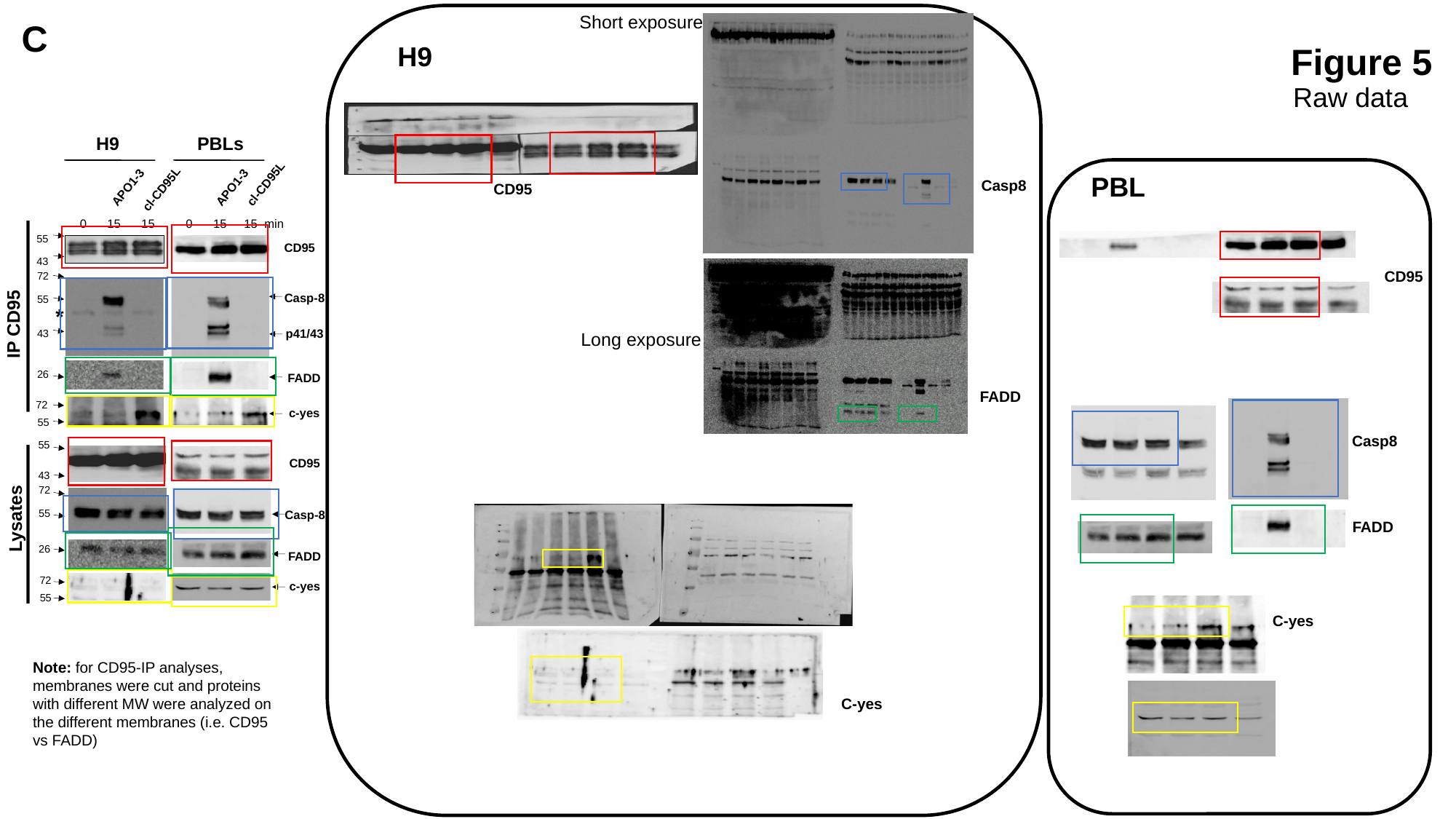

Short exposure
C
H9
Figure 5
Raw data
H9
PBLs
PBL
cl-CD95L
Casp8
CD95
APO1-3
APO1-3
cl-CD95L
0 15 15 0 15 15 min
55
43
CD95
CD95
72
Casp-8
55
*
IP CD95
p41/43
43
Long exposure
26
FADD
FADD
72
c-yes
55
Casp8
55
CD95
43
72
55
Lysates
Casp-8
FADD
26
FADD
72
c-yes
55
C-yes
C-yes
Note: for CD95-IP analyses, membranes were cut and proteins with different MW were analyzed on the different membranes (i.e. CD95 vs FADD)

## Slide 6
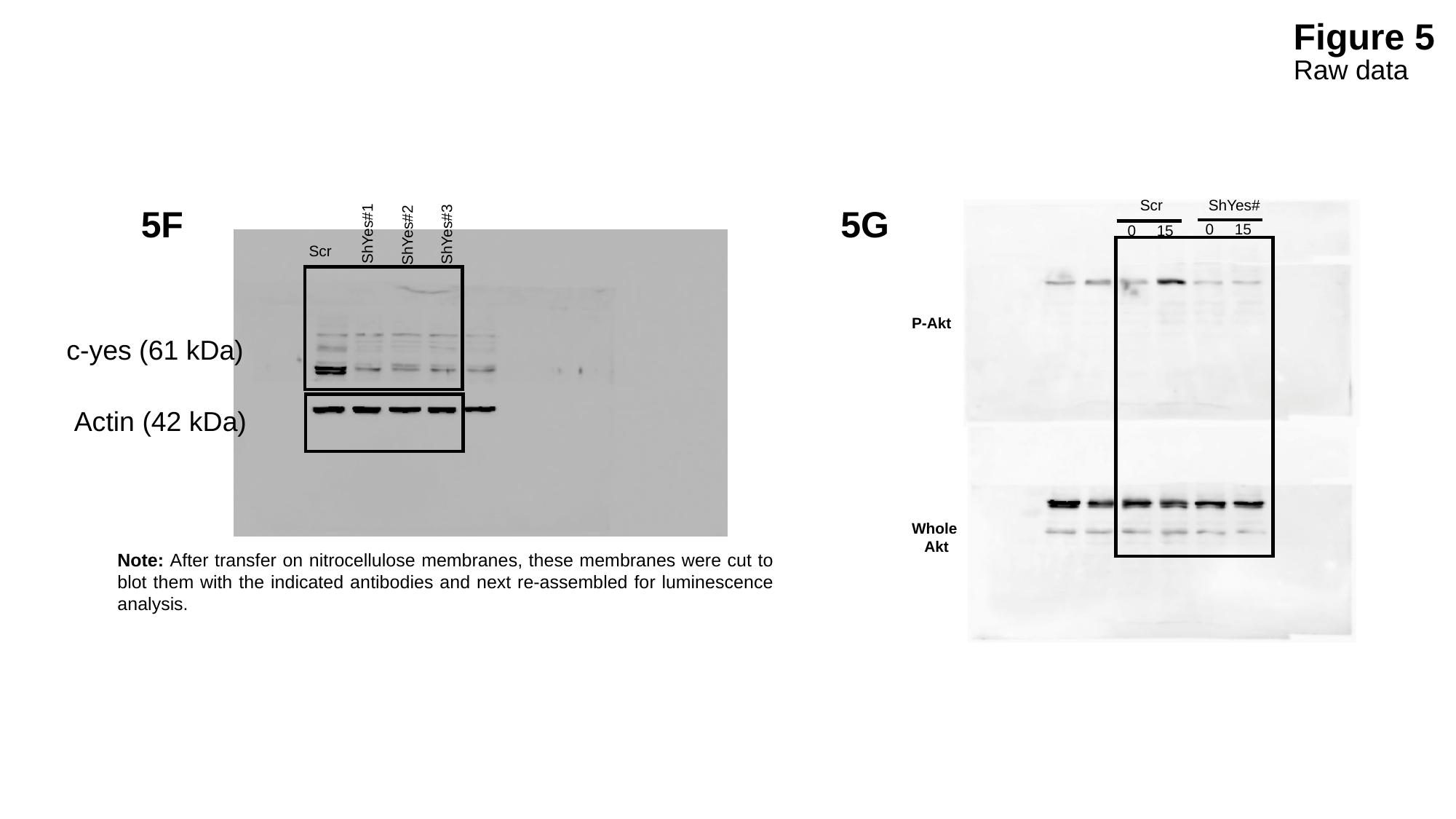

Figure 5
Raw data
Scr
ShYes#
5F
5G
0 15
0 15
ShYes#1
ShYes#3
ShYes#2
Scr
P-Akt
c-yes (61 kDa)
Actin (42 kDa)
Whole
Akt
Note: After transfer on nitrocellulose membranes, these membranes were cut to blot them with the indicated antibodies and next re-assembled for luminescence analysis.

## Slide 7
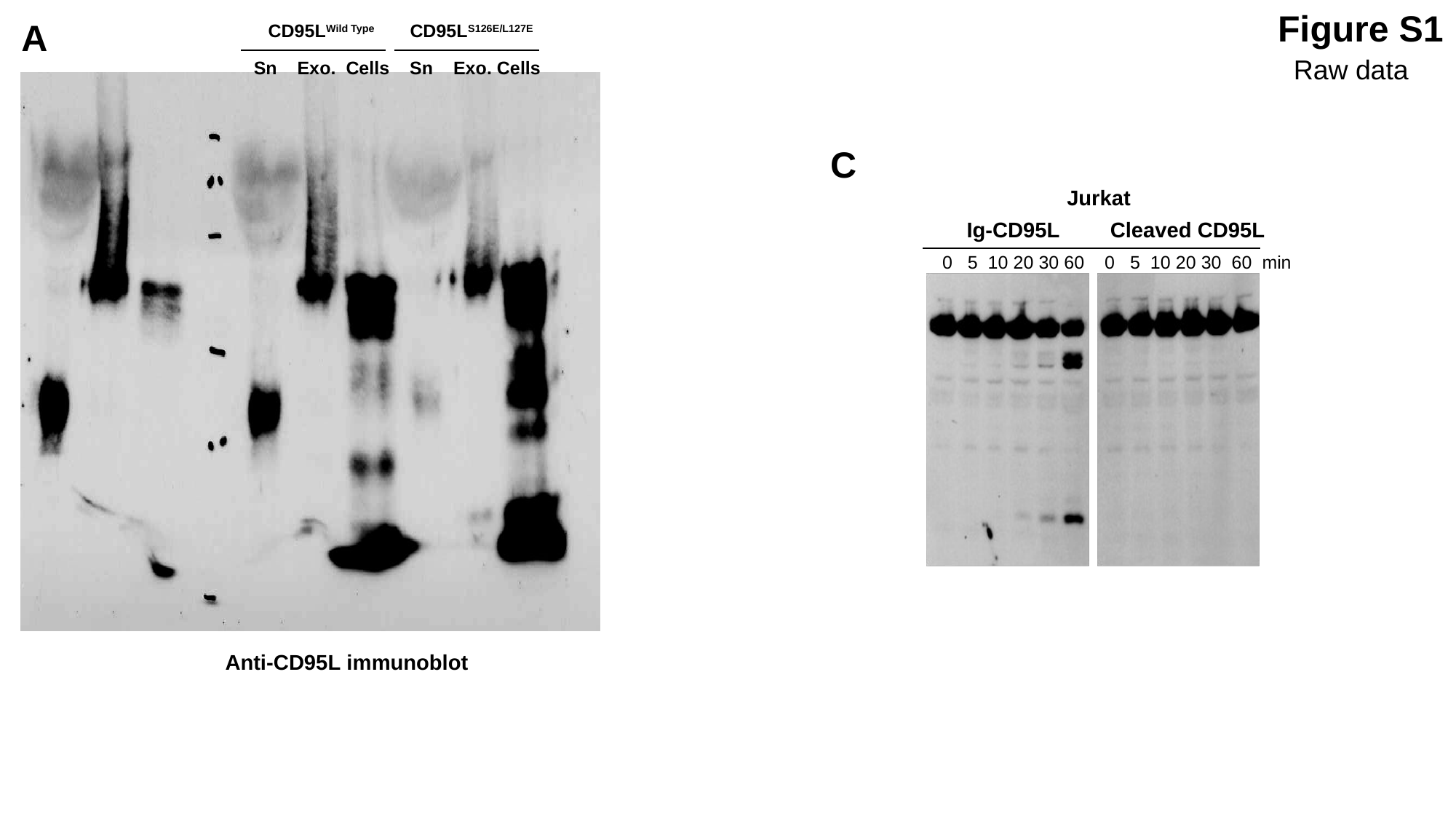

Figure S1
A
CD95LWild Type CD95LS126E/L127E
Raw data
Sn Exo. Cells Sn Exo. Cells
C
Jurkat
Ig-CD95L
Cleaved CD95L
0 5 10 20 30 60 0 5 10 20 30 60 min
Anti-CD95L immunoblot

## Slide 8
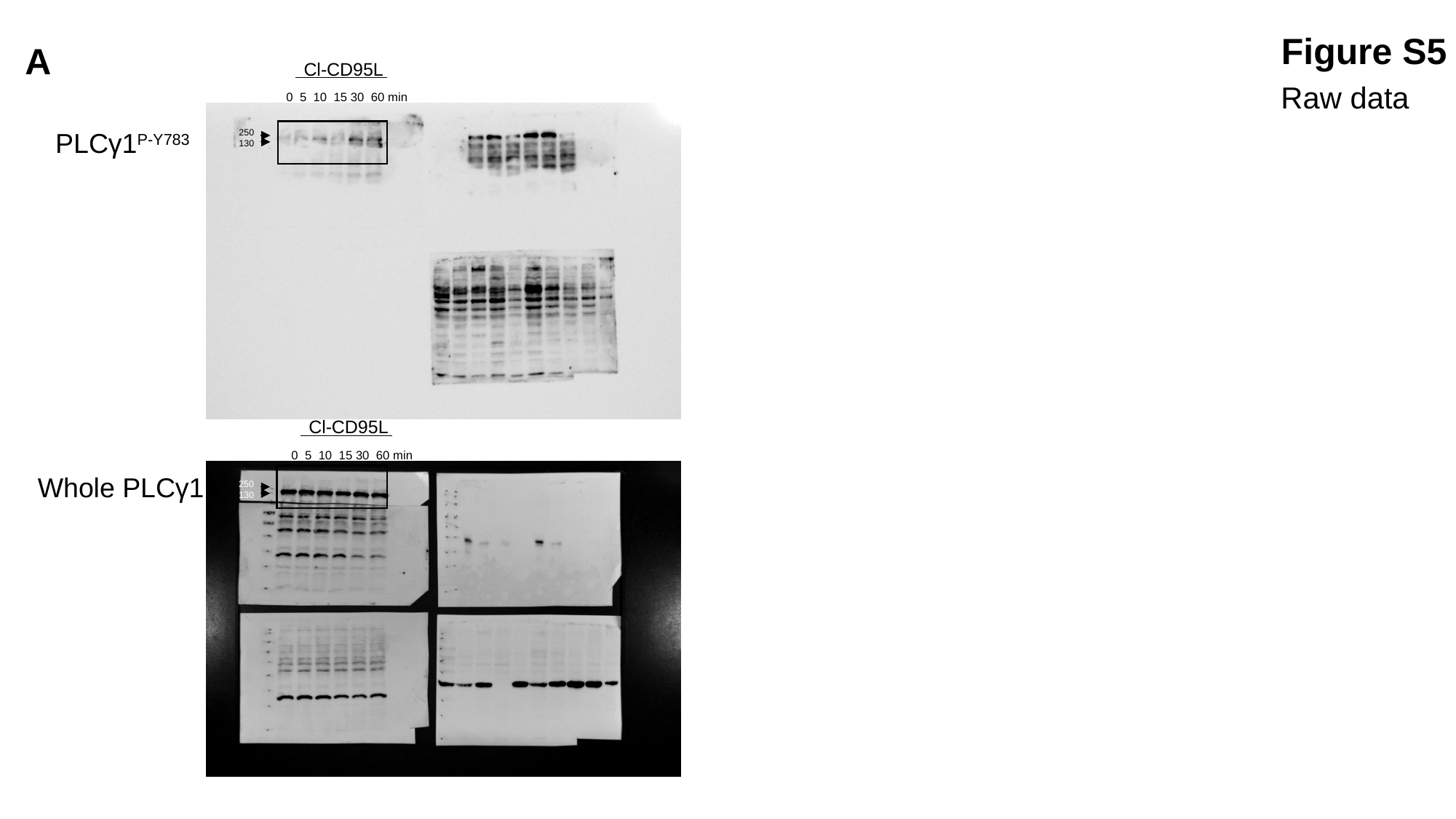

Figure S5
A
Cl-CD95L
Raw data
0 5 10 15 30 60 min
PLCγ1P-Y783
250
130
Cl-CD95L
0 5 10 15 30 60 min
Whole PLCγ1
250
130

## Slide 9
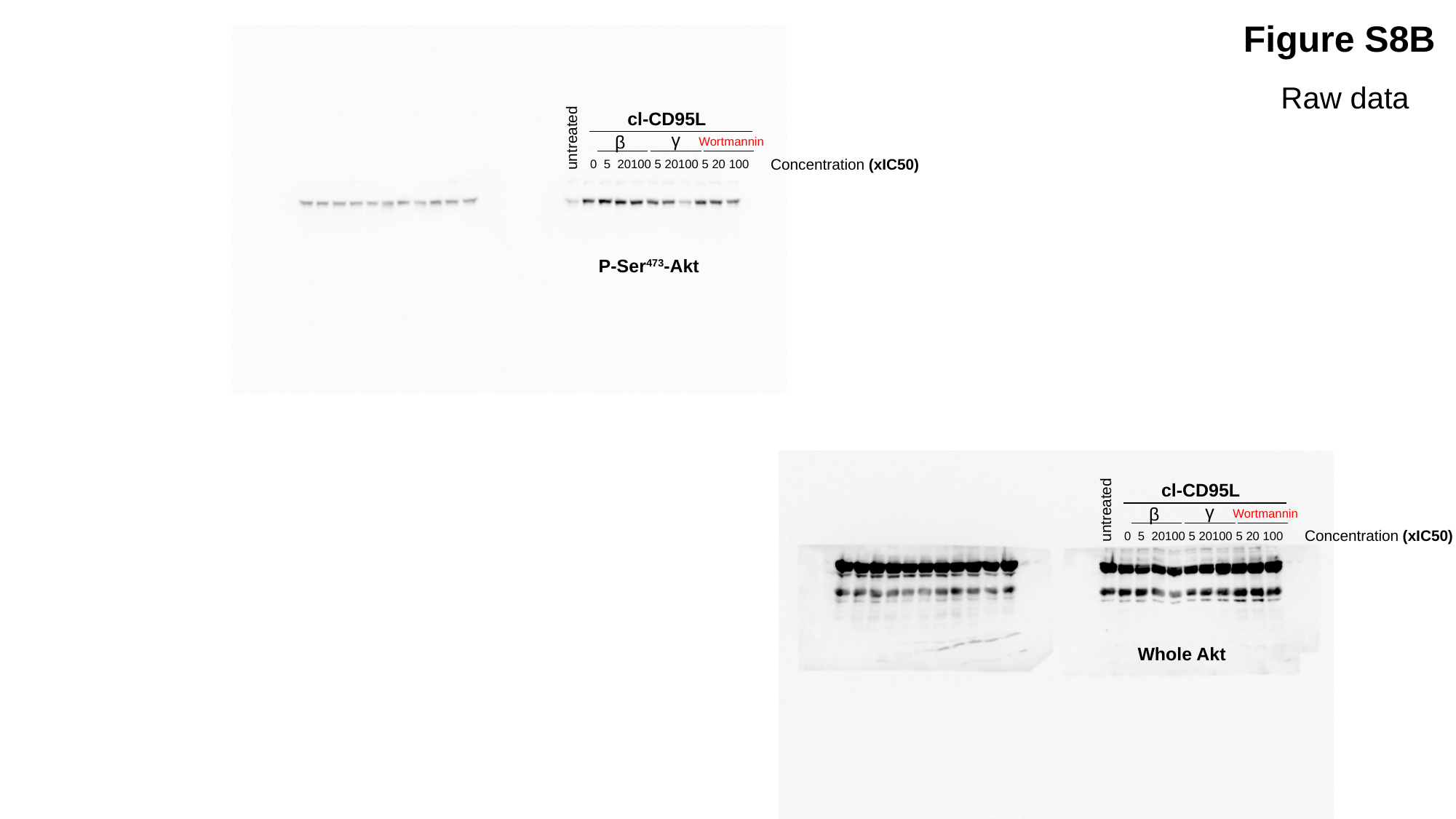

Figure S8B
Raw data
cl-CD95L
γ
untreated
β
Wortmannin
Concentration (xIC50)
0 5 20100 5 20100 5 20 100
P-Ser473-Akt
cl-CD95L
γ
untreated
β
Wortmannin
Concentration (xIC50)
0 5 20100 5 20100 5 20 100
Whole Akt

## Slide 10
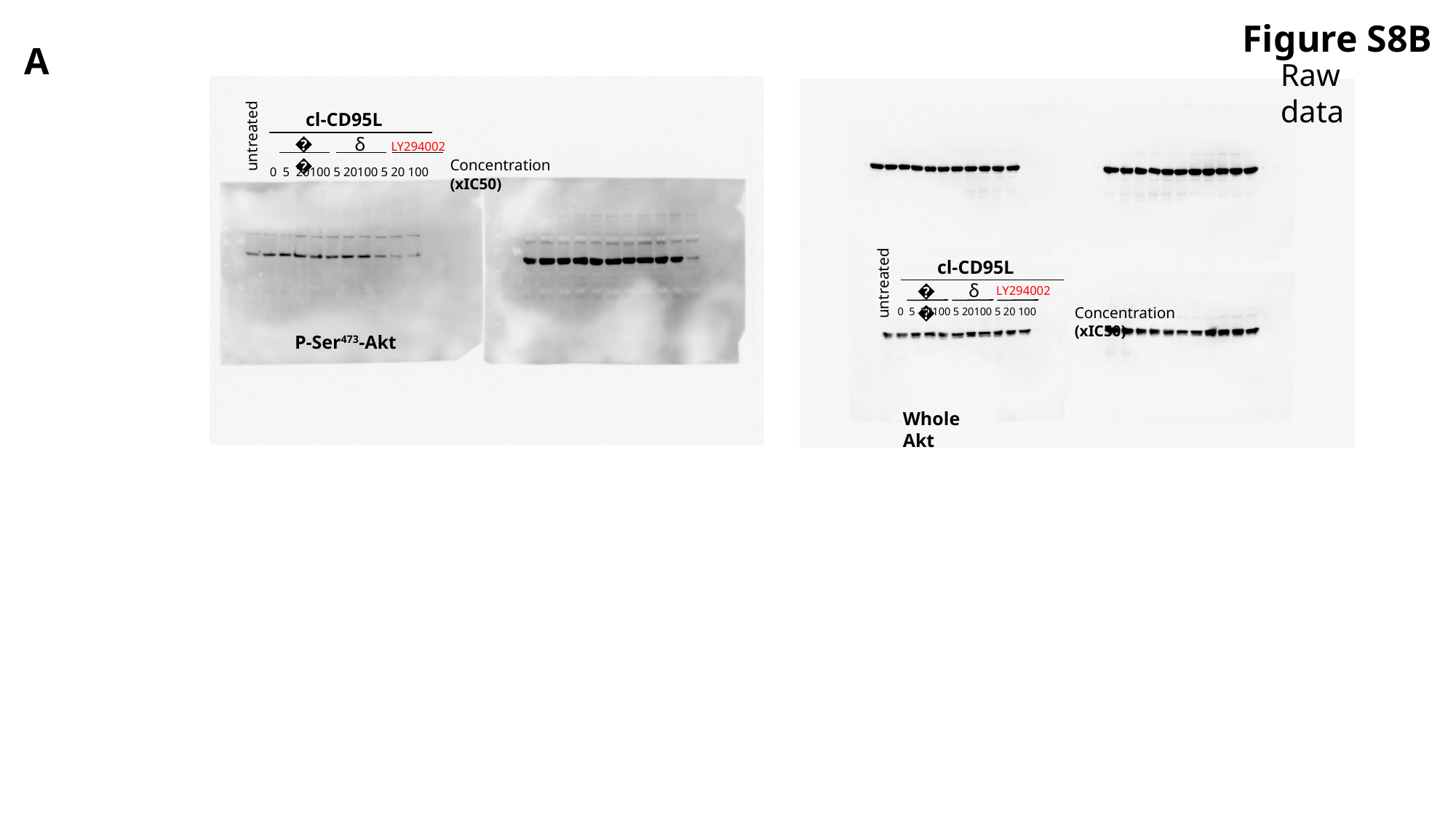

Figure S8B
A
Raw data
cl-CD95L
untreated
𝛂
δ
LY294002
Concentration (xIC50)
0 5 20100 5 20100 5 20 100
cl-CD95L
untreated
δ
𝛂
LY294002
0 5 20100 5 20100 5 20 100
Concentration (xIC50)
P-Ser473-Akt
Whole Akt

## Slide 11
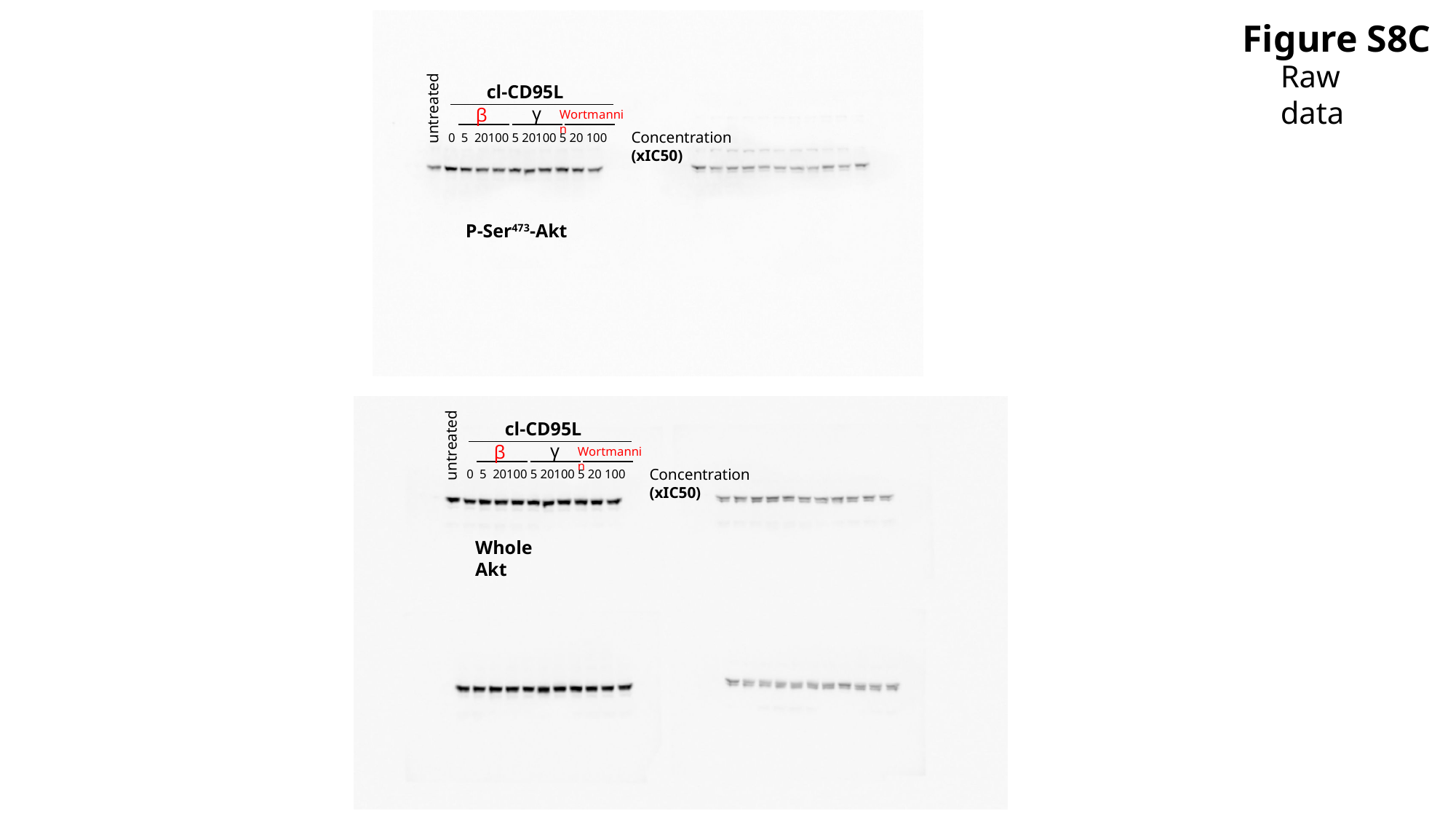

Figure S8C
Raw data
cl-CD95L
untreated
γ
β
Wortmannin
Concentration (xIC50)
0 5 20100 5 20100 5 20 100
P-Ser473-Akt
cl-CD95L
untreated
γ
β
Wortmannin
Concentration (xIC50)
0 5 20100 5 20100 5 20 100
Whole Akt

## Slide 12
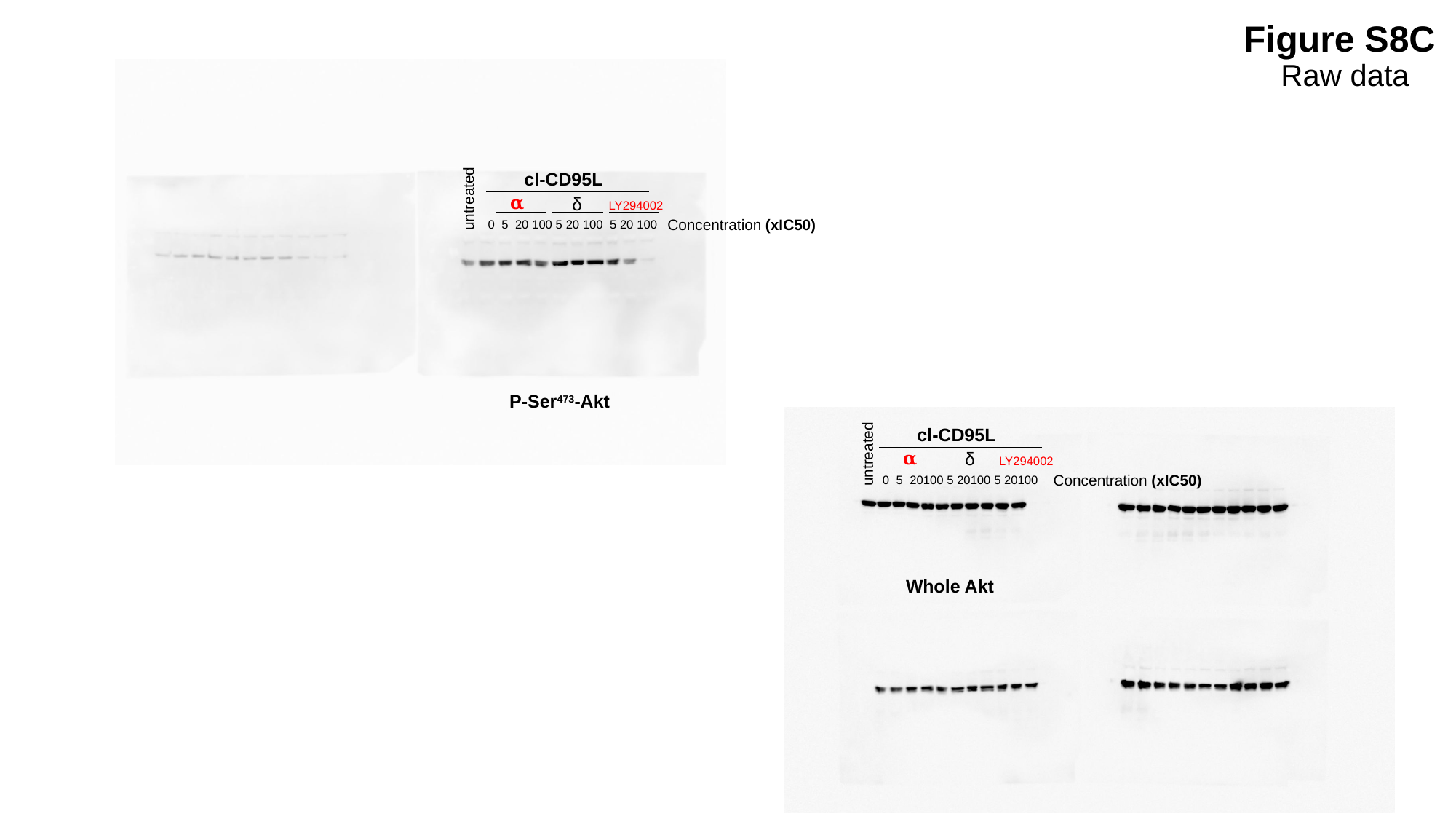

Figure S8C
Raw data
cl-CD95L
untreated
𝛂
δ
LY294002
Concentration (xIC50)
0 5 20 100 5 20 100 5 20 100
P-Ser473-Akt
cl-CD95L
untreated
𝛂
δ
LY294002
Concentration (xIC50)
0 5 20100 5 20100 5 20100
Whole Akt
