# Supplementary material for: Correction: The Naturally Processed CD95L Elicits a c-Yes/Calcium/PI3K-Driven Cell Migration Pathway
Source: PLoS Biol. 2023 Feb 23;21(2):e3002027. doi: 10.1371/journal.pbio.3002027 (PMC9949890; doi:10.1371/journal.pbio.3002027)
Supplement: S2 File — (DOCX) [file pbio.3002027.s012.docx]

**File S2: Supplementary information for Materials and Methods section.**

**Protocol for generation of control and CD95-expressing PS120-NHE1 cells in PLoS Biology.**

The transmembrane transporter Na(+)/H(+) exchanger isoform 1 (NHE1) regulates intracellular pH and is ubiquitously expressed [1]. The original PS120 cell line was derived from the Chinese Hamster fibroblast CCL39 cell line and is devoid of all NHE isoforms [2]. This cell line has been genetically selected using a H^+^ killing technique by Jacques Pouyssegurs’ group [3]. This cell line is used as a recipient for WT and mutated NHE isoforms.

We used the pECE vector encoding NHE1, described in [4]. When transfected, this vector expresses NHE1 at a moderate level (SV40 promoter). Stably transfected PS120 cells (NHE-deficient cells) were selected by using repeated acute acidification (NH_4_^+^ pre-pulse technique) in which only NHE1-expressing cells can survive (see [5] for the method). To avoid clonal differences due to insertion effects, we selected a cell population expressing NHE1. The initial pECE-derived vector contained only the WT NHE1 and, we next electroporated the NHE1-reconstituted PS120 cell population (designated PS120 control cells in the manuscript) using BTM 830 electroporation generator (BTX, Holliston, MA) with the empty vector (pcDNA3.1(+)), a wild type human CD95 or its death domain truncated (Δ1-210) counterpart encoding-pcDNA3 vector. After 24 hours, the medium was replaced, and cells were incubated for 7 days in the presence of neomycin (1 mg/mL). CD95-expressing PS120 cells were next cloned by limiting dilutions and the CD95 expression was assessed using flow cytometry. Control cells corresponded to PS120 cells (PS120-NHE1-pcDNA3.1(+)), and CD95-expressing counterparts were designated PS120^-CD95^) (NHE1- and human full length CD95-expressing PS120 cells) and PS120^CD95(Δ1-210)^ (NHE1- and human death domain truncated CD95-expressing PS120 cells).

**Protocol for the transfection of Lifeact-GFP, PHAkt-GFP and GFP-Orai1.**

The T cell lines H9 and Jurkat were electroporated at 200V/65 ms using the BTM 830 electroporation generator (BTX, Holliston, MA) with 10 μg of the vectors, pmEGFP-N1-Lifeact (gift from Dr R. Wedlich-soeldner, University of Münster, Germany), PHAkt-eGFP_pEGFP-N1 (gift from T. Balla, National Institutes of Health, Bethesda, USA) and GFP-Orai1 (described in [6]). 24 hours after transfection, living cells were harvested in a Ficoll gradient and stimulated in the presence or absence of cl-CD95L.

**Protocol for the production of Ig-CD95.**

HEK/293T cells maintained in an 8% Fetal calf serum (FCS)-containing medium were transfected using Calcium/Phosphate precipitation method with 3 μg of empty plasmid (control pcDNA3.1(+)), wild type human CD95L (full length CD95L cleaved by metalloproteases – cl-CD95L) or IgCD95L [7]-containing vector. 24 hrs after transfection, medium was replaced by OPTI-MEM (Invitrogen) supplemented with 2 mM L-glutamine. After 5 days, media containing metalloprotease-cleaved CD95L (cl-CD95L), IgCD95L or control (pcDNA3.1(+)) were harvested. Dead cells and debris were eliminated through a first step of centrifugation (2 x 4000 rpm for 15 min) followed by a second step, for the elimination of exosomes by ultracentrifugation at 100,000 g for 2hrs. Control medium served as untreated condition.

**Information regarding CEM and SKW6.4 cell lines.**

CEM is a T cell line purchased from ATCC ([CCRF CEM] CCL-119™). SKW 6.4 is a B-cell line purchased from ATCC (TIB-215™). These cells have been extensively characterized for the CD95-mediated signaling pathways [8-10]. These cells were cultured in RPMI medium complemented with 8% fetal calf serum.

**Protocol for PLCy1-reconsistuted Jurkat cells.**

The PLCγ1-deficient jurkat cell line (J.gamma1, CRL-2678™) and its PLCγ1-reconstituted counterpart (J.gamma1.WT derivative ATCC CRL-2679) were obtained from ATCC. These cells have been cloned from E6-1 Jurkat cells treated with the frameshifting mutagen ICR-191 and clones were selected based on their inability to trigger a calcium response in response to pervanadate [11].

**Sequences for all shRNAs, including scrambled.**

As mentioned in the manuscript, the silencing experiments were performed by lentiviral transduction using ready to use and validated shRNAmir-pGIPZ vectors for c-yes, Orai1, or the recommended nontargeting shRNAmir-pGIPZ vector as a negative control following the manufacturer’s recommendation (Open Biosystems, USA). To improve the percentage of transduced T-cells, living cells were harvested 72 h after transduction and green cells (pGIPZ encodes GFP) were sorted by flow cytometry using FACSAria (BD Bioscience). These tools are validated shRNAmir-pGIPZ-vectors for c- Yes, Orai1 and a non-targeting shRNAmir-pGIZ vector as a negative control.

The c-yes-targeting mature sequences for these vectors were: TTTGGTCCCATGATATTCC (clone id V2LHS_69603); TAAGATTGCATACTGACTG (clone id V2LHS_69601); GCAAGGTTAATTGAAGACA (clone id V3LHS_345521)

The c-Orai1-targeting mature sequences for these vectors were: CAGCTAGGAAGAGCAGCGT (clone id V3LHS_340965); TATGTGTCACACACACATG (clone id V3LHS_405711)

The sequences used for the GIPZ non-silencing lentiviral ShRNA control vector corresponded to this (<https://horizondiscovery.com/en/gene-modulation/knockdown/shrna/products/gipz-lentiviral-shrna-controls#description>).

**Suppliers for PI3K isoform selective inhibitors, zVAD.**

The different isoform-selective inhibitors on the class I PI3Ks came from Calbiochem (Merck Chemicals Ltd., Nottingham, UK).

PI3-Kα Inhibitor IV (3-(4 Morpholinothieno[3,2-d]pyrimidin-2-yl)phenol) - CAS 1188890-32-5; PI3-Kβ Inhibitor VI/TGX-221 ((±)-7-Methyl-2-(morpholin-4-yl)-9-(1-phenylaminoethyl)-pyrido[1,2-a]-pyrimidin-4-one) - CAS 663619-89-4; PI 3-Kγ Inhibitor (5-Quinoxalin-6-ylmethylene-thiazolidine-2,4-dione) CAS 648450-29-7; PI 3-Kδ Inhibitor X, IC87114- CAS 371242-69-2; Wortmannin - CAS 19545-26-7; LY 294002 - CAS 154447-36-6

zVAD-fmk (carbobenzoxy-valyl-alanyl-aspartyl-[O-methyl]-fluoromethylketone) was purchased from Calbiochem (Merck Chemicals Ltd., Nottingham, UK).

**References**

1. Sardet C, Franchi A, Pouyssegur J. Molecular cloning, primary structure, and expression of the human growth factor-activatable Na+/H+ antiporter. Cell. 1989;56(2):271-80. Epub 1989/01/27. doi: 10.1016/0092-8674(89)90901-x. PubMed PMID: 2536298.

2. Milosavljevic N, Monet M, Lena I, Brau F, Lacas-Gervais S, Feliciangeli S, et al. The intracellular Na(+)/H(+) exchanger NHE7 effects a Na(+)-coupled, but not K(+)-coupled proton-loading mechanism in endocytosis. Cell reports. 2014;7(3):689-96. doi: 10.1016/j.celrep.2014.03.054. PubMed PMID: 24767989.

3. Pouyssegur J, Sardet C, Franchi A, L'Allemain G, Paris S. A specific mutation abolishing Na+/H+ antiport activity in hamster fibroblasts precludes growth at neutral and acidic pH. Proc Natl Acad Sci U S A. 1984;81(15):4833-7. PubMed PMID: 6087349; PubMed Central PMCID: PMC391585.

4. Wakabayashi S, Fafournoux P, Sardet C, Pouyssegur J. The Na+/H+ antiporter cytoplasmic domain mediates growth factor signals and controls "H(+)-sensing". Proc Natl Acad Sci U S A. 1992;89(6):2424-8. Epub 1992/03/15. doi: 10.1073/pnas.89.6.2424. PubMed PMID: 1372444; PubMed Central PMCID: PMCPMC48670.

5. Franchi A, Perucca-Lostanlen D, Pouyssegur J. Functional expression of a human Na+/H+ antiporter gene transfected into antiporter-deficient mouse L cells. Proc Natl Acad Sci U S A. 1986;83(24):9388-92. Epub 1986/12/01. doi: 10.1073/pnas.83.24.9388. PubMed PMID: 3025840; PubMed Central PMCID: PMCPMC387143.

6. Khadra N, Bresson-Bepoldin L, Penna A, Chaigne-Delalande B, Segui B, Levade T, et al. CD95 triggers Orai1-mediated localized Ca2+ entry, regulates recruitment of protein kinase C (PKC) beta2, and prevents death-inducing signaling complex formation. Proc Natl Acad Sci U S A. 2011;108(47):19072-7. Epub 2011/11/09. doi: 10.1073/pnas.1116946108. PubMed PMID: 22065776; PubMed Central PMCID: PMC3223456.

7. Daburon S, Devaud C, Costet P, Morello A, Garrigue-Antar L, Maillasson M, et al. Functional characterization of a chimeric soluble Fas ligand polymer with in vivo anti-tumor activity. PLoS One. 2013;8(1):e54000. doi: 10.1371/journal.pone.0054000. PubMed PMID: 23326557; PubMed Central PMCID: PMC3541234.

8. Kischkel FC, Hellbardt S, Behrmann I, Germer M, Pawlita M, Krammer PH, et al. Cytotoxicity-dependent APO-1 (Fas/CD95)-associated proteins form a death-inducing signaling complex (DISC) with the receptor. Embo J. 1995;14(22):5579-88. PubMed PMID: 8521815.

9. Peter ME, Hellbardt S, Schwartz-Albiez R, Westendorp MO, Walczak H, Moldenhauer G, et al. Cell surface sialylation plays a role in modulating sensitivity towards APO-1-mediated apoptotic cell death. Cell Death Differ. 1995;2(3):163-71. Epub 1995/07/01. PubMed PMID: 17180039.

10. Trauth BC, Klas C, Peters AM, Matzku S, Moller P, Falk W, et al. Monoclonal antibody-mediated tumor regression by induction of apoptosis. Science. 1989;245(4915):301-5. PubMed PMID: 2787530.

11. Irvin BJ, Williams BL, Nilson AE, Maynor HO, Abraham RT. Pleiotropic contributions of phospholipase C-gamma1 (PLC-gamma1) to T-cell antigen receptor-mediated signaling: reconstitution studies of a PLC-gamma1-deficient Jurkat T-cell line. Mol Cell Biol. 2000;20(24):9149-61. Epub 2000/11/30. doi: 10.1128/MCB.20.24.9149-9161.2000. PubMed PMID: 11094067; PubMed Central PMCID: PMCPMC102173.

12. Kleber S, Sancho-Martinez I, Wiestler B, Beisel A, Gieffers C, Hill O, et al. Yes and PI3K bind CD95 to signal invasion of glioblastoma. Cancer Cell. 2008;13(3):235-48. Epub 2008/03/11. doi: S1535-6108(08)00043-3 [pii]

10.1016/j.ccr.2008.02.003. PubMed PMID: 18328427.
